# Isoforskolin modulates AQP4-SPP1-PIK3C3 related pathway for chronic obstructive pulmonary disease via cAMP signaling

**DOI:** 10.1186/s13020-023-00778-w

**Published:** 2023-10-10

**Authors:** Haochang Lin, Sha Cheng, Songye Yang, Qian Zhang, Lueli Wang, Jiangya Li, Xinyue Zhang, Liju Liang, Xiaoqian Zhou, Furong Yang, Jingfeng Song, Xue Cao, Weimin Yang, Zhiying Weng

**Affiliations:** 1 School of Pharmaceutical Science and Yunnan Key Laboratory of Pharmacology for Natural Products, Kunming Medical University, No. 1168, Chunrong West Road, Yuhua Street, Chenggong New Town, Kunming, 650500 China; 2 State Key Laboratory of Functions and Applications of Medicinal Plants, Guizhou Medical University, Guiyang, 550014 Guizhou China; 3 Key Laboratory of Chemistry for Natural Products of Guizhou Province and Chinese Academy of Sciences, Guiyang, 550014 Guizhou China; 4 Department of Laboratory Animal Science, Kunming Medical University, Kunming, 650500 China

**Keywords:** ISOF, COPD, cAMP, Anti-inflammation, AQP4-SPP1-PIK3C3 signaling

## Abstract

**Background:**

Cyclic adenosine monophosphate (cAMP) levels are directly activated by adenylate cyclase (AC) and play an anti-inflammatory role in chronic obstructive pulmonary disease (COPD). Previously, we have shown that isoforskolin (ISOF) can effectively activate AC1 and AC2 in vitro, improve pulmonary ventilation and reduce the inflammatory response in COPD model rats, supporting that ISOF may be a potential drug for the prevention and treatment of COPD, but the mechanism has not been explored in detail.

**Methods:**

The potential pharmacological mechanisms of ISOF against COPD were analyzed by network pharmacology and multi-omics based on pharmacodynamic study. To use specific agonists, inhibitors and/or SiRNA for gene regulation function studies, combined qPCR, WB were applied to detect changes in mRNA and protein expression of important targets PIK3C3, AKT, mTOR, SPP1 and AQP4 which related to ISOF effect on COPD. And the key inflammatory factors detected by ELISA.

**Results:**

Bioinformatics suggested that the anti-COPD pharmacological mechanism of ISOF was related to PI3K-AKT signaling pathway, and suggested target protein like PIK3C3, AQP4, SPP1, AKT, mTOR. Using the AQP4 inhibitor,or inhibiting SPP1 expression by siRNA-SPP1 could block the PIK3C3-AKT-mTOR pathway and ameliorate chronic inflammation. ISOF showed cAMP-promoting effect then suppressed AQP4 expression, together with decreased level of IL-1β, IL-6, and IL-8.

**Conclusions:**

These findings demonstrate ISOF controlled the cAMP-regulated PIK3C3-AKT-mTOR pathway, thereby alleviating inflammatory development in COPD. The cAMP/AQP4/PIK3C3 axis also modulate Th17/Treg differentiation, revealed potential therapeutic targets for this disease.

**Supplementary Information:**

The online version contains supplementary material available at 10.1186/s13020-023-00778-w.

## Introduction

COPD kills over 3 million people worldwide each year and has become a highly prevalent disease worldwide, with a prevalence of 8% to 13% in people over 40 years of age and over 20% in people over 60 years of age [5, 36]. COPD is characterized by persistent airflow limitation, and an enhanced chronic inflammatory response in the airways is the main pathological mechanism [20]. Chronic cough, chest pain, dyspnea, physical exercise intolerance, excessive sputum and hypersecretion, depression and weight loss are the most common complaints [20]. The inflammatory response is one of the central mechanisms of COPD, although the pathogenesis is not fully understood. Inflammation is characterised by increased numbers of alveolar macrophages, neutrophils, T lymphocytes, and innate lymphocytes recruited from the circulation. A variety of pro-inflammatory mediators, including cytokines, chemokines, growth factors and lipid mediators, are secreted by these cells and by structural cells, including epithelial and endothelial cells and fibroblasts [20].

ISOF (Compound CID: 9549169), a forskolin derivative, is the only class of naturally occurring compounds that has been shown to directly agitate AC enzymes and increase cAMP levels in a variety of tissues, thereby participating in the regulation of cellular functions, as reported by us [47]. It, a major active compound isolated from Coleus forskohlii, has a promising anti-inflammatory effect and enhances lung function in cigarette smoke-induced COPD rat model, and may contribute to maintaining the balance of Th17/Treg, engage in the differentiation of T cell subsets, and serve a role in maintaining immune homeostasis. It is speculated that ISOF may regulate mTOR expression via cAMP to be involved in T cell subpopulation differentiation, maintenance of Th17/Treg homeostasis and anti-inflammatory effects. However, the pharmacological mechanisms of how ISOF elevates cAMP in COPD to exert anti-inflammatory and improve lung function effects, and how it reduces mTOR expression to keep T cell subpopulation homeostasis have not been investigated in detail.cAMP is an attractive drug target for the treatment of chronic airway diseases because it is one of the most important second messengers and plays a key role in relaxing airway smooth muscle and reducing inflammation [52]. The production of cAMP is triggered by the activation of membrane receptors (mainly G protein-coupled receptors), which activate cellular ACs to convert ATP to cAMP [52], where the elevation of cAMP can be achieved either directly by agonizing ACs or indirectly by inhibiting phosphodiesterases [48]. Recent studies have reported that cAMP regulates the mTOR pathway possibly through protein kinase A (PKA) [48], but there is insufficient evidence confirming the role of this regulatory mechanism in the inflammatory response or even in T cell subsets. These events downstream of cAMP include the activation of cyclic nucleotide-gated ion channels [19], exchange proteins directly activated by cAMP (Epac) [8], or PKA. In addition, PKA phosphorylates and controls the activity of cellular motor proteins, ion channels, and enzymes such as protein kinase C, phosphoinositide 3 kinase (PI3K), and phospholipase C [39]. cAMP governs different processes during inflammation and resolution, depending on the activated cell type and pathway. So, the pharmacological mechanism of how ISOF maintains Th17/Treg homeostasis and immune homeostasis through cAMP-regulated mTOR deserves further exploration.

**Fig. 1:**
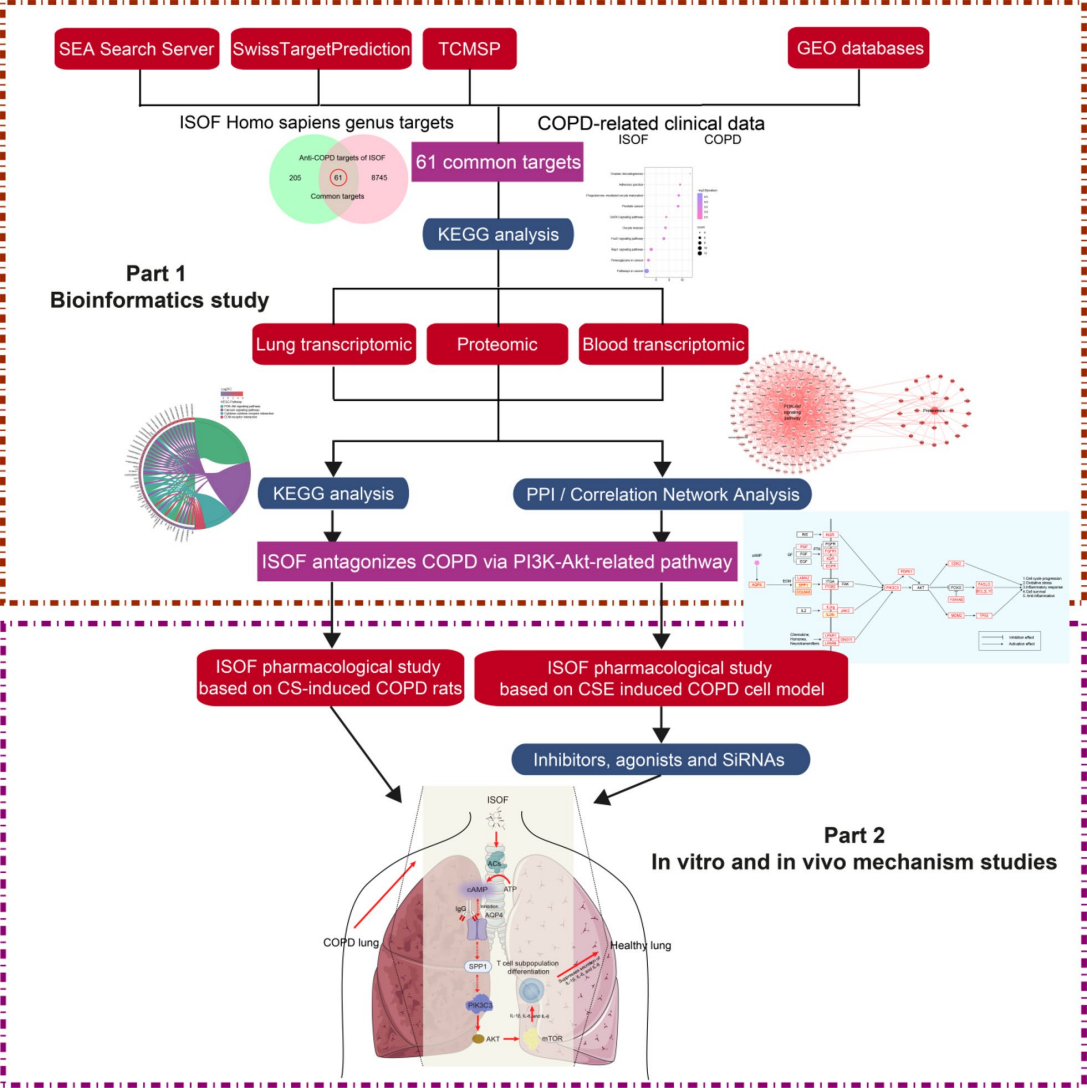
Research process diagram

In this study, network pharmacology combined with multi-omics analysis, and in vivo and in vitro experiments were applied to explore the pharmacological mechanism of ISOF for the treatment of COPD. It provides directions for the ACs/cAMP pathway for the treatment of COPD and also provides a scientific and theoretical basis for the development of ISOF, a new drug candidate for COPD. The specific research process is shown in Fig. 1.

## Methods

### Reagents

ISOF (purity > 99.9%) was synthesized by Baselia Pharmaceuticals (China) according to the patented method for ISOF synthesis (ZL201610044817.8). FSK, SQ22536, TGN20, SAR405 were from MedChemExpress (USA). Cigarettes (Hongqiqu® filter cigarettes containing 11 mg tar, 0.7 mg nicotine and 13 mg carbon monoxide per cigarette) were purchased from Henan Tobacco Industry (China). Human species IL-1β, IL-6 and IL-8 ELISA kits (KE00021, KE00129, KE00006) and protein marker (PL00003) were obtained from Proteintech (USA). Human Species cAMP ELISA Kit (K019-H1) was from ARBOR ASSAYS (USA). Lysis buffer (REF: P0013B) was purchased from Beyotime (Shanghai, China). Cocktail (P8340), PMSF (10837091001), PhosStop (4906845001) were from Sigma (USA). Liposome Transfection 3000 (L3000-001) was from Thermo (USA). All other chemicals were reagent grade and obtained from the market. The following antibodies were used in the study: rabbit mTOR antibody (Cat. #2972), rabbit phosphorylated (p) mTOR (Ser2481) antibody (Cat. #2974), rabbit p-mTOR (Ser2448) antibody (Cat. #2971), rabbit AKT antibody (Cat. #9272), rabbit p-AKT (Ser473) antibody (Cat. #4060), PIK3C3 (D9A5) rabbit mAb (#4263), GAPDH (14C10) rabbit mAb (Cat. #2118) from Cell signaling Technology (USA). AQP4 rabbit-derived primary antibody (ab46182), and SPP1 rabbit-derived primary antibody (ab8448) were purchased from abcam (UK). Membranes were stripped and reprobed with Western primary antibody and secondary antibody removal solution (weakly basic, P0025B, Beyotime, China). Primers were obtained from Bioengineering (Shanghai, China). SiRNA was obtained from Gima Genetics (Shanghai, China).

### Network pharmacology

SMILES for ISOF were obtained through the PubChem database [13] (http://www.swisstargetprediction.ch/). Its SMILES was imported into SwissTargetPrediction [13] (http://www.swisstargetprediction.ch/) with SEA Search Server (https://sea.bkslab.org/) [18] website, as well as 3D structures, were imported to the PharmMapper website [45] (http://www.lilab-ecust.cn/pharmmapper/) to obtain the respective relevant targets. The screening criteria were Probability ≥ 0.6, Max Tc ≥ 0.6 and Norm Fit ≥ 0.6. All targets were converted into Gene symbol format, duplicates were excluded and merged to obtain the respective relevant targets. A search through the GEO database [2] (https://www.ncbi.nlm.nih.gov/gds/?term =), using COPD, Chronic obstructive pulmonary disease, etc. as search terms, yielded GSE37768, GSE73395 [34], GSE37768, GSE103174, GSE106986, GSE112260 [32], GSE112811, GSE76925 [27] and GSE130928 [29] were obtained as annotated files of gene expression from eight independent studies of human alveolar lavage fluid, macrophages, whole blood, and lung tissue, covering non-smoking normal subjects, non-smoking COPD patients, smoking normal subjects, and smoking COPD patients. Differentially expressed genes (DEGs) between smoking normal subjects and smoking COPD patients, non-smoking normal subjects and non-smoking COPD patients, female normal group and female COPD patient group and male normal group and male COPD patient group were screened with *P*-value ≤ 0.05 and |Log_2_FC|≥ 1 as screening conditions, and duplicates were excluded to obtain COPD-related pathological targets. Finally, the Draw Venn Diagram (http://bioinformatics.psb.ugent.be/webtools/Venn/) website was applied to analyze the co-related targets between COPD and ISOF.

ISOF common targets with COPD were analyzed separately by the DAVID online database [16] (https://david.ncifcrf.gov/tools.jsp) for Kyoto Encyclopedia of Genes and Genomes (KEGG) functional enrichment, where KEGG pathways with *P* ≤ 0.05 were included in the analytical study.

### Cigarette smoke-induced COPD rat model

Sprague–Dawley rats (bought from Charles River, Beijing, China) in half genders were randomly divided into 6 groups: control, model, ROFL (0.5 mg/kg), ISOF (0.5 mg/kg), ISOF (1 mg/kg) and ISOF (2.0 mg/kg) groups (n = 16 in each group). All animal care and experimental protocols were approved by the Animal Experimental Ethical Committee of Kunming Medical University (approval number: KMMU-2018001), and all animals received humane care in compliance with the National Institutes of Health guidelines. Animals were exposed to room air or cigarette smoke once a day for 28 weeks (1 cigarette each rat) in an oral and nasal exposure system (Beijing Huironghe Technology, China). After 14 weeks of exposure to cigarette smoke, the rats were orally treated with ROFL (0.5 mg/kg) or ISOF (0.5, 1.0, or 2.0 mg/kg) in 0.9% saline solution (80%) and PEG400 (20%) once daily for 14 consecutive weeks. Totally 66 animals completed the course of treatments.

### Transcriptomics analysis in lung tissue

In previous studies, COPD model rats were established based on cigarette smoke exposure (CS)-induced rats, and 100 g of lung tissue was taken from three rats in each of the Control, model and ISOF groups [47]. Genes with Fold Change > 1.2 and *P* value < 0.05 by DESeq2 were designated as differentially expressed genes. In addition, GO functional enrichment and KEGG pathway analysis were performed using Goatools with KOBAS and a *P* value ≤ 0.05 was used as a screening criterion. Raw transcriptomics data are available in the Sequence Read Archive (SRA) database (PRJNA685952).

### Blood transcriptomics analysis

Total RNA was extracted from whole blood of rats using TRIZOL reagent (Invitrogen, cat. NO 15596026) according to the instructions. after digestion of DNA with DNaseI. RNA quality was determined by testing A260/A280 with a Nanodrop^™^ OneCspectrophotometer (Thermo Fisher Scientific Inc). the integrity of RNA was confirmed by 1.5% agarose gel electrophoresis. Qualified RNA was finally quantified by Qubit3.0 and QubitTM RNA Wide Range Detection Kit (Life Technologies, Q10210).

Using the KC-Digital^™^ Stranded mRNA Library Prep Kit for Illumina^®^ (Catalog NO. DR08502, Wuhan Seqhealth Co.; Ltd. China), 2 μg of total RNA was used for stranded RNA sequencing library according to the manufacturer's instructions. preparation. The kit eliminates repeat bias in PCR and sequencing steps by using a unique molecular identifier (UMI) of 8 random bases to label pre-amplified cDNA molecules. The equivalent of 200–500 bps of library product is enriched, quantified, and finally sequenced on a Novaseq 6000 sequencer (Illumina), model PE150.

Raw sequencing data were first filtered by Trimmomatic (version 0.36) and low quality reads were discarded. Clean Reads are further processed with an internal script to eliminate duplicate bias introduced in library preparation and sequencing. Briefly, Clean Reads are first clustered based on UMI sequences, where reads with the same UMI sequence are grouped into the same cluster. Reads in the same cluster are compared by pairwise matching, and then reads with more than 95% sequence identity are extracted into a new sub-cluster. After all subclusters were generated, multiple sequence comparisons were performed to obtain one consensus sequence for each subcluster. After these steps, any errors and biases introduced by PCR amplification or sequencing are eliminated.

The consensus sequences with duplicates removed were used for standard transcriptomics analysis. They were mapped to the reference genome of rats from NCBI (GCol_000001895.5, https://www.ncbi.nlm.nih.gov/assembly/GCol_000001895.5/) using STAR software (version 2.5.3a) and default parameters. Reads mapped to exonic regions of each gene were counted by featureCounts (Subread-1.5.1; Bioconductor) and then RPKM was calculated. genes differentially expressed between groups were identified using the edgeR package (version 3.12.1). *P*-value threshold was 0.05 and differential expression (Fold chang) threshold of 1.2 was used to determine the statistical significance of gene expression differences. GO analysis and KEGG enrichment analysis were both implemented by KOBAS software (version: 2.1.1) with a *P*-value threshold of 0.05 to determine the degree of statistical enrichment. Raw transcriptomics data are available in the SRA database (PRJNA744396).

### Proteomics analysis

In previous studies, lung tissues from the control, model and ISOF (1 mg/kg) groups were subjected to proteomic analysis [47]. Quantitative protein results were statistically analyzed by Mann–Whitney Test. *P*-value threshold of 0.05 and a differential expression (Fold change) threshold of 1.2 were used to determine the statistical significance of protein expression differences (DEPs). GO and KEGG analyses were used to analyze functional attribution and signal transduction pathways, and PPI data analysis was also performed through the STRING database was performed. Raw proteomics data are available in the ProteomeXchange (px-submission #440240).

### Correlation network analysis-protein–protein interaction (PPI)

PPI analysis was performed for whole blood transcriptomics of DEGs or proteomics of DEPs or with all targets in the PI3K-AKT signaling pathway through the STRING database [43] (https://string-db.org/), where Homo sapiens was used as the study species and the parameter was set to 0.7 high confidence. Next, the PPI analysis data were imported into Cytoscape [31] to draw a PPI analysis network for visualization.

### Real-time quantitative PCR(RT-PCR)

Total RNA was extracted from rat lung tissue samples and reverse transcribed using a PrimeScript RT kit (Takara). All RT-PCRs were performed on an Applied Biosystems 7500 real-time quantitative PCR system. Data from different samples were normalized using Gapdh as an internal control. The primers used for RT-PCR were showed in Additional file 1: Table S1.

### Western blotting (WB)

Total proteins of rat lung and BEAS2B cells were extracted with RIPA, PMSF, protease inhibitor cocktail and PhosSTOP. Determination of protein concentration by BCA protein detection kit. The total protein (20 μg) was separated by electrophoresis with 8% Sodium lauryl sulfate gel, transfer onto PVDF membrane and incubate at room temperature in a closed buffer for 2 h. The antibody and membrane of AQP4, SPP1, PIK3C3, AKT, p-AKT (S473), mTOR, pmTOR (S2481), p-mTOR (S2448) or GAPDH were cultured overnight at 4 ℃ (The internal reference GAPDH had a strip on both membranes in each experiment, and membranes were cut horizontally and were stripped and reprobed in MTOR phosphorylation). The membrane was cleaned 4 times with 1% TBST and incubated at room temperature for 2 h with goat anti-rabbit IgG and rabbit anti-mouse antibody labeled with Horseradish peroxidase. The membrane is then cleaned four times and tested with a Horseradish peroxidase kit. The image was developed using Amersham Imager 600, and the grayscale of the protein band in the membrane was Quantitative analysis with its analysis function.

### Culture of BEAS2B cells and establishment in vitro

The BEAS2B cells were cultured in a medium containing 10% FBS and 1% penicillin–streptomycin solution in a DMEM medium containing high glucose and maintained in 37 ℃ and 5% CO2 incubators. The chronic inflammatory model of COPD in vitro was established when the cells were exposed to DMEM complete medium containing 4% CSE for 6 h after the cells reached 80% density.

### Preparation of cigarette smoke extract (CSE)

The CSE was prepared with a smoke extraction device, the unfiltered cigarette was placed in a driving device with a 50 mL syringe, and the smoke was continuously aspirated and ignited. The smoke was discharged through the outlet of a three-way pipe into the 20 mL DMEM basal medium to form suspension, the suspension was adjusted to pH 7.4 with 1 mol/L NaOH, and the CSE solution was filtrated by 0.22 μM microporous membrane in the ultra-clean platform. The complete medium containing 10% FBS, 1% penicillin and streptomycin were diluted to 4% CSE for the experiment (freshly prepared use).

### Transfection of SPP1-SiRNA and the addition of inhibitors and agonists in BEAS2B cells

SPP1 SiRNA (SPP1-homo-1491:5ʹ—3ʹ: GGUGGUGUGUCAAUUGCUUAUTT, 3ʹ—5ʹ: AUAAGCAAUUGACCACCTT) was used for transient transfection in the presence of fectamine 3000. The transfected cells were cultured for 48 h.

ISOF, FSK, SQ22536, TGN20 and SAR405 were dissolved in DMSO at concentrations of 100, 25, 10, 5 and 20 μM, respectively. In general, ISOF, FSK, SQ22536, TGN20 and SAR405 were uniformly mixed with 4% CSE in DMEM complete medium, and then BEAS2B cells were incubated for 6 h.

### Detection of IL-1β, IL-6, IL-8 and cAMP by ELISA

ELISA tests for IL-1β, IL-6, and IL-8 were performed according to the manufacturer's instructions of Proteintech, and the cAMP ELISA test was performed according to ARBOR ASSAYS.

### Statistical analysis

All data values are displayed as mean ± SD. Plots were made using GraphPad Prism 8, and statistical analyses were performed using the Student–Newman–Keuls method of one-way ANOVA (the normality test and the equivalent variance test were passed) and the rank sum test of one-way ANOVA (the normal test and/or equivalent variance test, not passed) via SigmaStat 3.5.

## Results

### Bioinformatics suggested that the anti-COPD pharmacological mechanism of ISOF is related to PI3K-AKT signaling pathway

**Fig. 2:**
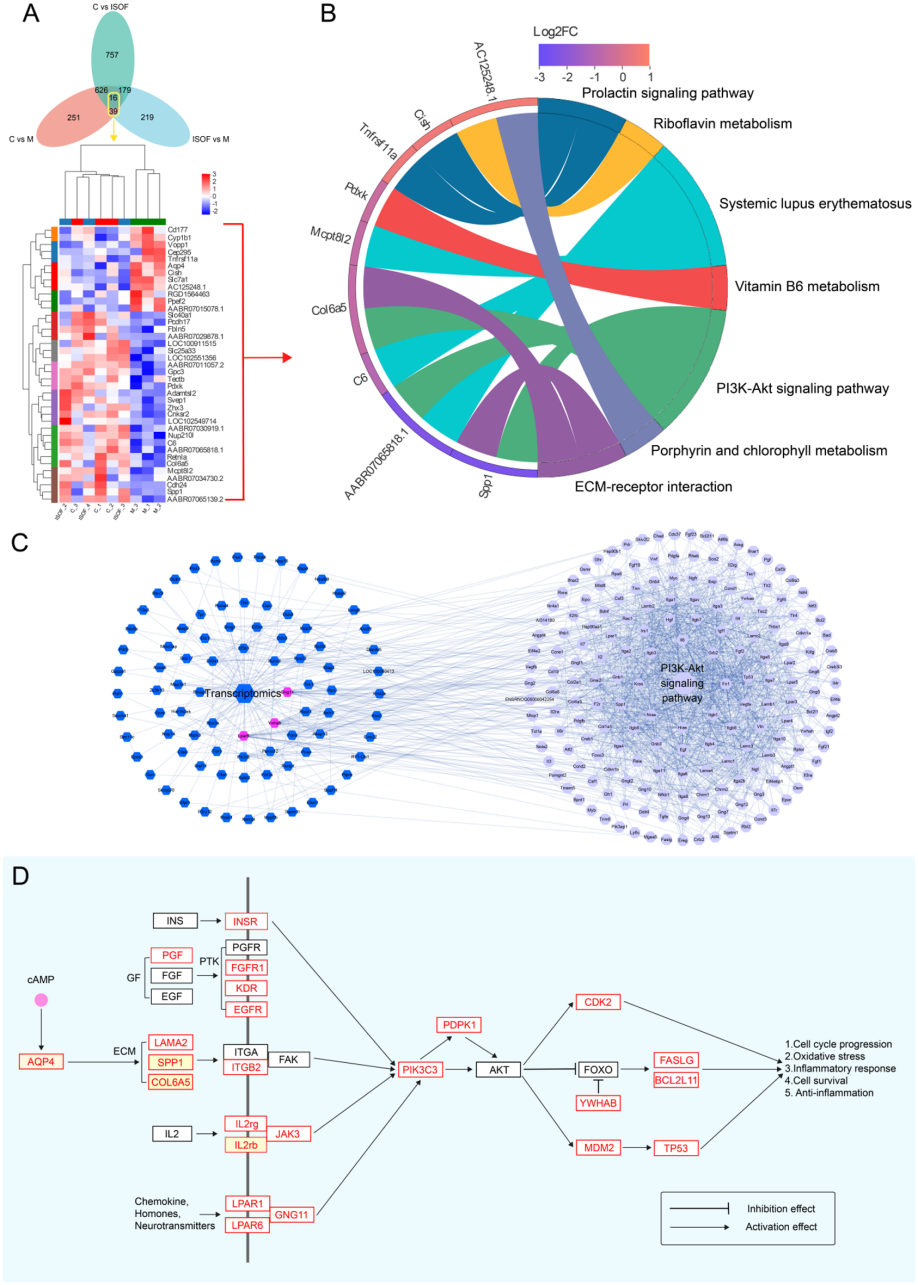
Network pharmacology combined with multi-omics analysis suggests that the anti-COPD pharmacological mechanism of ISOF is mainly related to the PI3K-Akt signaling pathway. **A**: Venn analysis of three comparative DEGs in lung tissue transcriptomics and clustering screening of 55 common DEGs; **B**: KEGG functional enrichment analysis based on 39 core DEGs; **C**: PPI analysis of the correlation between whole blood transcriptomics DEGs and PI3K-Akt signaling pathway targets (light blue positive octagonal nodes represent genes of the PI3K-Akt signaling pathway, dark blue positive hexagonal nodes represent DEGs of ISOF vs M in whole blood transcriptomics, purple positive hexagonal nodes represent genes shared by whole blood transcriptomics and PI3K-Akt signaling pathway); **D**: pharmacological mechanism map according to network pharmacology and multi-omics analysis (red borders are DEGs in multi-omics or ISOF's anti-COPD related targets in network pharmacology, red boxes on a yellow background are core DEGs in histology)

Initially, The impact of ISOF on COPD patients was considered from a clinical perspective by applying network pharmacology, so the appropriate targets related to ISOF (Additional file 1: Table S2) and COPD (Additional file 1: Table S3) were obtained from SwissTargetPrediction, SEA Search Server, PharmMapper website, and GEO database in NCBI (Additional file 1: Figure S1), respectively. KEGG analysis of 61 common targets for ISOF and COPD (Additional file 1: Figure S2) revealed that ISOF may affect COPD patients through 27 pathways (Additional file 1: Table S4). The best significant one (Additional file 1: Figure S3A and Table S4) was Pathway in cancer, specifically, including multiple pathways, such as MAPK signaling pathway, PI3K-AKT signaling pathway, etc., which are closely related to inflammatory immune function [3, 9, 47]. Previous transcriptomic and proteomic data [47] in lung tissue were used to assess the efficacy of ISOF in CS-induced COPD at the transcriptional and protein levels. The KEGG enrichment of 39 ISOF-treated gene sets (COPD pathogenesis altered them, while ISOF treatment reversed them, Fig. 2A) in transcriptome showed that PI3K-AKT signaling pathway and ECM-receptor interaction (two pathways in cancer sub-pathway, Fig. 1B) were significantly implicated in the mechanism of ISOF treatment for CS-induced COPD rats. Further PPI analysis in the ISOF vs COPD DEGs disclosed that the most important *Tp53* and *Il2rb* genes (Additional file 1: Table S4) were involved in the PI3K-AKT signaling pathway, while the ECM-receptor interaction was absent (Additional file 1: Figure S3B, C). Also, proteomic correlation network analysis (Additional file 1: Figure S3E) exhibited similar results as described above. Additionally, transcriptomics based on whole blood tissue (the baseline situation is shown in Additional file 1: Figure S4A–C) were conducted, and analogous conclusions were obtained in the KEGG enrichment results (Additional file 1: Figure S3D) with the correlation analysis network (Fig. 2C). Thus, the PI3K-AKT signaling pathway may be mostly associated with ISOF against COPD. Moreover, based on network pharmacology, multi-omics, and literature search, a relatively more complete picture of the molecular mechanism can be obtained (Fig. 2D).

### ISOF antagonized COPD via PI3K-AKT-related pathway in vitro and in vivo levels

**Fig. 3:**
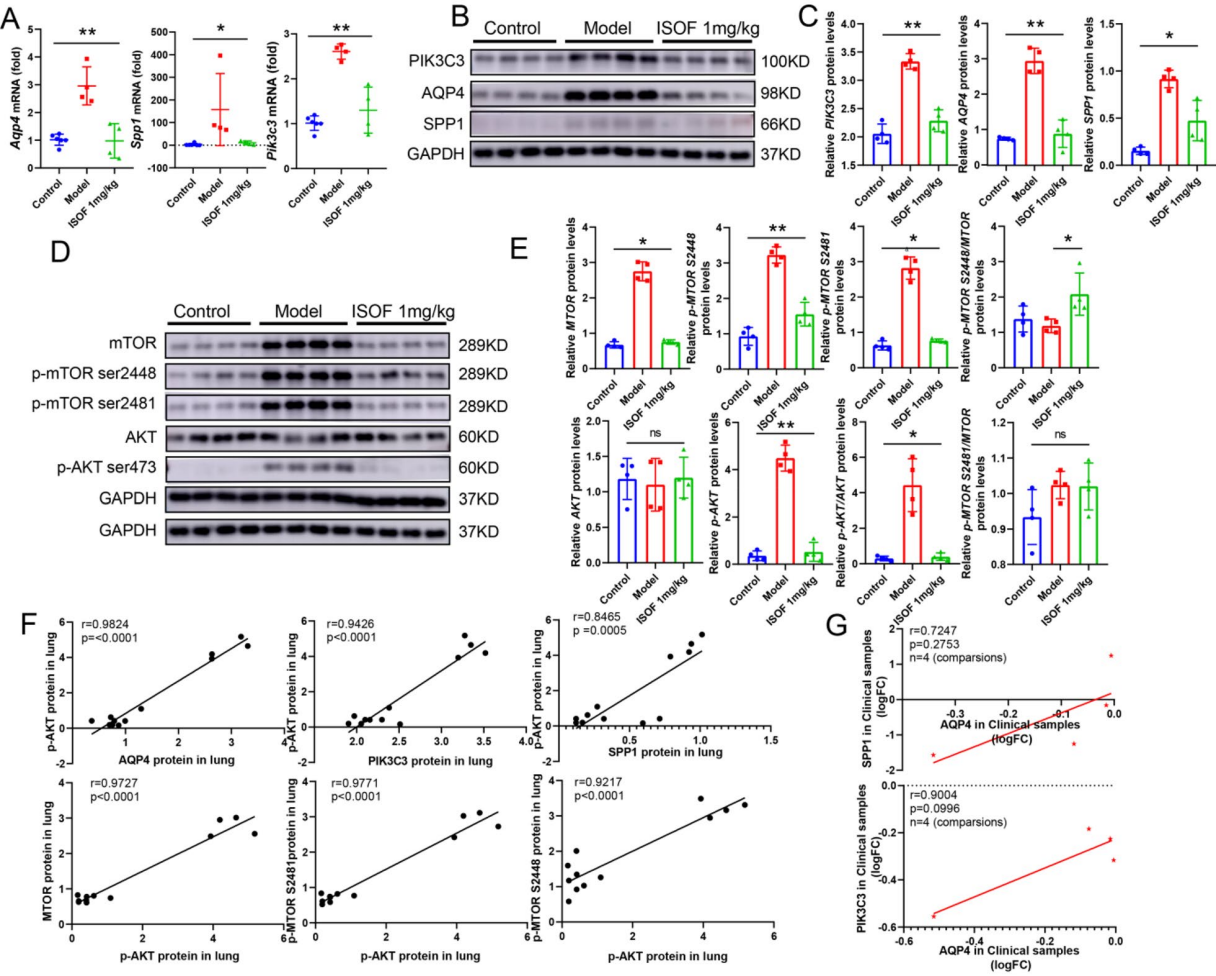
In vivo study of the anti-COPD pharmacological mechanism of ISOF. **A**: Validation of mRNA expression levels of *Pik3c3*, *Aqp4*, and *Spp1*; **B**: Western blot of PIK3C3, AQP4, and SPP1 protein expression in rat lung tissues, n = 4; **C**: Quantification of PIK3C3, AQP4, and SPP1 expression; **D**: Western blot of p-AKT, AKT, mTOR and p-mTOR (S2448 and S2481) protein expression; **E**: Quantification of p-AKT, AKT, MTOR and p-MTOR (S2448 and S2481) expression; **F**: Correlation analysis of p-AKT protein expression on PIK3C3, AQP4, SPP1, AKT, MTOR and p-MTOR protein expression, n = 12; **G**: Correlation analysis of AQP4 (DEGs) on PIK3C3 and SPP1 in expression profiling by array with clinical samples, n = 4. GAPDH was homogenized as an internal reference, *P < 0.05, and **P < 0.01 vs. the Model groups

To verify the above speculation on the anti-COPD pharmacological mechanism of ISOF, firstly, the expression of 16 DEGs (Fig. 2D) involved in the PI3K/AKT pathway (KEGG database) was examined in lung tissue from rats with CS-induced COPD. ISOF treatment specifically reversed CS-induced alterations in *Pik3c3*, *Aqp4*, *Spp1, Lpar6, Ywhab, Bcl2l1, Col6a5, Faslg, Ghr, Gng11, Il2rg, Itgb6, Jak3, Lama2, Lpar1, Tp53* (Fig. 3A, B, C, and Additional file 1: Figure S5A–M). Activation of PI3K facilitates phosphorylation of AKT, and subsequent regulation of mTOR can promote T cell proliferation and even differentiation of Th17 cells [10, 28, 33]. Therefore, the effect of ISOF on AKT and mTOR was the subject of this study. ISOF was found that ISOF markedly counteracted the CS-induced elevated AKT phosphorylation, mTOR, and its phosphorylated expression in rat lung tissue, where there was no significant change for AKT expression, but significantly increased the ratio of AKT phosphorylation (Fig. 3D–E). And AKT phosphorylation and mTOR and its phosphorylation correlated positively with each other and with AQP4, SPP1, and PIK3C3 expression, but not with AKT (Fig. 3F, Additional file 1: Figure S6A–C). A positive correlation was also identified between AQP4 and SPP1 in 156 clinical cases (GSE76925-male, GSE130928-male, GSE130928-female and GSE112811) and PIK3C3 in 171 cases (GSE76925-female, GSE130928-male, GSE130928-female and GSE112811, Fig. 3G).

**Fig. 4:**
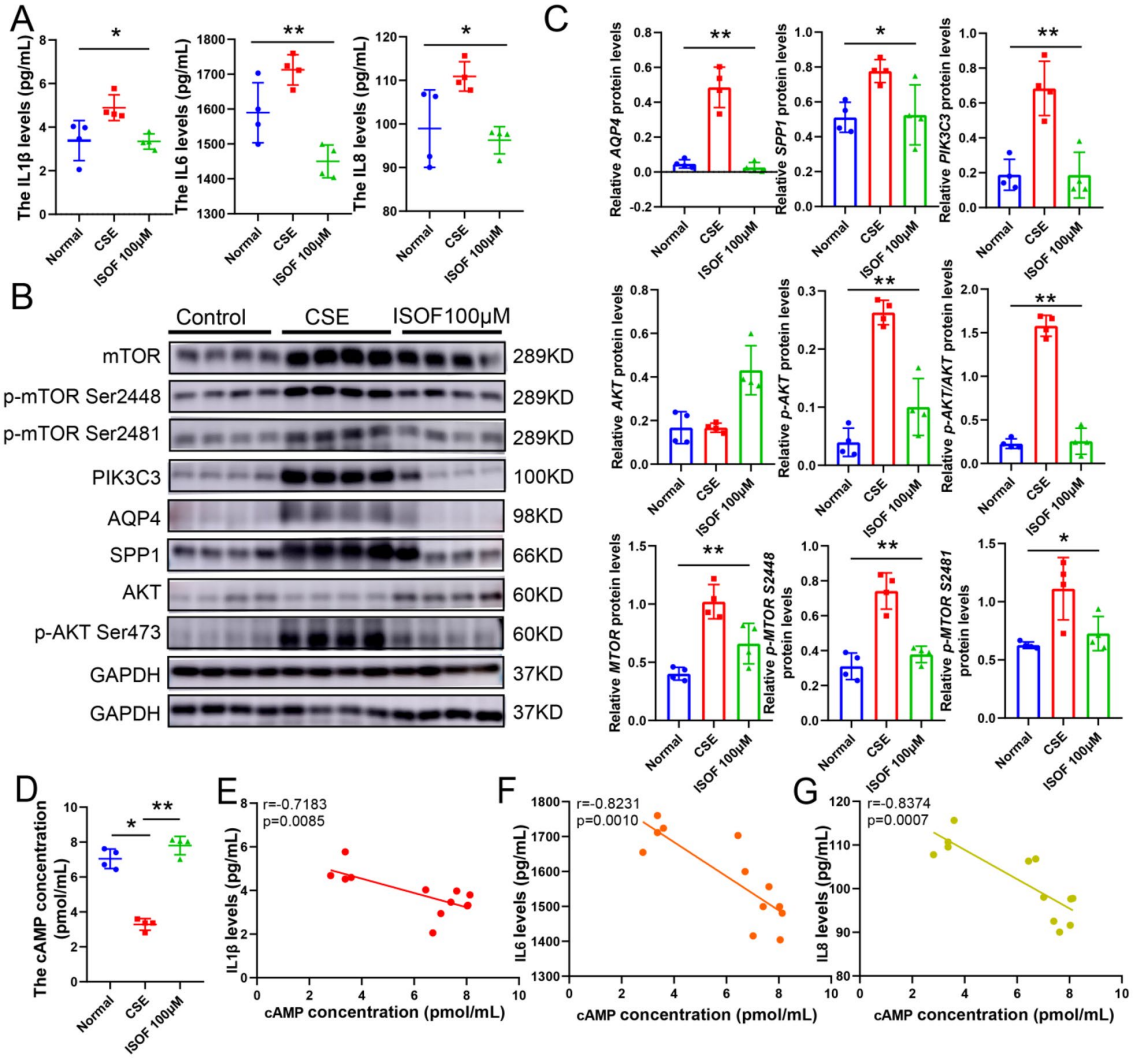
In vitro study of the anti-COPD pharmacological mechanism of ISOF. **A**: Detection of IL1β, IL6, and IL8 inflammatory factor protein levels; **B**: Western blot of PIK3C3, AQP4, SPP1, p-AKT, AKT, mTOR and p-mTOR (S2448 and S2481) protein expression, n = 4; **C**: Quantification of PIK3C3, AQP4, SPP1, p-AKT, AKT, mTOR and p-mTOR (S2448 and S2481) expression; **D**: Detection of cAMP protein levels; **E**: Correlation analysis of IL1B concentration on cAMP concentration, n = 12; **F**: Correlation analysis of IL6 on cAMP, n = 12; **G**: Correlation analysis of IL8 on cAMP, n = 12. GAPDH was homogenized as an internal reference, *P < 0.05, and **P < 0.01 vs. the CSE groups

Again, CSE stimulation was associated with increased levels of IL-1β, IL-6, and IL-8 (Fig. 4A), AQP4, SPP-1, and PIK3C3 with mTOR expression and phosphorylation levels of AKT and mTOR (Fig. 4B, C). The pro-inflammatory effect and the increased protein expression or phosphorylation of CSE almost disappeared when ISOF (100 μM, Additional file 1: Figure S7A–D) was used in combination with CSE. Also, ISOF elevated CSE leading to a decrease in cAMP (Fig. 4D). It was also found that IL-1β, IL-6, and IL-8 had significant positive correlations with cAMP, AQP4, SPP1, PIK3C3, AKT phosphorylation and mTOR, and its phosphorylation (Fig. 4E–G, and Additional file 1: Figure S8A–C), and cAMP, AQP4, SPP1, PIK3C3, AKT phosphorylation, and mTOR and its phosphorylation had strong correlations with each other (Additional file 1: Figure S9A–D). These implied that the anti-COPD effect of ISOF may be related to PI3K-AKT signaling pathway and targets such as AQP4, SPP1, PIK3C3, AKT, and mTOR as well as cAMP.

### Reduced PIK3C3 suppresses AKT-mTOR signaling activation

**Fig. 5:**
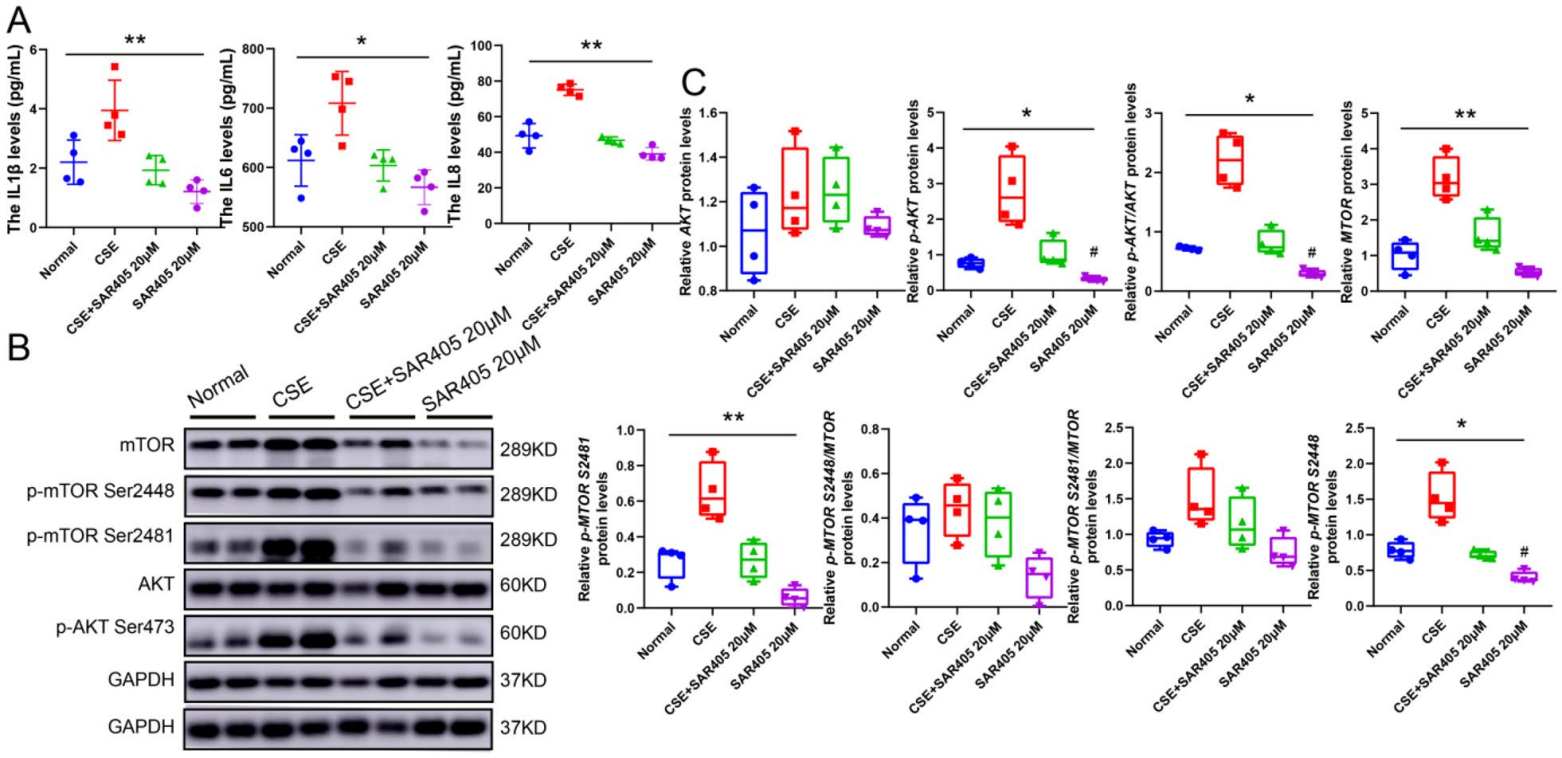
Diminished PIK3C3 represses the activation of AKT-mTOR signaling. **A**: Detection of IL1β, IL6, and IL8 protein levels; **B**: Western blot of AKT, p-AKT, mTOR and p-mTOR (S2448 and S2481) protein expression, n = 4; **C**: Quantification of AKT, p-AKT, mTOR and p-mTOR (S2448 and S2481) expression; GAPDH was homogenized as an internal reference, *P < 0.05, and **P < 0.01 vs. the CSE groups; #*P* < 0.05 vs. The Normal groups

To recognize the role of PIK3C3 in the inflammatory response, SAR405, a PIK3C3 inhibitor, was utilized in this study. SAR405 hindered the inflammatory effect (IL-1β, IL-6, and IL-8) triggered by CSE (Fig. 5A), and impeded the phosphorylation of AKT, and the expression and phosphorylation of mTOR (Fig. 5B–C). However, it didn't change the expression of AKT, suggesting that PIK3C3 can regulate the downstream expression of AKT and mTOR and then play an anti-inflammatory role in COPD.

### PIK3C3 signaling regulated by SPP1

**Fig. 6:**
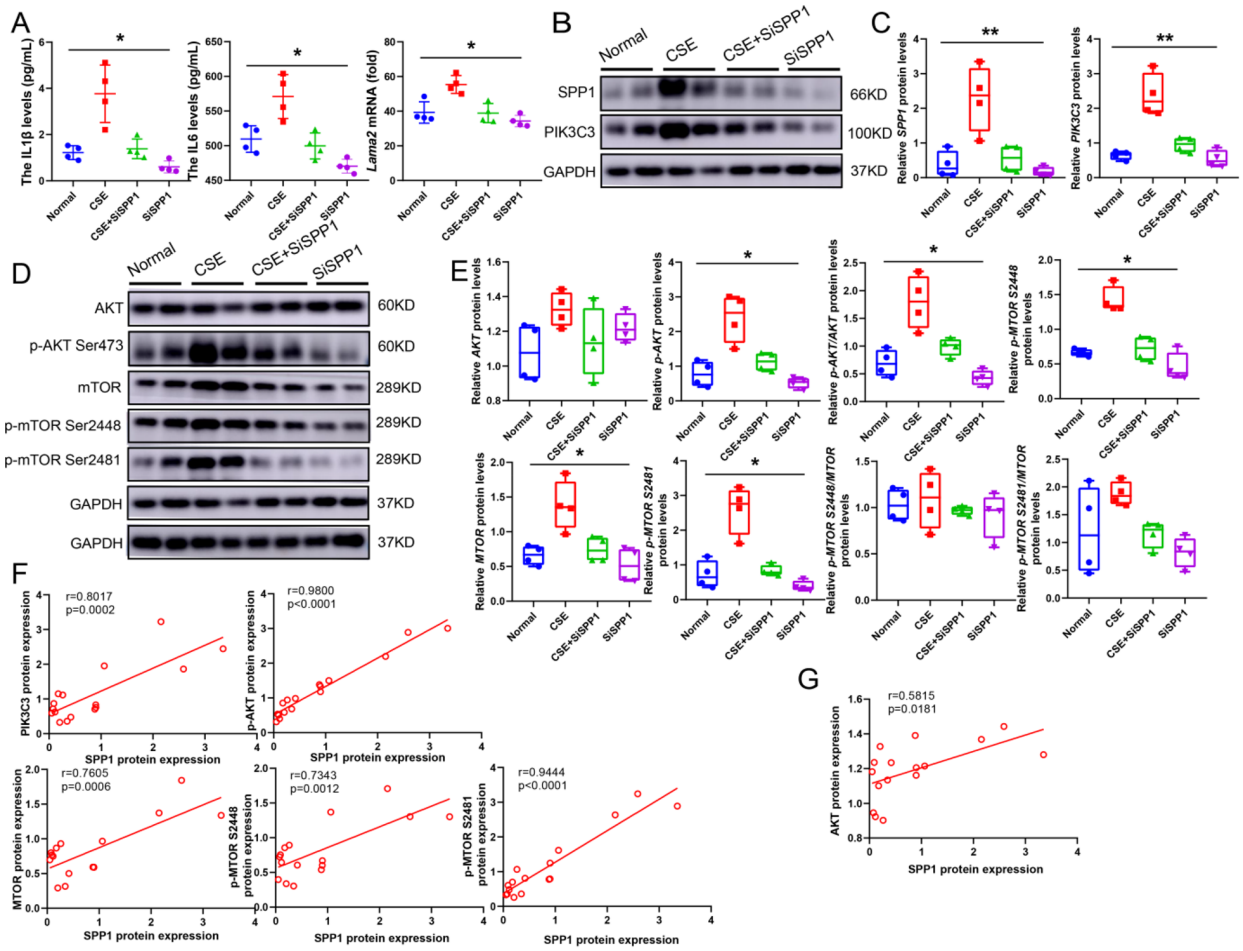
PIK3C3 signaling regulated by SPP1. **A**: Detection of IL1β, IL6, and IL8 protein levels; **B**: Protein expression of SPP1 and PIK3C3, n = 4; **C**: Quantification of PIK3C3 and SPP1 expression; **D**: Western blot of p-AKT, AKT, mTOR and p-mTOR (S2448 and S2481) protein expression; **E**: Quantification of p-AKT, AKT, MTOR and p-MTOR (S2448 and S2481) expression; **F**: Correlation analysis of SPP1 protein expression on PIK3C3, p-AKT, MTOR and p-MTOR protein expression, n = 16; **G**: Correlation analysis of SPP1 on AKT, n = 16; GAPDH was homogenized as an internal reference, *P < 0.05, and **P < 0.01 vs. the CSE groups

To understand the effects of SPP1 in COPD, an SPP1 knockdown group was established by siRNA3 (SPP1-homo-1491) and liposome 3000 (Additional file 1: Figure S9). Further study revealed that SPP1 knockdown reduced the secretion of IL-1β, IL-6, and IL-8 (Fig. 6A), and the expression or/and phosphorylation of SPP1, PIK3C3, AKT, and mTOR in the CSE group (Fig. 6B–C). Furthermore, AKT phosphorylation, MTOR expression and phosphorylation could be attenuated by SPP1 knockdown (Fig. 6D–E). Additionally, it was confirmed that variations in SPP1 expression had a distinct positive correlation with PIK3C3, AKT phosphorylation, MTOR and its phosphorylation (Fig. 6F), also a clear relationship with AKT (Fig. 6G), indicating that the anti-inflammatory effect mediated by SPP1 knockdown may be affected via reducing the expression of PIK3C3.

### Reduction of AQP4 activity abolished the CSE-induced inflammatory effect through inhibition of SPP1

**Fig. 7:**
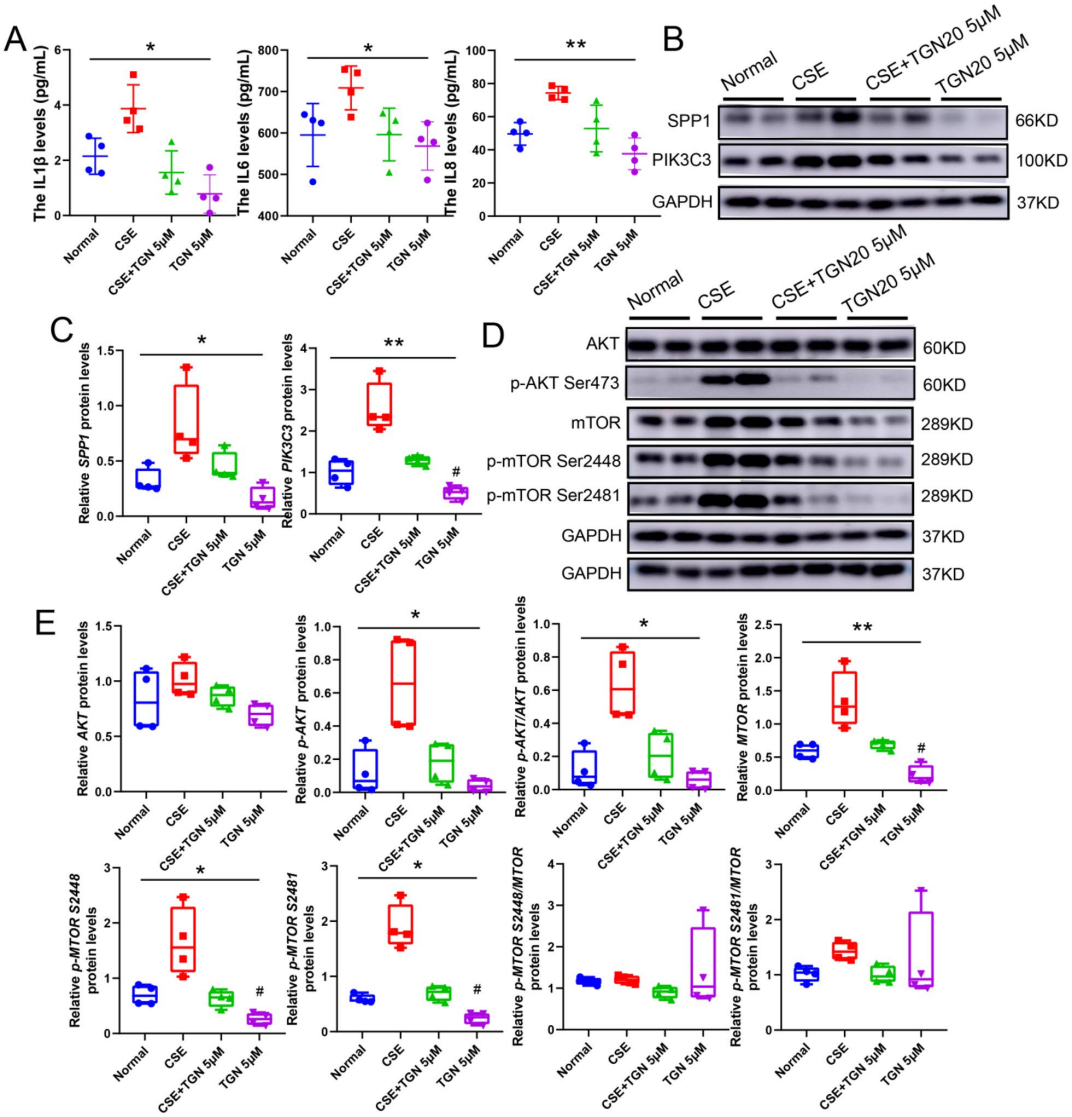
Reduction of AQP4 activity eliminated the CSE-induced inflammatory effect by inhibiting SPP1. **A**: Detection of IL1β, IL6, and IL8 protein levels; **B**: Protein expression of SPP1 and PIK3C3, n = 4; **C**: Quantification of PIK3C3 and SPP1 expression; **D**: Western blot of p-AKT, AKT, mTOR and p-mTOR (S2448 and S2481) protein expression; **E**: Quantification of p-AKT, AKT, MTOR and p-MTOR (S2448 and S2481) expression; GAPDH was homogenized as an internal reference, *P < 0.05, and **P < 0.01 vs. the CSE groups; #*P* < 0.05 vs. The Normal groups

To identify whether AQP4 serves an essential function in the anti-COPD inflammatory immune-related role of ISOF, treatment with TGN20 largely exempted CSE-induced increase in IL1 secretion (Fig. 7A) and elevated expression of SPP1 and PIK3C3 through AQP4 inactivation (Fig. 7B–C). Besides, TGN20 also attenuated AKT phosphorylation, MTOR expression and phosphorylation (Fig. 7D–E). These results connoted that AQP4 regulates SPP1, AKT, and mTOR, playing an anti-inflammatory role in COPD.

### The anti-COPD activity of ISOF was initiated by hindering AQP4 expression due to raising cAMP signaling

**Fig. 8:**
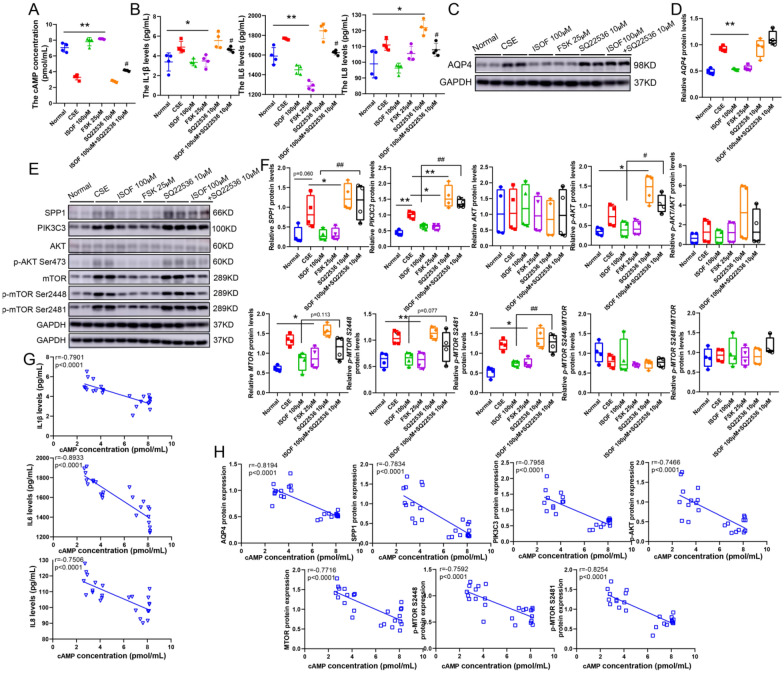
The anti-COPD activity of ISOF is initiated by hindering AQP4 expression due to raising cAMP signaling. **A**: Detection of cAMP levels; **B**: IL1β, IL6, and IL8 protein levels; **C**: Protein expression of AQP4, n = 4; **D**: Quantification of AQP4 expression; **E**: Western blot of SPP1, PIK3C3, p-AKT, AKT, mTOR and p-mTOR (S2448 and S2481) protein expression; **F**: Quantification of SPP1, PIK3C3, p-AKT, AKT, MTOR and p-MTOR (S2448 and S2481) expression; **G**: Correlation analysis of cAMP concentration on IL1β, IL6, and IL8 concentration, n = 24; **H**: Correlation analysis of cAMP concentration on AQP4, SPP1, PIK3C3, p-AKT, MTOR and p-MTOR protein expression, n = 24; GAPDH was homogenized as an internal reference, *P < 0.05, and **P < 0.01 vs. the CSE groups; #*P* < 0.05, and ##*P* < 0.01 vs. The ISOF 100 μM groups

cAMP can mediate the activation of the PI3K/AKT signaling pathway [11, 30]. FSK (ACs agonist) to activate cAMP and SQ22536 (ACs inhibitor) to decrease cAMP in BEAS2B cells were used to further confirm the causal relationship between increased cAMP, decreased AQP4 and anti-inflammation of ISOF. CSE decreased cAMP concentration and increased IL-1β, IL-6, and IL-8 levels, SQ22536 could potentiate it, while the addition of ISOF or FSK reversed the effect (Fig. 8A–B). And the increased expression of AQP4 by CSE could be partially repressed with ISOF or FSK (Fig. 8C–D), furthermore, SPP1, PIK3C3, AKT phosphorylation, and MTOR expression and phosphorylation were abrogated (Fig. 8E–F). But, in combination with SQ22536, it nearly disappeared (Fig. 8C–E). Similarly, movements in cAMP levels had a pronounced negative correlation with IL-1β, IL-6, IL-8, AQP4, SPP1, PIK3C3, AKT phosphorylation, MTOR expression and its phosphorylation (Fig. 8G–H), although not with AKT (Additional file 1: Figure S10), meaning that ISOF promotes intracellular cAMP concentration and then cAMP could regulate the expression and activity of downstream AQP4, SPP1, PIK3C3, AKT, and mTOR, thus exerting its anti-inflammatory effects.

## Discussions

The role of the development of inflammation in the pathological mechanisms of COPD has not been elucidated, although it is well-known that inflammation-mediated damage plays a key role in the pathogenesis of COPD [20]. Here, it was demonstrated that ISOF controlled the secretion of various inflammatory factors such as IL-1β, IL-6, and IL-8 by human airway epithelial cells via the cAMP pathway to minimize inflammation infiltration. Blocking the expression of AQP4, SPP1, and/or PIK3C3, and thus the activation of AKT-MTOR signaling may also achieve the anti-inflammatory effect of ISOF mediated by cAMP activation. Also, inactivation of AQP4 has a positive correlation with the inhibition of SPP1 and PIK3C3 expression, as evidenced by the clinical data. Finally, PIK3C3 has also been found to be partially regulated by SPP1 in respiratory diseases after altering the inflammatory environment via AKT-MTOR. How ISOF plays an anti-inflammatory role in COPD through cAMP signaling may therefore be well explained.

ISOF is the main active component of *Coleus forskohlii*, a plant native to Yunnan, China, a potent AC activator that induces an increase in intracellular cAMP [47]. cAMP has long been recognized as an inducer of anti-inflammatory responses, and cAMP-dependent pathways have been widely used in the pharmacological treatment of inflammatory diseases. Recently, cAMP has been indicated as a substance critical for the resolution of inflammation [44]. However, it is not yet fully understood how cAMP is involved in signaling to reach the critical anti-inflammatory pathway. cAMP has been reported to dose-control the expression of AQP4 in human astrocytes [44], SPP1 [4] in rat aortic and vascular smooth muscle cells, and PI3K in rat lung tissue and alveolar type II epithelial cells. AQP4 is a membrane-bound protein that plays a pro-inflammatory role by promoting the release of cytokines that activate microglia and other cells, among others [6, 35, 50]. Smoking increases the expression of SPP1 in induced sputum, and its levels are also increased in induced sputum [6, 35, 50] and lung tissue [1] from COPD patients (Miao and [47]. PI3K/AKT signaling pathway is implicated in many malignant, inflammatory, and autoimmune disease pathologies, including in COPD [23, 38, 41–44]. While PIK3C3 is a member of the PI3K family, PIK3C3 plays a key role in T cell metabolism and CD4 + T cell-mediated autoimmune diseases and can also regulate autophagy through the AKT-mTOR pathway [49]. Therefore, from the above results, ISOF caused an increase in intracellular cAMP levels to suppress AQP4 expression, consequently reducing SPP1 expression and blocking the inflammatory expansion process by preventing the activation of PI3K-AKT signaling via PIK3C3 to diminish the inflammatory infiltration of airway epithelial cells.

IL-1β, IL-6, and IL-8, typical pro-inflammatory cytokines, exert important roles in COPD. Clinical studies have implicated IL-1β, IL-6 and IL-8 in the pathogenesis of COPD [7, 14, 15]. Th17 cells are heterogeneous and their phenotypes can vary greatly depending on the cytokine milieu within which they differentiate, particularly IL-6 and IL-1β expression [24]. In vitro, IL-6 prove to be required for the differentiation of TCR-stimulated naive CD4 T cells into Th17 cells in mice, while IL-1β is involved in the expansion and maturation of Th17 cells [24]. IL8, also known as CXCL8, is a chemotactic cytokine known to be secreted by various cells responding to inflammatory stimuli and contributing to rapid infiltration of the respiratory system and propagation of inflammation in the early stages of disease [46]. These findings indicate that IL-1β, IL-6, and IL-8 are associated with the development and progression of COPD and, therefore, used as biomarkers of the model [7].

However, a limitation of the study was mainly the inability to thoroughly explore the role of ACs subtypes and COPD pathophysiology, as well as the biological functions of cAMP, AQP4, SPP1, and PIK3C3 at the in vivo level. And additional studies are needed to elucidate the network mechanisms behind the cAMP-AQP4-SPP1-PIK3C3 anti-inflammatory axis, T cell differentiation, and the pharmacological mechanisms associated with the anti-COPD of ISOF and an in-depth description of the ISOF mechanism for elevating cAMP.

**Fig. 9:**
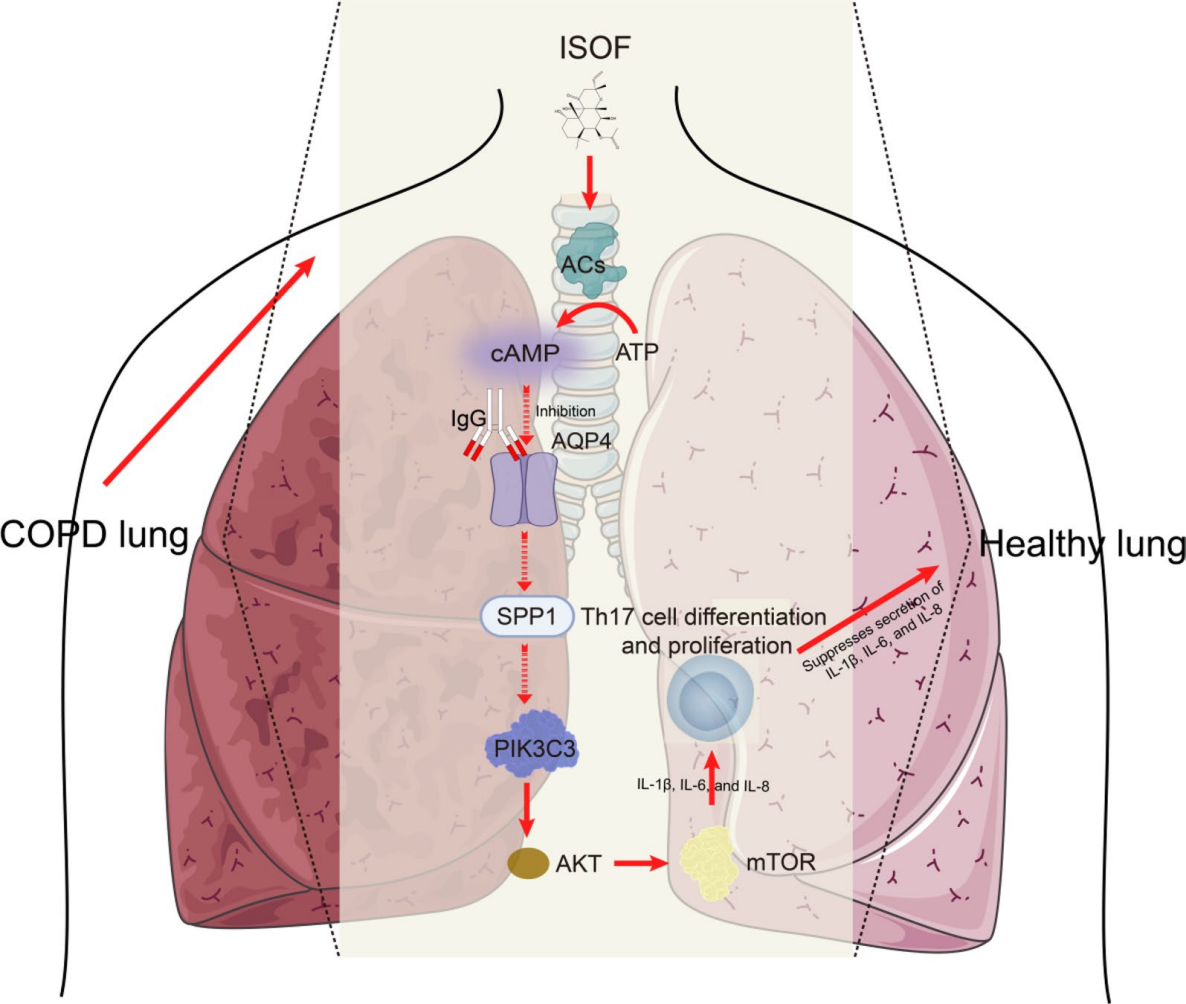
Diagram of the anti-COPD pharmacological mechanism of ISOF

In conclusion, this study confirms that the pharmacological mechanism of ISOF pertains to cAMP activation and impedes AQP4-SPP1-PIK3C3, diminishing the release of key downstream inflammatory factors IL-1β, IL-6, and IL-8 (Fig. 9). Meanwhile, there have been first reports of the anti-inflammatory effects of AQP4 and PIK3C3 in respiratory diseases. Thus, this may well broaden the content of how cAMP antagonizes COPD via PI3K-AKT-related signaling and also provide a theoretical basis for the treatment of COPD with ISOF. The results support the effects of ISOF via the cAMP/AQP4/SPP1/PIK3C3 axis on chronic airway inflammation, demonstrate its potential to reduce Th17 differentiation and thus the Th17/Treg balance, and provide potential targets for the treatment of this disease.

### Supplementary Information

**Additional file 1: Figure S1.** Volcano diagram of 8 arrays of differentially expressed genes included in the GEO database (Comparison criteria: gender and presence of smoking population). **Figure S2.** 61 common targets for ISOF and COPD. **Figure S3.** Network pharmacology and multi-omics studies. **Figure S4.** Basic information on blood transcriptomics. **A**: Principal component analysis in whole blood transcriptomic; **B**: DEGs volcano plot of Model vs Control; **C**: DEGs volcano plot of Model vs ISOF. **Figure S5.** Validation of mRNA expression levels of omics DEGs in rat lung tissues. **A**: *Bcl2l1*; **B**:* Col6a5*; **C**: *Faslg*; **D**:* Ghr*;* E*: *Gng11*;* F*: *Lama2*; **G**: *Lpar1*; **H**: *Lpar6*; **I**: *Il2rb*; **J**: *Itgb6*; **K**: *Jak3*; **L**: *Tp53*; **M**: *Ywhab*. **Figure S6.** Correlation analysis in vivo study. **Figure S7.** 100 μM as effective dose for ISOF in vitro studies. **A**: Detection of cAMP inflammatory factor protein levels; **B**: IL1β; **C**: IL6; **D**: IL8; n=2. **Figure S8. **Correlation analysis about IL-1β, IL-6, and IL-8 in vitro study. **Figure S9. **Correlation analysis about AQP4, SPP1, PIK3C3, AKT, and MTOR in vitro study. **Figure S10.** Screening for efficient SiRNA in knockdown SPP1 experiments (n=2). **Figure S11. **Correlation analysis of cAMP concentration (pmol/mL) on AQP4 protein expression in vitro cAMP regulation mechanism study (n=24). **Table S1.** The primers sequence for RT-PCR. **Table S2.** Basic information of ISOF-related targets in network pharmacology. **Table S3.** Basic information of COPD-related targets acquired through GEO. **Table S4.** Results of KEGG functional enrichment of common targets of ISOF and COPD in network pharmacology. **Table S5.** Basic information of the top 20 targets in the PPI network based on DEGs in ISOF vs M in three algorithms Degree, Betweenness Centrality and Closeness Centrality respectively (bold indicates the most important genes).

## Data Availability

DAVID database: https://david.ncifcrf.gov/tools.jsp; Draw Venn Diagram: http://bioinformatics.psb.ugent.be/webtools/Venn/; GEO database: https://www.ncbi.nlm.nih.gov/gds/; PharmMapper website: http://www.lilab-ecust.cn/pharmmapper/; PubChem database: https://pubchem.ncbi.nlm.nih.gov/; SEA Search Server: https://sea.bkslab.org/; STRING database: https://string-db.org/; SwissTargetPredict database: http://www.swisstargetprediction.ch/;

## Abbreviations

AC: Adenylate cyclase
cAMP: Cyclic adenosine monophosphate
COPD: Chronic obstructive pulmonary disease
CSE: Cigarette smoke extract
DEGs: Differentially expressed genes
DEPs: Differentially expressed proteins
FC: Fold chang
GO: Gene ontology
ISOF: Isoforskolin
KEGG: Kyoto Encyclopedia of Genes and Genomes
PI3K: Phosphoinositide 3 kinase
PPI: Protein–Protein interaction
WB: Western Blot
