# Supplementary material for: Isoforskolin modulates AQP4-SPP1-PIK3C3 related pathway for chronic obstructive pulmonary disease via cAMP signaling
Source: Chin Med. 2023 Oct 10;18:128. doi: 10.1186/s13020-023-00778-w (PMC10566078; doi:10.1186/s13020-023-00778-w)
Supplement: Supplementary file 1 — Additional file 1: Figure S1. Volcano diagram of 8 arrays of differentially expressed genes included in the GEO database (Comparison criteria: gender and presence of smoking population). Figure S2. 61 common targets for ISOF and COPD. Figure S3. Network pharmacology and multi-omics studies. Figure S4. Basic information on blood transcriptomics. A: Principal component analysis in whole blood transcriptomic; B: DEGs volcano plot of Model vs Control; C: DEGs volcano plot of Model vs ISOF. Figure S5. Validation of mRNA expression levels of omics DEGs in rat lung tissues. A: Bcl2l1; B: Col6a5; C: Faslg; D: Ghr; E: Gng11; F: Lama2; G: Lpar1; H: Lpar6; I: Il2rb; J: Itgb6; K: Jak3; L: Tp53; M: Ywhab. Figure S6. Correlation analysis in vivo study. Figure S7. 100 μM as effective dose for ISOF in vitro studies. A: Detection of cAMP inflammatory factor protein levels; B: IL1β; C: IL6; D: IL8; n=2. Figure S8. Correlation analysis about IL-1β, IL-6, and IL-8 in vitro study. Figure S9. Correlation analysis about AQP4, SPP1, PIK3C3, AKT, and MTOR in vitro study. Figure S10. Screening for efficient SiRNA in knockdown SPP1 experiments (n=2). Figure S11. Correlation analysis of cAMP concentration (pmol/mL) on AQP4 protein expression in vitro cAMP regulation mechanism study (n=24). Table S1. The primers sequence for RT-PCR. Table S2. Basic information of ISOF-related targets in network pharmacology. Table S3. Basic information of COPD-related targets acquired through GEO. Table S4. Results of KEGG functional enrichment of common targets of ISOF and COPD in network pharmacology. Table S5. Basic information of the top 20 targets in the PPI network based on DEGs in ISOF vs M in three algorithms Degree, Betweenness Centrality and Closeness Centrality respectively (bold indicates the most important genes). [file 13020_2023_778_MOESM1_ESM.docx]

**Additional file materials**

**Isoforskolin modulates AQP4-SPP1-PIK3C3 related pathway for chronic obstructive pulmonary disease via cAMP signaling**

Haochang Lin^1^, Sha Cheng^1,2,3^, Jiangya Li^1^, Xinyue Zhang^1^, Lueli Wang^1^, Liju Liang^1^, Qian Zhang^1^, Songye Yang^1^, Xiaoqian Zhou^1^, Furong Yang^1^, Jingfeng Song^1*^, Xue Cao^1,4*^, Weimin Yang^1*^, Zhiying Weng^1*^.

^1^ School of Pharmaceutical Science and Yunnan Key Laboratory of Pharmacology for Natural Products, Kunming Medical University, Kunming 650500, China

^2^ State Key Laboratory of Functions and Applications of Medicinal Plants, Guizhou Medical University, Guiyang 550014, Guizhou, China

^3^ Key Laboratory of Chemistry for Natural Products of Guizhou Province and Chinese Academy of Sciences, Guiyang 550014, Guizhou, China

^4^ Department of Laboratory Animal Science, Kunming Medical University, Kunming 650500, China

^*^Correspondence: Jingfeng Song ([462759891@qq.com; 0871-65922815),](mailto:462759891@qq.com,) Xue Cao ([dir1865@163.com; 0871-65922815),](mailto:dir1865@163.com,) Weimin Yang ([ywmbessie@yeah.net; 0871-65922815)](mailto:ywmbessie@yeah.net), and Zhiying Weng (weng_zy@sina.com; 0871-65922815).

Postal address: No. 1168, Chunrong West Road, Yuhua Street, Chenggong New Town, Kunming, China

**1. Additional file Figure**


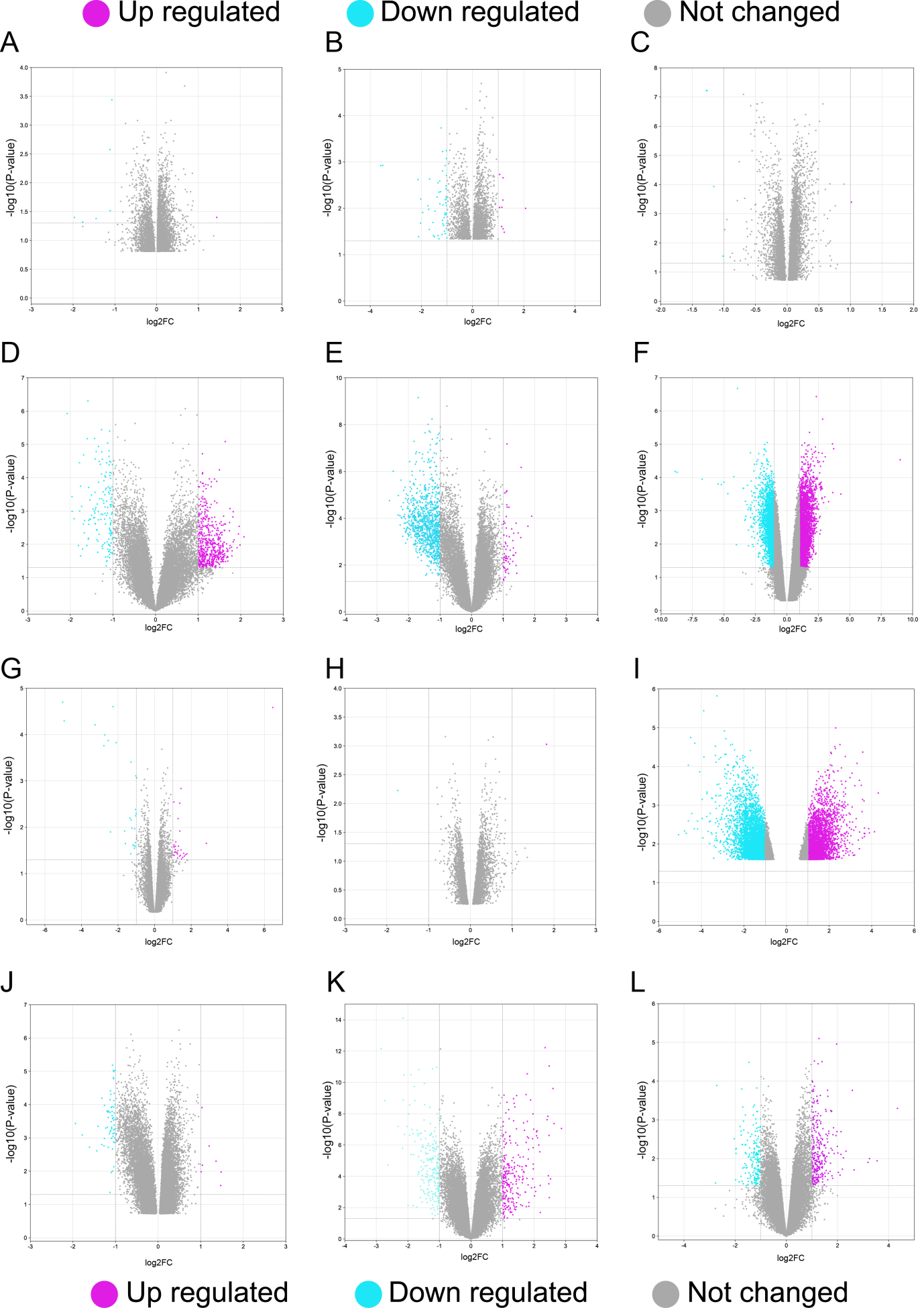


Figure S1. Volcano diagram of 8 arrays of differentially expressed genes included in the GEO database (Comparison criteria: gender and presence of smoking population). A): DEGs volcano plots of smoking normal subjects group vs. smoking COPD patients group in GSE37768; B): non-smoking normal subjects group vs. non-smoking COPD patients group in GSE37768; C): normal subjects group vs. COPD patient group in GSE73395; D): male normal subject group versus male COPD patient group in GSE76925; E): female normal subject group versus female COPD patient group in GSE76925; F): normal subject group versus COPD patient group in GSE106986; G): non-smoking normal subject group vs. non-smoking COPD patient group in GSE103174; H): smoking normal subject group vs. smoking COPD patient group in GSE103174; I): normal subject group vs. COPD patient group in GSE112260; J): normal subject group vs. COPD patient group in GSE112811; K): normal subjects group vs. COPD patients group in GSE130928 for males; L): normal subjects group vs. COPD patients group in GSE130928 for females.


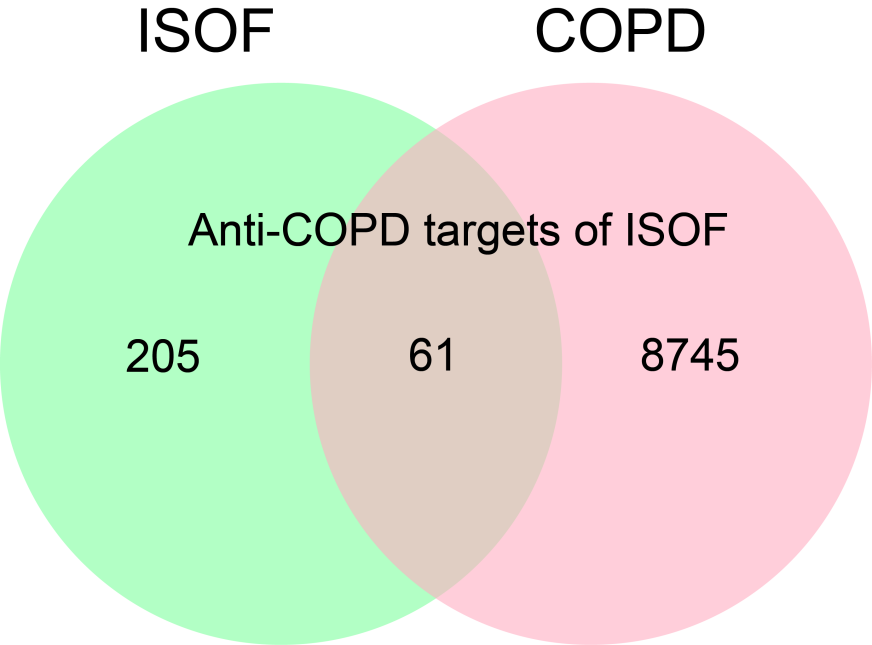


Figure S2. 61 common targets for ISOF and COPD


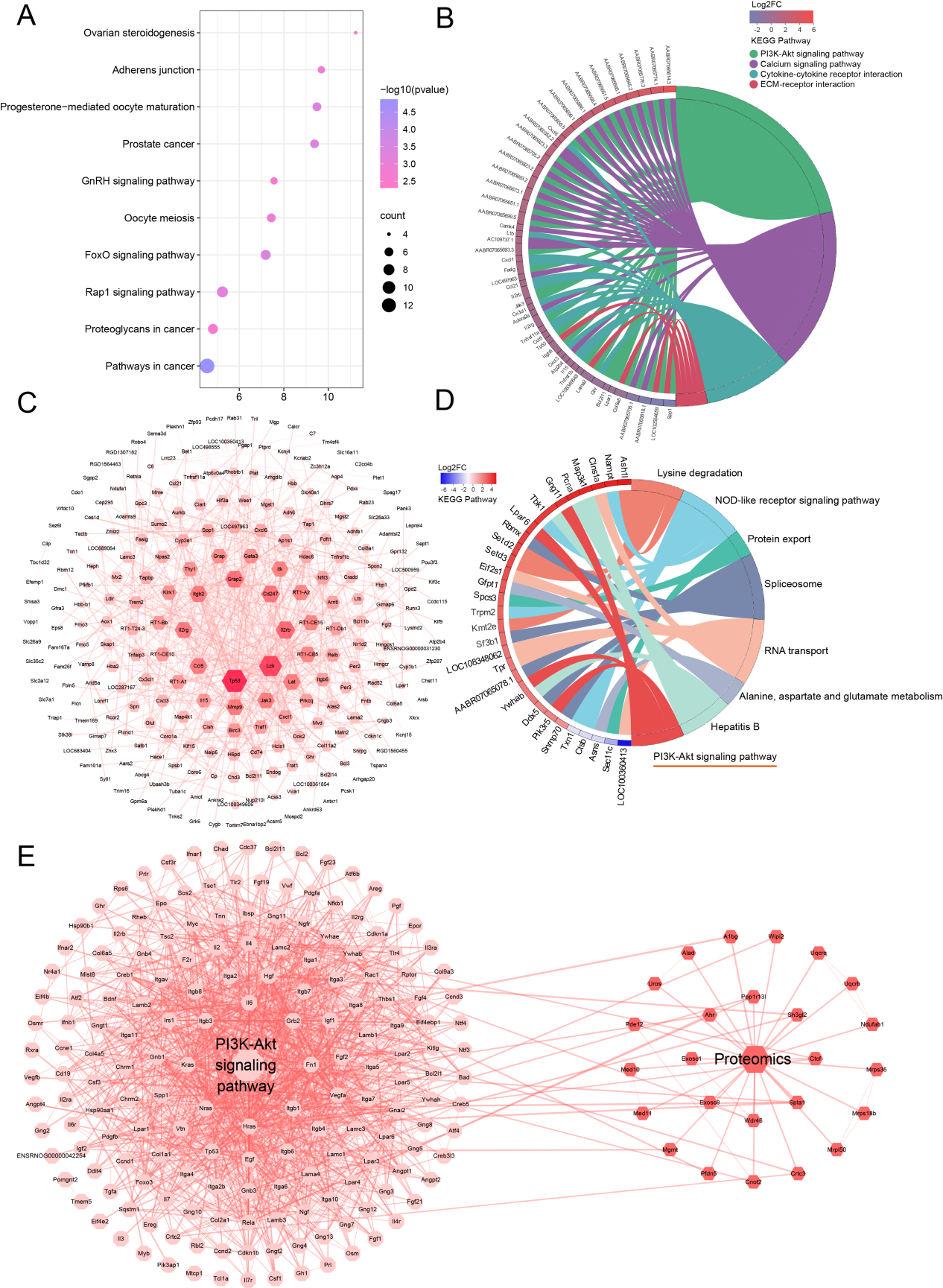


Figure S3. Network pharmacology and multi-omics studies. A): KEGG functional enrichment analysis of 61 common targets in ISOF vs COPD in network pharmacology; B): Enrichment chord plot of ISOF vs M based DEGs in transcriptomic analysis of lung tissues (only Pathway in cancer related pathways are shown); C): PPI analysis based on DEGs in ISOF vs M; D): Enrichment chord plot based on whole blood transcriptomic ISOF vs M DEGs KEGG functional enrichment analysis; E): PPI network of correlation between proteomics-related proteins and PI3K-Akt signaling pathway targets (pink positive octagonal nodes represent proteins of PI3K-Akt signaling pathway, red positive hexagonal nodes represent proteins group in the DEPs of ISOF vs M)


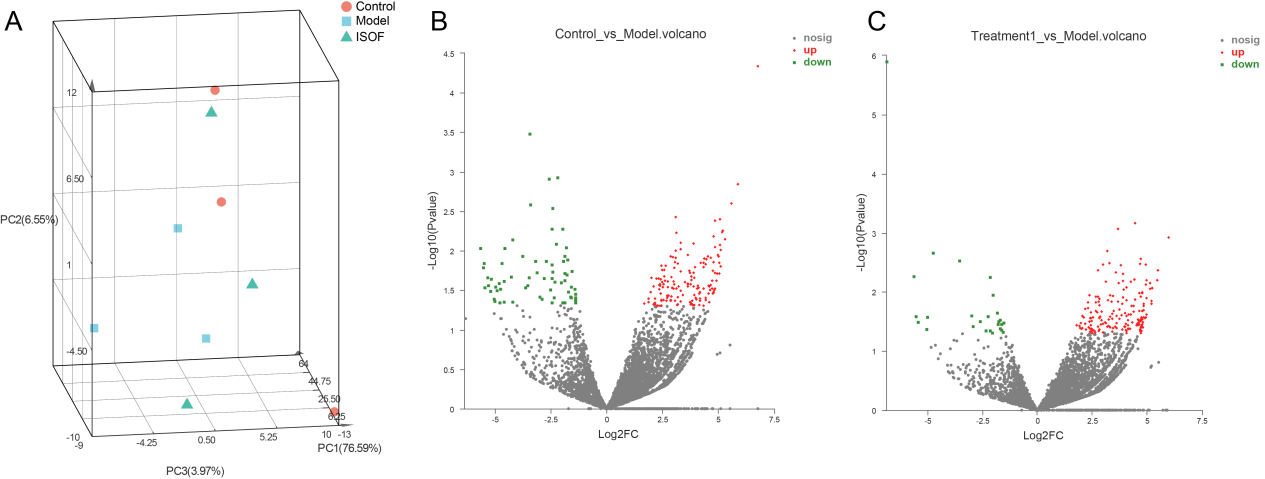


Figure S4. Basic information on blood transcriptomics. A): Principal component analysis in whole blood transcriptomic; B): DEGs volcano plot of Model vs Control; C): DEGs volcano plot of Model vs ISOF.


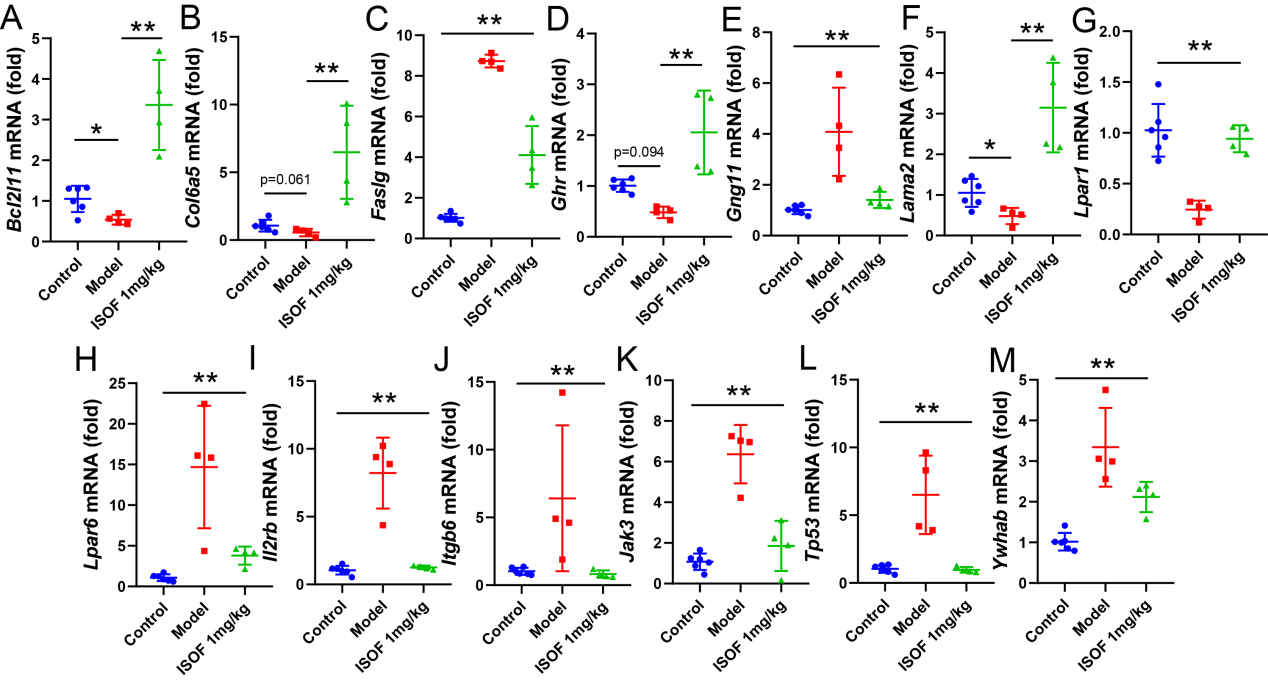


Figure S5. Validation of mRNA expression levels of omics DEGs in rat lung tissues. A): *Bcl2l1*; B): *Col6a5*; C): *Faslg*; D): *Ghr*; E): *Gng11*; F): *Lama2*; G): *Lpar1*; H): *Lpar6*; I): *Il2rb*; J): *Itgb6*; K): *Jak3*; L): *Tp53*; M): *Ywhab*.


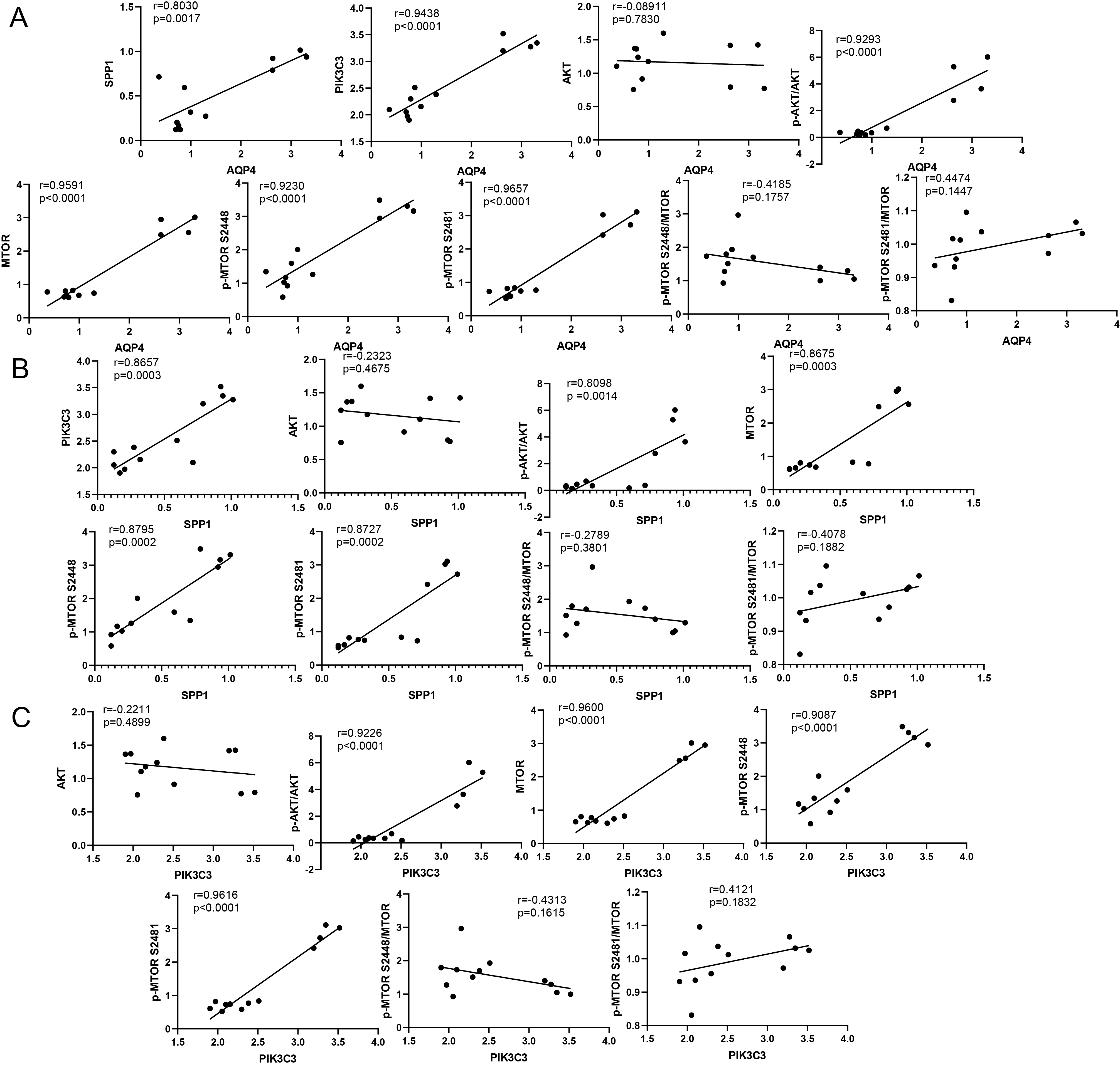


Figure S6. Correlation analysis in vivo study A): AQP4 protein expression on SPP1, PIK3C3, AKT, p-AKT/AKT, MTOR, p-MTOR and p-MTOR/MTOR protein expression; B): SPP1 on PIK3C3, AKT, p-AKT/AKT, MTOR, p-MTOR and p-MTOR/MTOR; C): PIK3C3 on AKT, p-AKT/AKT, MTOR, p-MTOR and p-MTOR/MTOR; n=12.


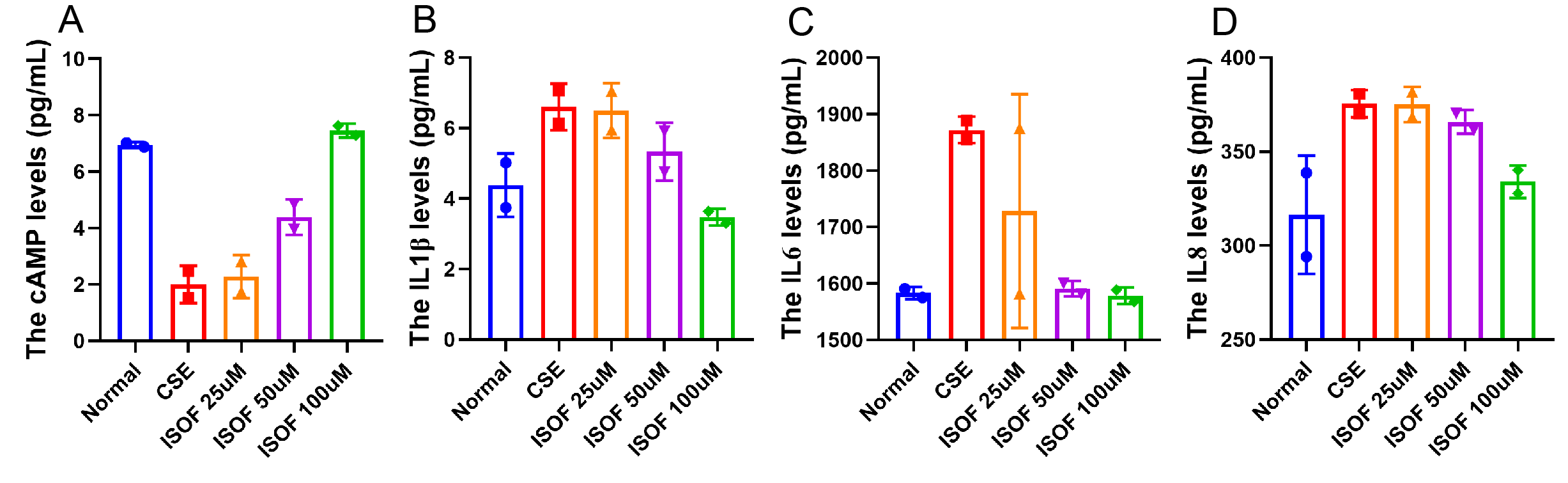


Figure S7. 100uM as effective dose for ISOF in vitro studies. A): Detection of cAMP inflammatory factor protein levels; B): IL1β; C): IL6; D): IL8; n=2.


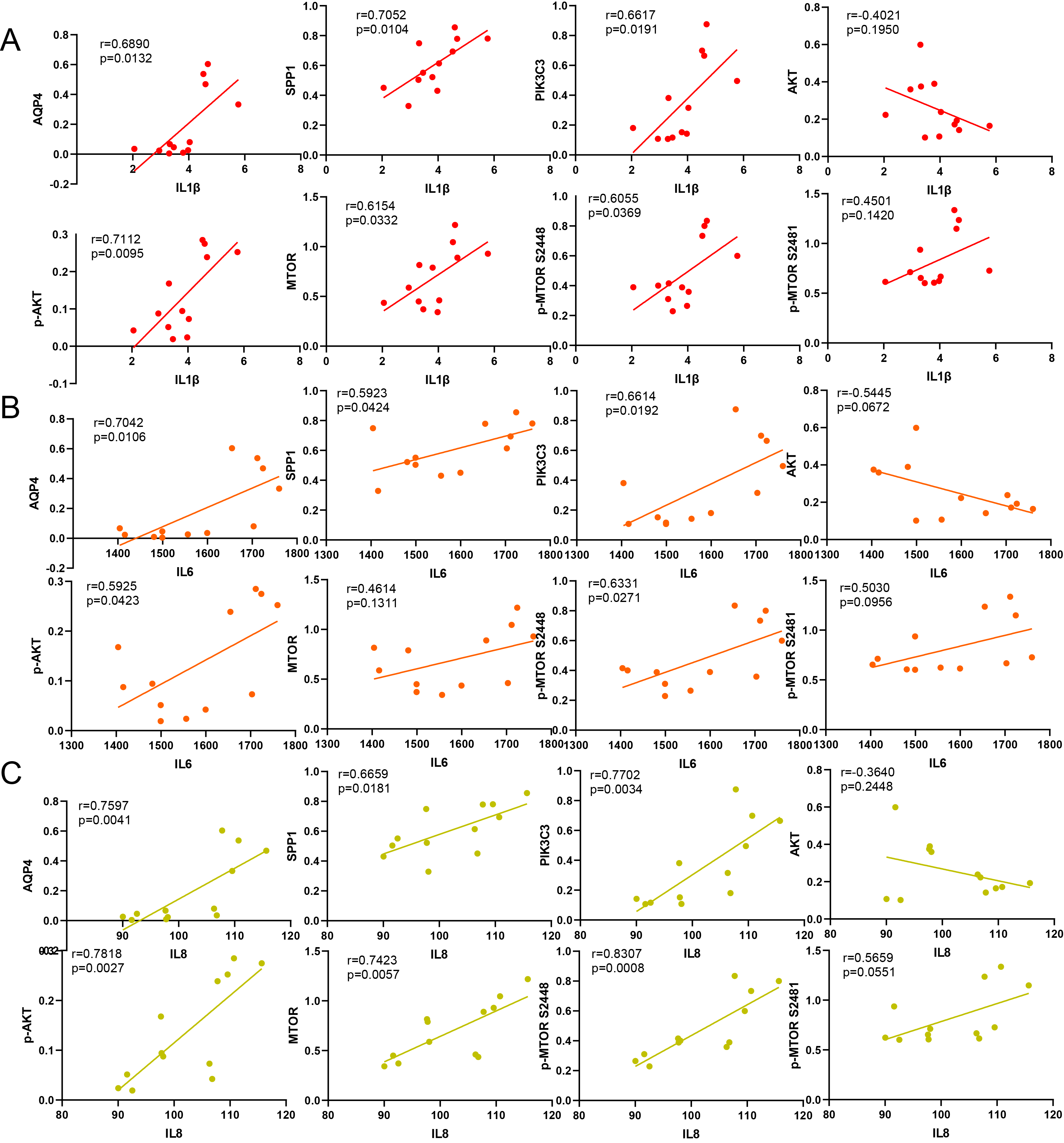


Figure S8. c A): IL1B concentration (pg/mL) on AQP4, SPP1, PIK3C3, AKT, p-AKT, MTOR, and p-MTOR protein expression; B): IL6 on AQP4, SPP1, PIK3C3, AKT, p-AKT, MTOR, and p-MTOR; C): IL8 on AQP4, SPP1, PIK3C3, AKT, p-AKT, MTOR, and p-MTOR; n=12.


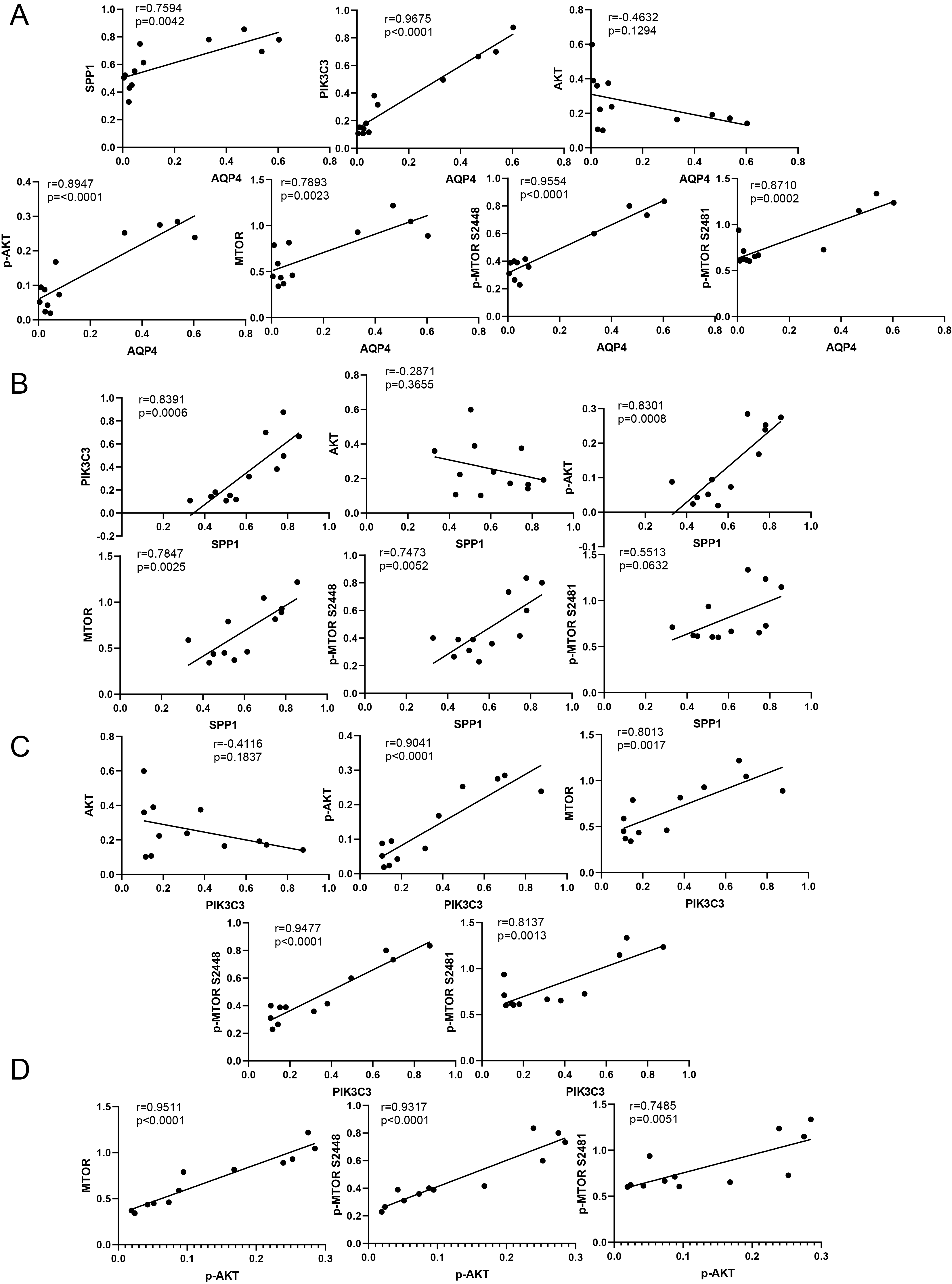


Figure S9. Correlation analysis about AQP4, SPP1, PIK3C3, AKT, and MTOR in vitro study A): AQP4 protein expression on SPP1, PIK3C3, AKT, p-AKT, MTOR, and p-MTOR protein expression; B): SPP1 on PIK3C3, AKT, p-AKT, MTOR, and p-MTOR; C): PIK3C3 on AKT, p-AKT, MTOR, and p-MTOR; D): p-AKT on MTOR, and p-MTOR; n=12.


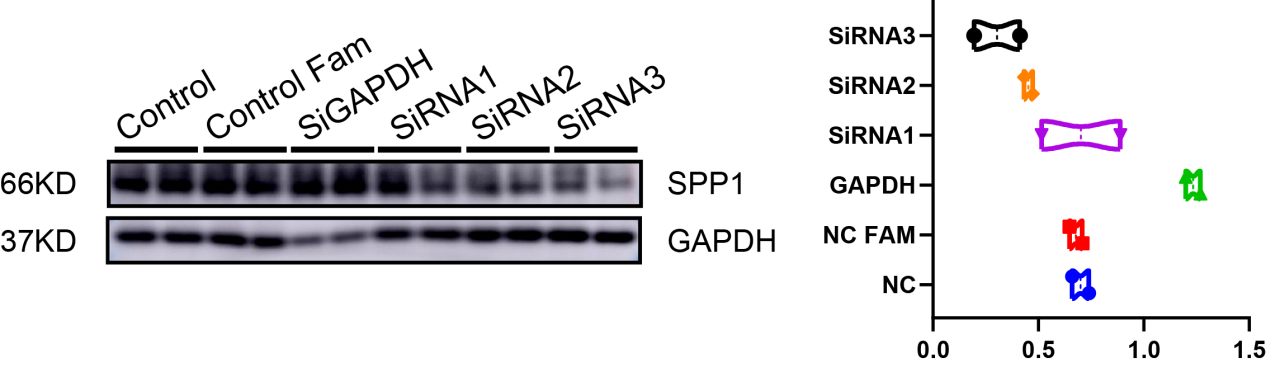


Figure S10. Screening for efficient SiRNA in knockdown SPP1 experiments (n=2).


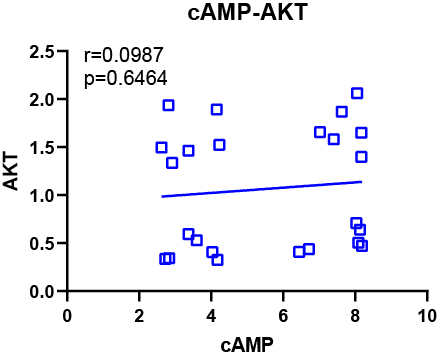


Figure S11. Correlation analysis of cAMP concentration (pmol/mL) on AQP4 protein expression in vitro cAMP regulation mechanism study (n=24)

1. **Additional file Table**

Table S1. The primers sequence for RT-PCR

| Gene symbol | Direction | Sequence |
| --- | --- | --- |
| *Lpar6* | Forward: | 5’-TCCCACTGCCCTTATGACGACTC-3’ |
|  | Reverse: | 5’-TATCAGCCCAAGCACAAACACCATG-3’ |
| *Ywhab* | Forward: | 5’-GTCAAACTCCCAGCAGGCGTAC-3’ |
|  | Reverse: | 5’-CAAGGCGAATCGGGTGTGTAGG-3’ |
| *Bcl2l1* | Forward: | 5’-GCGGATCGGAGACGAGTTCAATG-3’ |
|  | Reverse: | 5’-CCAGACCAGACGGAAGATGAATCG-3’ |
| *Col6a5* | Forward: | 5’-ACCTGTACCTGCTCTTCTCCTCTG-3’ |
|  | Reverse: | 5’-TCACCATCTTGCTTGCCGTTCG-3’ |
| *Ghr* | Forward: | 5’-GAGCCCGATATGGTCAACATCAGTC-3’ |
|  | Reverse: | 5’-GTCTGGATCTCACACGCACTTCG-3’ |
| *Il2rb* | Forward: | 5’-AGTTCTGAGCCTCAGCCGAC-3’ |
|  | Reverse: | 5’-TGGCTGCACTCCTGGAATGT-3’ |
| *Itgb6* | Forward: | 5’-GTGTCTGCGGCAAGTGTGTC-3’ |
|  | Reverse: | 5’-GATGCAGCTCCGTCTCGAGT-3’ |
| *Jak3* | Forward: | 5’-TGTAGCCTCTCATCCTCAGAAGCAG-3’ |
|  | Reverse: | 5’-ACACAGGTCCTCAGCCAAGTAGTC-3’ |
| *Lama2* | Forward: | 5’-CGCCCAGGAAATAAAGGACAGAAGG-3’ |
|  | Reverse: | 5’-CCATCAAGCACAGCAGACGAGTC-3’ |
| *Lpar1* | Forward: | 5’-GCTATGTTCGCCAGAGGACTATGAG-3’ |
|  | Reverse: | 5’-CAATAAAGGCACCCAGCACAATGAC-3’ |
| *Tp53* | Forward: | 5’-CGAGATGTTCCGAGAGCTGAATGAG-3’ |
|  | Reverse: | 5’-CTAGGCTGGAGGCTGGAGTGAG-3’ |
| *Pik3c3* | Forward: | 5’-AGGCAGATGGCTCCGAACCC-3’ |
|  | Reverse: | 5’-CTGTCCAACCAATCCACCTTCACC-3’ |
| *Spp1* | Forward: | 5’-GACGATGATGACGACGACGATGAC-3’ |
|  | Reverse: | 5’-GTGTGCTGGCAGTGAAGGACTC-3’ |
| *Aqp4* | Forward: | 5’-GAAGGCGGTCACAGCAGAGTTC-3’ |
|  | Reverse: | 5’-AGTCCAAAGCAGAGGGAGATGAGG-3’ |
| *Faslg* | Forward: | 5’-GCTGGTGGCTCTGGTTGGAATG-3’ |
|  | Reverse: | 5’-CTCTAAGGCTGTGGTTGGTGAACTC-3’ |
| *Gng11* | Forward: | 5’-CCGCCCTTCACATTGAGGATCTG -3’ |
|  | Reverse: | 5’-ACACCTGTTGTCTCTGCAACTTCAC-3’ |

Table S2. Basic information of ISOF-related targets in network pharmacology

| No. | Gene symbol | No. | Gene symbol | No. | Gene symbol | No. | Gene symbol | No. | Gene symbol | No. | Gene symbol |
| --- | --- | --- | --- | --- | --- | --- | --- | --- | --- | --- | --- |
| 1 | 10 | 46 | LYTA | 91 | FHUD | 136 | HSD17B10 | 181 | TYRS | 226 | TRI5 |
| 2 | 406102 | 47 | AVD | 92 | TTR | 137 | NUDC | 182 | CODA | 227 | PDPK1 |
| 3 | ADCY1 | 48 | SOP2 | 93 | CATA | 138 | PRX2 | 183 | EPHB4 | 228 | PTPN11 |
| 4 | GTR1 | 49 | APE1547.1 | 94 | PLA2G1B | 139 | ESR1 | 184 | ZIPA | 229 | LOX1.3 |
| 5 | NR0B1 | 50 | OBG | 95 | METE | 140 | BACE1 | 185 | AKR1C9 | 230 | AKR1C3 |
| 6 | RXFP1 | 51 | SSO2706 | 96 | GDI1 | 141 | GFRA1 | 186 | ART2B | 231 | PCM5 |
| 7 | OPD | 52 | INS | 97 | SRC | 142 | NOS2 | 187 | FABI | 232 | PCM6 |
| 8 | GAG-POL | 53 | OMPA | 98 | CIP1 | 143 | PDE4B | 188 | ADCY2 | 233 | PCM7 |
| 9 | KIF11 | 54 | PDE5A | 99 | CINA | 144 | ALPHA-LP | 189 | HSD17B1 | 234 | PCM8 |
| 10 | NSPA | 55 | FECA | 100 | PARP1 | 145 | BPHC | 190 | ETA | 235 | LAP3 |
| 11 | BMP2 | 56 | HEV1 | 101 | CDK2 | 146 | RPH | 191 | ALPHA-MAN-IIA | 236 | LACZ |
| 12 | PTGS1 | 57 | CA2 | 102 | GC | 147 | PF3D70907900 | 192 | KDSA | 237 | PDAD |
| 13 | NFT-1 | 58 | NR1H2 | 103 | NALP | 148 | AKR1B1 | 193 | CGT | 238 | YICI |
| 14 | STS | 59 | ACPS | 104 | CA1 | 149 | ACOX1 | 194 | CRK2 | 239 | FUCA |
| 15 | MUP1 | 60 | MAPK8 | 105 | CES1 | 150 | CALM1 | 195 | NA | 240 | CYP102A1 |
| 16 | OMPF | 61 | MMP3 | 106 | ATPE | 151 | CALM2-A | 196 | HAL3A | 241 | PTGS2 |
| 17 | KSI | 62 | CTSS | 107 | CYP2B4 | 152 | CALM2-B | 197 | THI1.2 | 242 | RBSD |
| 18 | RPLA | 63 | PRXC1A | 108 | LIP | 153 | SCGB1A1 | 198 | APX1 | 243 | TGFBR1 |
| 19 | MUP6 | 64 | UPRT | 109 | MAOB | 154 | CTXB | 199 | AXE1 | 244 | MDM2 |
| 20 | PLDA | 65 | PRKACA | 110 | AMPC | 155 | ROP | 200 | THIM | 245 | PGF |
| 21 | MAPK10 | 66 | CBL4 | 111 | ACADM | 156 | GRESAG | 201 | DHODH | 246 | CPA1 |
| 22 | KDR | 67 | LTP1 | 112 | MXIM | 157 | CLPP | 202 | CHI1 | 247 | RBSK |
| 23 | LET-70 | 68 | ITGAL | 113 | RADA | 158 | CEL | 203 | NPRS | 248 | MUTY |
| 24 | BOP | 69 | F2 | 114 | PPARD | 159 | CDK5R1 | 204 | TSX | 249 | ADK |
| 25 | E | 70 | HSD11B1 | 115 | CHEK1 | 160 | PGR | 205 | FGFR1 | 250 | C1R |
| 26 | CUT1 | 71 | TM1596 | 116 | NOS3 | 161 | XYLA | 206 | PPP5C | 251 | PTPN1 |
| 27 | DHLA | 72 | AMY1A | 117 | MAPK14 | 162 | HOM6 | 207 | SEC14L2 | 252 | PPARG |
| 28 | YHAI | 73 | AMY1B | 118 | METB | 163 | DUSP6 | 208 | OPMA | 253 | PHAA |
| 29 | BTUB | 74 | AMY1C | 119 | HNL | 164 | PPX | 209 | GSK3B | 254 | PDE4D |
| 30 | RHO | 75 | DRAA | 120 | PIK3CG | 165 | RORA | 210 | DEF | 255 | FUCI |
| 31 | PPIA | 76 | CEL7B | 121 | FOLB | 166 | MGG02252 | 211 | ACCC | 256 | PAM |
| 32 | MUP2 | 77 | QPCT | 122 | CCP1 | 167 | CFD | 212 | GLMS | 257 | ALOX15 |
| 33 | CASP7 | 78 | RNASE1 | 123 | ESTB | 168 | OMPT | 213 | GROS | 258 | ACK2 |
| 34 | MSDC | 79 | APOA2 | 124 | THYA | 169 | COMB | 214 | CTNNA1 | 259 | ADH1B |
| 35 | CYP19A1 | 80 | ANXA5 | 125 | FRR | 170 | DPS | 215 | NPR | 260 | POBA |
| 36 | GSTP1 | 81 | TAR | 126 | FABB | 171 | FHUA | 216 | NR3C2 | 261 | PEPP |
| 37 | GPD | 82 | TREM1 | 127 | AKR1C2 | 172 | CYP51 | 217 | ARGI | 262 | FBPC |
| 38 | CFB | 83 | FKBP1A | 128 | CPO | 173 | SULT2A1 | 218 | PLA2G10 | 263 | PDE10A |
| 39 | HEM2 | 84 | BCHE | 129 | VAOA | 174 | MUTB | 219 | IMPDH | 264 | CBR1 |
| 40 | PIM1 | 85 | CAMC | 130 | HSP90AA1 | 175 | DHA2112885 | 220 | LSRB | 265 | BLA |
| 41 | COBT | 86 | CCNA2 | 131 | AR | 176 | PNMT | 221 | AROD | 266 | TNNC1 |
| 42 | MAPK1 | 87 | NIRK | 132 | MIF | 177 | PYRG | 222 | SP0314 |  |  |
| 43 | ALB | 88 | FBPB | 133 | PH1313 | 178 | PAH | 223 | PLK1 |  |  |
| 44 | CAPNS1 | 89 | NAT | 134 | SDHA | 179 | FABP4 | 224 | WAS |  |  |
| 45 | FNTA | 90 | AROH | 135 | EGFR | 180 | DPP4 | 225 | NLPI |  |  |

Table S3. Basic information of COPD-related targets acquired through GEO

| No. | Gene symbol | No. | Gene symbol | No. | Gene symbol | No. | Gene symbol | No. | Gene symbol | No. | Gene symbol |
| --- | --- | --- | --- | --- | --- | --- | --- | --- | --- | --- | --- |
| 1 | GDF15 | 1469 | IQCC | 2937 | CUL9 | 4405 | MYO1D | 5873 | PSAT1 | 7341 | SCAI |
| 2 | SPATA18 | 1470 | ZNF761 | 2938 | LOC283038 | 4406 | KRT15 | 5874 | CLDN18 | 7342 | GGTA1P |
| 3 | GPR82 | 1471 | BNIP1 | 2939 | TRIM66 | 4407 | FRMPD3 | 5875 | MAP3K6 | 7343 | CHD7 |
| 4 | EREG | 1472 | ARHGAP5 | 2940 | LOC100272216 | 4408 | XRN1 | 5876 | RGR | 7344 | FBXO10 |
| 5 | CYP1B1 | 1473 | HCN2 | 2941 | PPP2R3B-AS1 | 4409 | SPPL2A | 5877 | HAND2-AS1 | 7345 | DGKQ |
| 6 | RPS4Y1 | 1474 | STK17B | 2942 | METTL20 | 4410 | RSRC2 | 5878 | MRPL30 | 7346 | MIR548AD |
| 7 | RPS4Y2 | 1475 | NPR1 | 2943 | LOC284009 | 4411 | GALNT9 | 5879 | COL4A5 | 7347 | PILRA |
| 8 | DDX3Y | 1476 | PRMT5 | 2944 | NRG1 | 4412 | PPFIBP1 | 5880 | NIPSNAP1 | 7348 | ZC2HC1B |
| 9 | USP9Y | 1477 | MEPE | 2945 | HOXA9 | 4413 | ANO6 | 5881 | PYCR1 | 7349 | ECHDC2 |
| 10 | NCRNA00185 | 1478 | AQR | 2946 | PHF8 | 4414 | NPC1L1 | 5882 | KIF25-AS1 | 7350 | FOLR1 |
| 11 | FGG | 1479 | H19 | 2947 | C1ORF145 | 4415 | FNIP2 | 5883 | NOTCH2 | 7351 | BACE1 |
| 12 | TXLNG2P | 1480 | MRPL20 | 2948 | ATP13A4 | 4416 | KAT5 | 5884 | MYRIP | 7352 | LINC00684 |
| 13 | KDM5D | 1481 | ANKLE2 | 2949 | LOC339593 | 4417 | PCSK6 | 5885 | RP11-68L1.2 | 7353 | FBXO44 |
| 14 | GSTT1 | 1482 | MCF2L | 2950 | CATSPER2 | 4418 | DNMT1 | 5886 | POM121L2 | 7354 | OSGEPL1 |
| 15 | SERPINE1 | 1483 | ZYX | 2951 | FAM47A | 4419 | SERAC1 | 5887 | SEMA5B | 7355 | SLC30A3 |
| 16 | XAGE1A | 1484 | RBM38 | 2952 | DGKH | 4420 | ITGA5 | 5888 | ELFN1 | 7356 | ARHGEF40 |
| 17 | GYG2 | 1485 | CCDC109B | 2953 | FLJ42392 | 4421 | SGTB | 5889 | HIST3H3 | 7357 | KCNAB1 |
| 18 | ZFY | 1486 | KCND2 | 2954 | UBAP2 | 4422 | RNF24 | 5890 | DLST | 7358 | RAVER1 |
| 19 | HBEGF | 1487 | ADAMTSL4 | 2955 | DEFB132 | 4423 | IL2RA | 5891 | C16ORF45 | 7359 | ZEB1 |
| 20 | SERPIND1 | 1488 | SCG5 | 2956 | ATP8 | 4424 | TNNT2 | 5892 | TTC40 | 7360 | CCDC37 |
| 21 | EIF1AY | 1489 | MAML1 | 2957 | LOC100190939 | 4425 | HGF | 5893 | ITGAM | 7361 | SAG |
| 22 | TMEM163 | 1490 | CBLC | 2958 | OR52A1 | 4426 | STAP2 | 5894 | CEP97 | 7362 | DCAF4 |
| 23 | UTY | 1491 | DOLK | 2959 | EMX2OS | 4427 | ACOX3 | 5895 | ATAD3A | 7363 | ZFP69 |
| 24 | IL6 | 1492 | DDX51 | 2960 | THOC1 | 4428 | ZFYVE26 | 5896 | EBNA1BP2 | 7364 | BTF3 |
| 25 | AGTR2 | 1493 | GCH1 | 2961 | ALDH3B2 | 4429 | CLEC10A | 5897 | BOP1 | 7365 | HEPH |
| 26 | AEN | 1494 | MLPH | 2962 | GPR75 | 4430 | FGFR1 | 5898 | SMC5 | 7366 | ATP8B3 |
| 27 | C20ORF3 | 1495 | CCDC115 | 2963 | LOC100128851 | 4431 | CHD3 | 5899 | LINC00152 | 7367 | CYP4B1 |
| 28 | TTTY15 | 1496 | NUPL1 | 2964 | HOXD3 | 4432 | EID3 | 5900 | LOC100996679 | 7368 | ANO2 |
| 29 | SEC23B | 1497 | TP53 | 2965 | ARID1B | 4433 | TANK | 5901 | CP | 7369 | ASL |
| 30 | CHIT1 | 1498 | FANCD2 | 2966 | KLHL14 | 4434 | PPP2R5C | 5902 | CACNA1G | 7370 | F11 |
| 31 | FGFBP1 | 1499 | TNFSF4 | 2967 | NDST3 | 4435 | CD38 | 5903 | NDUFB2 | 7371 | ZYG11B |
| 32 | IMPA2 | 1500 | RPL13A | 2968 | H2AFJ | 4436 | SLC22A9 | 5904 | RP11-815J21.4 | 7372 | FBXO4 |
| 33 | LINC00230A | 1501 | REPS2 | 2969 | WDR6 | 4437 | FAM160B2 | 5905 | LINC00930 | 7373 | AOC3 |
| 34 | CXCL3 | 1502 | TPO | 2970 | LOC100131581 | 4438 | C16ORF71 | 5906 | MIEF2 | 7374 | LHCGR |
| 35 | LOC96610 | 1503 | NFKB2 | 2971 | DKFZP686M1136 | 4439 | SEMA3A | 5907 | CSMD3 | 7375 | KRBA1 |
| 36 | TMEM158 | 1504 | TYR | 2972 | TBC1D8B | 4440 | PHC1 | 5908 | TRBV21OR9-2 | 7376 | BDNF |
| 37 | GMPPB | 1505 | CAV2 | 2973 | UTRN | 4441 | CEMIP | 5909 | TRMT44 | 7377 | LZTR1 |
| 38 | PER3 | 1506 | ARID2 | 2974 | MLL2 | 4442 | PDE9A | 5910 | C3ORF38 | 7378 | SLC5A7 |
| 39 | TNFAIP6 | 1507 | BCKDHB | 2975 | LOC100130547 | 4443 | CDH5 | 5911 | HOOK2 | 7379 | DNAH5 |
| 40 | DKK1 | 1508 | POTEM | 2976 | KRT26 | 4444 | IRAK1 | 5912 | CYB5R2 | 7380 | RP4-565E6.1 |
| 41 | IER3 | 1509 | TBCCD1 | 2977 | FBXL20 | 4445 | CD48 | 5913 | RP6-99M1.2 | 7381 | BCAR1 |
| 42 | LRRC58 | 1510 | LYZL2 | 2978 | ZFYVE9 | 4446 | MCM7 | 5914 | SLC25A15 | 7382 | HRK |
| 43 | CLDN2 | 1511 | ZNF672 | 2979 | LOC100129186 | 4447 | C1ORF112 | 5915 | ANKRD33 | 7383 | PPAP2A |
| 44 | UBE2E3 | 1512 | ZMYND19 | 2980 | AMIGO1 | 4448 | SRP68 | 5916 | IGSF1 | 7384 | DCDC2B |
| 45 | KRT16P2 | 1513 | NNMT | 2981 | KIAA0317 | 4449 | COG2 | 5917 | GUCY1B3 | 7385 | WDR49 |
| 46 | AHCY | 1514 | LY6E | 2982 | FGF1 | 4450 | ENOSF1 | 5918 | IGF2R | 7386 | RIMKLA |
| 47 | TMEM22 | 1515 | BHLHE23 | 2983 | LOC100131096 | 4451 | ATP2A1 | 5919 | ERICH3 | 7387 | LARGE |
| 48 | SUCLA2 | 1516 | DMRTA1 | 2984 | PHF12 | 4452 | LMCD1 | 5920 | VEPH1 | 7388 | APOB |
| 49 | TREM2 | 1517 | XPO5 | 2985 | EDEM3 | 4453 | ARHGAP22 | 5921 | HSD3B7 | 7389 | DDIAS |
| 50 | AREG | 1518 | KCNF1 | 2986 | CDKL4 | 4454 | TBC1D7 | 5922 | C11ORF84 | 7390 | COA1 |
| 51 | TM4SF1 | 1519 | F3 | 2987 | ANKRD17 | 4455 | DNAH7 | 5923 | LGALS3 | 7391 | AF121898.3 |
| 52 | FGA | 1520 | MAD1L1 | 2988 | KLHL31 | 4456 | SCN4A | 5924 | LAMA3 | 7392 | COL4A1 |
| 53 | PLA2G2A | 1521 | VWC2 | 2989 | HHIPL1 | 4457 | ABHD16A | 5925 | PRKD2 | 7393 | LRRK2 |
| 54 | ALDH3A1 | 1522 | RWDD4 | 2990 | OR4C15 | 4458 | RP11-513G19.1 | 5926 | AQPEP | 7394 | MPV17 |
| 55 | CXCL5 | 1523 | PSMA2 | 2991 | ANKRD20A1 | 4459 | C16ORF62 | 5927 | DNAH8 | 7395 | FHAD1 |
| 56 | CPZ | 1524 | APOD | 2992 | GCFC1 | 4460 | MROH8 | 5928 | KIAA0586 | 7396 | NDUFA2 |
| 57 | CCL8 | 1525 | C6ORF89 | 2993 | CTNND1 | 4461 | FAM20C | 5929 | A2ML1 | 7397 | MCM3AP |
| 58 | TMEM203 | 1526 | ZBTB11 | 2994 | HIST1H1D | 4462 | MYBPC3 | 5930 | KIAA0368 | 7398 | RPS6KA4 |
| 59 | HS3ST2 | 1527 | TMEM115 | 2995 | PLCXD2 | 4463 | APBB3 | 5931 | DPP9 | 7399 | RP13-143G15.4 |
| 60 | SHKBP1 | 1528 | THOC3 | 2996 | GNG13 | 4464 | ANKRD27 | 5932 | BMS1P20 | 7400 | USH1C |
| 61 | KBTBD4 | 1529 | RNF145 | 2997 | DMXL2 | 4465 | SYBU | 5933 | LOC101060275 | 7401 | ETNK2 |
| 62 | NECAP1 | 1530 | HES6 | 2998 | HNRNPK | 4466 | DIS3 | 5934 | CBLB | 7402 | CCDC122 |
| 63 | ITM2C | 1531 | ESAM | 2999 | OR5AP2 | 4467 | LOC93432 | 5935 | CYP4X1 | 7403 | STXBP4 |
| 64 | CCL20 | 1532 | AHCTF1 | 3000 | MRPS16 | 4468 | NLRC5 | 5936 | SOX2-OT | 7404 | VWA3A |
| 65 | EPHA1 | 1533 | SEC16A | 3001 | FSCB | 4469 | PDLIM5 | 5937 | COL6A4P1 | 7405 | MTRR |
| 66 | CYR61 | 1534 | CHMP2B | 3002 | TACR2 | 4470 | ASGR2 | 5938 | NUP205 | 7406 | IGFBP1 |
| 67 | CSF3 | 1535 | FKBPL | 3003 | KRTAP10-12 | 4471 | WDR91 | 5939 | ZNF250 | 7407 | TBX2 |
| 68 | CLP1 | 1536 | AP4M1 | 3004 | CD5L | 4472 | USP53 | 5940 | FAAH | 7408 | LRRCC1 |
| 69 | DGAT2 | 1537 | ACPP | 3005 | NKG7 | 4473 | ABCC4 | 5941 | UBASH3A | 7409 | DNAJC4 |
| 70 | LMNA | 1538 | PGGT1B | 3006 | MICAL3 | 4474 | C20ORF194 | 5942 | HECTD4 | 7410 | BMPR1A |
| 71 | MRC1 | 1539 | BPNT1 | 3007 | PLAC1 | 4475 | SPTBN5 | 5943 | PSPN | 7411 | ACBD4 |
| 72 | KRT14 | 1540 | PIK3R4 | 3008 | GOLGA8E | 4476 | NLRP3 | 5944 | TPPP3 | 7412 | PEX3 |
| 73 | NAPSA | 1541 | PHF23 | 3009 | RBPJL | 4477 | CENPF | 5945 | HDAC7 | 7413 | C12ORF42 |
| 74 | PLEKHB2 | 1542 | ACTA2 | 3010 | SYNGAP1 | 4478 | NRCAM | 5946 | SCNN1D | 7414 | EPB41L1 |
| 75 | KRT5 | 1543 | NIPSNAP3A | 3011 | SNAR-A3 | 4479 | CALU | 5947 | GSTA2 | 7415 | DDX31 |
| 76 | GALNT11 | 1544 | C7ORF58 | 3012 | LOC100130876 | 4480 | TMEM253 | 5948 | WDR26 | 7416 | COG1 |
| 77 | PRKAB1 | 1545 | PVRL1 | 3013 | LOC339807 | 4481 | MYH4 | 5949 | FENDRR | 7417 | RBP4 |
| 78 | RRAD | 1546 | FAM160B1 | 3014 | UBN2 | 4482 | COL22A1 | 5950 | FICD | 7418 | METTL7A |
| 79 | GOLM1 | 1547 | SLC15A4 | 3015 | UBAP1L | 4483 | SEMA5A | 5951 | OTUD3 | 7419 | PAAF1 |
| 80 | IST1 | 1548 | GMFB | 3016 | LAMA4 | 4484 | ALOX15 | 5952 | SH3GL1 | 7420 | RNF217 |
| 81 | SLC2A8 | 1549 | CAMSAP2 | 3017 | ANKRD36 | 4485 | KCTD9 | 5953 | TRIM13 | 7421 | TDRD1 |
| 82 | ABHD2 | 1550 | YIPF6 | 3018 | TTC28 | 4486 | WRAP53 | 5954 | CDK18 | 7422 | ENPP5 |
| 83 | PFKFB2 | 1551 | PSMD6 | 3019 | PGM5P2 | 4487 | CACNA1I | 5955 | GIMAP1-GIMAP5 | 7423 | TBCK |
| 84 | DNPEP | 1552 | IFNGR2 | 3020 | RABL2B | 4488 | VAV3 | 5956 | SCN10A | 7424 | GAD2 |
| 85 | PTPRO | 1553 | PPAPDC1B | 3021 | ZNF90 | 4489 | DOCK7 | 5957 | APOA1 | 7425 | RND3 |
| 86 | LOC100653210 | 1554 | CDK2AP1 | 3022 | PPM1K | 4490 | EHD2 | 5958 | ALS2CR11 | 7426 | LINC00277 |
| 87 | TDO2 | 1555 | DNAJA3 | 3023 | LOC284108 | 4491 | ESPNL | 5959 | ABR | 7427 | LINC00869 |
| 88 | NLGN4Y | 1556 | CD40 | 3024 | WDR87 | 4492 | RHOJ | 5960 | PC | 7428 | MT-TT |
| 89 | PNO1 | 1557 | NRIP1 | 3025 | DEFA5 | 4493 | TRPM2 | 5961 | RNF212B | 7429 | CTSB |
| 90 | UPP1 | 1558 | SNRPN | 3026 | PTCH2 | 4494 | MAN2A2 | 5962 | PNLIPRP1 | 7430 | URB1 |
| 91 | HPR | 1559 | RBM3 | 3027 | CCDC147 | 4495 | CAPRIN1 | 5963 | SQLE | 7431 | RYR3 |
| 92 | PDE6D | 1560 | PPID | 3028 | SYNPO2L | 4496 | TM4SF19-TCTEX1D2 | 5964 | CATSPER4 | 7432 | FGD5 |
| 93 | PLAUR | 1561 | ZNF622 | 3029 | ERV18-1 | 4497 | GAB2 | 5965 | FPGT | 7433 | UACA |
| 94 | ATF3 | 1562 | ZNF211 | 3030 | BRD1 | 4498 | GPC4 | 5966 | ARHGAP22-IT1 | 7434 | ACSS1 |
| 95 | UBE2N | 1563 | SEC24D | 3031 | LOC100131372 | 4499 | KIF5C | 5967 | CUL1 | 7435 | CTSC |
| 96 | AQP3 | 1564 | CHMP4C | 3032 | FAM168A | 4500 | PHLDB1 | 5968 | KCTD7 | 7436 | CDC45 |
| 97 | GYG2P1 | 1565 | PSMB2 | 3033 | SLC6A2 | 4501 | CACNA2D4 | 5969 | IPPK | 7437 | CAPN14 |
| 98 | SLC6A14 | 1566 | SIK1 | 3034 | TRIM6 | 4502 | KIF1B | 5970 | COL4A3 | 7438 | MTIF2 |
| 99 | AKR1A1 | 1567 | CEP112 | 3035 | MCC | 4503 | LAMB3 | 5971 | ISYNA1 | 7439 | CBWD1 |
| 100 | SLC35D2 | 1568 | RRN3 | 3036 | SMC1A | 4504 | REV1 | 5972 | VSX2 | 7440 | TYK2 |
| 101 | CRKL | 1569 | HINFP | 3037 | GPR135 | 4505 | N4BP2 | 5973 | POLR3C | 7441 | C14ORF80 |
| 102 | SCRG1 | 1570 | TFPT | 3038 | PRPH | 4506 | SLC6A11 | 5974 | SUPV3L1 | 7442 | NRXN1 |
| 103 | DHX32 | 1571 | ANKRD54 | 3039 | PCDHGB6 | 4507 | CCDC40 | 5975 | NFRKB | 7443 | DNAJC16 |
| 104 | MSMO1 | 1572 | TMED7-TICAM2 | 3040 | LOC284014 | 4508 | COL17A1 | 5976 | IPO9 | 7444 | TTC29 |
| 105 | C17ORF59 | 1573 | KIAA0930 | 3041 | LOC646736 | 4509 | HAVCR1 | 5977 | SLC8B1 | 7445 | MSANTD1 |
| 106 | C2ORF49 | 1574 | C4ORF3 | 3042 | NLRP11 | 4510 | TONSL | 5978 | CACNA2D3 | 7446 | DZIP1 |
| 107 | ELF5 | 1575 | NAP1L4 | 3043 | POLDIP3 | 4511 | RHBDF2 | 5979 | MEF2D | 7447 | EPHA4 |
| 108 | SNAP47 | 1576 | PQLC1 | 3044 | PKP2 | 4512 | PPM1J | 5980 | SASS6 | 7448 | RP11-1008C21.2 |
| 109 | LUZP1 | 1577 | P4HTM | 3045 | LOC153811 | 4513 | TMEM38B | 5981 | RP11-802E16.3 | 7449 | SGCA |
| 110 | RAN | 1578 | TRAK2 | 3046 | NBPF15 | 4514 | USP45 | 5982 | DNAH11 | 7450 | FBXO41 |
| 111 | MOAP1 | 1579 | CKLF | 3047 | WNT1 | 4515 | IQGAP2 | 5983 | ARID3B | 7451 | VGLL4 |
| 112 | HBA2 | 1580 | PSD3 | 3048 | SYCE2 | 4516 | KCNN4 | 5984 | RYR1 | 7452 | CRHR1-IT1 |
| 113 | SPP1 | 1581 | TMEM120B | 3049 | MAGEB3 | 4517 | AC114730.2 | 5985 | PARD3B | 7453 | HDAC9 |
| 114 | C1ORF51 | 1582 | REL | 3050 | TET2 | 4518 | SPTA1 | 5986 | SNED1 | 7454 | USPL1 |
| 115 | SOX7 | 1583 | EIF3K | 3051 | HIST1H2BN | 4519 | SLC25A13 | 5987 | RBBP5 | 7455 | FARP2 |
| 116 | PFAS | 1584 | DOK5 | 3052 | RBM12B | 4520 | C9ORF91 | 5988 | HSF2BP | 7456 | ASS1 |
| 117 | DCTD | 1585 | DDAH1 | 3053 | LOC100128139 | 4521 | GEM | 5989 | MIR4657 | 7457 | UBR5 |
| 118 | HOXD1 | 1586 | NFKBIB | 3054 | OR11H12 | 4522 | CACNB3 | 5990 | MND1 | 7458 | NEK2 |
| 119 | STEAP1 | 1587 | ATP6V1A | 3055 | ZNF483 | 4523 | MRPS5 | 5991 | NSMF | 7459 | TEK |
| 120 | GREM1 | 1588 | SCNN1B | 3056 | THAP5 | 4524 | SPATS2 | 5992 | DSC1 | 7460 | ATP1A2 |
| 121 | PRDX3 | 1589 | RILP | 3057 | AKAP11 | 4525 | DSG4 | 5993 | ARL13A | 7461 | HSF4 |
| 122 | INSIG2 | 1590 | PDIA4 | 3058 | D21S2088E | 4526 | TCERG1L | 5994 | LOC101927354 | 7462 | CREB3L1 |
| 123 | SPATA2L | 1591 | CASD1 | 3059 | PPYR1 | 4527 | USP24 | 5995 | CRISPLD2 | 7463 | ZFR2 |
| 124 | JAM3 | 1592 | SNX19 | 3060 | ZNF407 | 4528 | KLHL24 | 5996 | UGT3A2 | 7464 | POC5 |
| 125 | TCP1 | 1593 | JTB | 3061 | TOM1L2 | 4529 | PPP1R13B | 5997 | OSER1 | 7465 | CCDC104 |
| 126 | PSMD10 | 1594 | MAN2A1 | 3062 | CCDC6 | 4530 | AHRR | 5998 | RP11-13P5.1 | 7466 | VWDE |
| 127 | LANCL1 | 1595 | FST | 3063 | CCDC76 | 4531 | SCO2 | 5999 | HSFY1 | 7467 | CCDC169-SOHLH2 |
| 128 | GPR152 | 1596 | TAF11 | 3064 | HPX-2 | 4532 | GFRA2 | 6000 | UBALD2 | 7468 | CTD-2019O4.1 |
| 129 | ECH1 | 1597 | TTC39A | 3065 | C14ORF28 | 4533 | VCAN-AS1 | 6001 | FAM174B | 7469 | PRMT3 |
| 130 | DUSP5 | 1598 | USP34 | 3066 | EPB41 | 4534 | FBXW2 | 6002 | STRN4 | 7470 | SDK1 |
| 131 | NOTCH1 | 1599 | WFDC2 | 3067 | GPX5 | 4535 | CASP1 | 6003 | TFDP1 | 7471 | ARHGEF1 |
| 132 | SLC25A6 | 1600 | KLF11 | 3068 | SLC19A1 | 4536 | RP11-153M7.3 | 6004 | TRERF1 | 7472 | STIL |
| 133 | BRMS1L | 1601 | NSFL1C | 3069 | STARD13 | 4537 | SPTLC3 | 6005 | CAMK1D | 7473 | VGLL3 |
| 134 | HK3 | 1602 | TMEM209 | 3070 | KLHL28 | 4538 | CAMKK1 | 6006 | PNPLA8 | 7474 | SPATA17 |
| 135 | LARP1B | 1603 | NFKB1 | 3071 | PTPN14 | 4539 | NRIP3 | 6007 | TMEM116 | 7475 | SCAPER |
| 136 | FAM8A1 | 1604 | NAALADL1 | 3072 | LOC100129447 | 4540 | ACACA | 6008 | FNDC3A | 7476 | ENAH |
| 137 | GPAA1 | 1605 | TECR | 3073 | RFT1 | 4541 | CCDC146 | 6009 | RDH12 | 7477 | PASK |
| 138 | C1ORF43 | 1606 | FLJ31715 | 3074 | RBM6 | 4542 | SFI1 | 6010 | RASSF8-AS1 | 7478 | SUDS3 |
| 139 | NDUFS2 | 1607 | C5ORF44 | 3075 | METTL19 | 4543 | SSBP2 | 6011 | C1RL | 7479 | GATA4 |
| 140 | SRF | 1608 | NME1 | 3076 | LOC144742 | 4544 | SLC47A2 | 6012 | CALCB | 7480 | EDAR |
| 141 | IGF1 | 1609 | TUBA4A | 3077 | PVRL4 | 4545 | ANAPC4 | 6013 | RPS7 | 7481 | LRTOMT |
| 142 | SLC7A11 | 1610 | SMUG1 | 3078 | TAS2R30 | 4546 | RP11-519G16.3 | 6014 | FAIM2 | 7482 | LOC643201 |
| 143 | ARMCX6 | 1611 | ADCK4 | 3079 | HIST1H4A | 4547 | MKNK1 | 6015 | HMGA2 | 7483 | LOC101928791 |
| 144 | SDS | 1612 | GNG2 | 3080 | IGFALS | 4548 | RBM23 | 6016 | C7ORF50 | 7484 | C3P1 |
| 145 | RAB34 | 1613 | G3BP2 | 3081 | ALMS1P | 4549 | LMOD1 | 6017 | MAP1S | 7485 | CCT2 |
| 146 | CCL18 | 1614 | PACRGL | 3082 | TRPM3 | 4550 | NR2C1 | 6018 | NUBP1 | 7486 | STARD8 |
| 147 | SEMA4B | 1615 | INHBA | 3083 | PTPRS | 4551 | STXBP5 | 6019 | CHAF1B | 7487 | AADAT |
| 148 | NDUFA4L2 | 1616 | SRSF7 | 3084 | LOC100130522 | 4552 | MUS81 | 6020 | PLCL2 | 7488 | SMYD3 |
| 149 | ARPC1B | 1617 | MIS12 | 3085 | TTN | 4553 | TLN2 | 6021 | GOLGA8I | 7489 | PBRM1 |
| 150 | PARL | 1618 | SORBS2 | 3086 | IRF2BP1 | 4554 | SMU1 | 6022 | DOPEY1 | 7490 | PRR5L |
| 151 | CYCS | 1619 | CYBA | 3087 | C1ORF98 | 4555 | UNC5B | 6023 | C17ORF75 | 7491 | PAM |
| 152 | LOC100132831 | 1620 | LOC284889 | 3088 | MYOCD | 4556 | TPRA1 | 6024 | KCNIP1 | 7492 | EHBP1L1 |
| 153 | SNRK | 1621 | C9ORF167 | 3089 | TNK2 | 4557 | CHST7 | 6025 | CSTA | 7493 | NOC3L |
| 154 | HTRA3 | 1622 | PRDX4 | 3090 | CLLU1OS | 4558 | ATP1A4 | 6026 | WDR66 | 7494 | TJP1 |
| 155 | HLA-DPB1 | 1623 | NTHL1 | 3091 | GKN1 | 4559 | LRIG1 | 6027 | PRRG3 | 7495 | LOC101928767 |
| 156 | C19ORF28 | 1624 | JAKMIP2 | 3092 | LMTK2 | 4560 | TESK2 | 6028 | ECI2 | 7496 | PBX4 |
| 157 | APOBEC3A | 1625 | UCP2 | 3093 | FLJ31306 | 4561 | APPL2 | 6029 | KLHDC2 | 7497 | L1CAM |
| 158 | MPPE1 | 1626 | VPS13C | 3094 | PTPRK | 4562 | DCBLD1 | 6030 | EVI2B | 7498 | ACAN |
| 159 | TSPAN19 | 1627 | DRAM2 | 3095 | TBX15 | 4563 | HS3ST3B1 | 6031 | TMEM229B | 7499 | TFCP2L1 |
| 160 | AGTRAP | 1628 | PIEZO2 | 3096 | LOC642648 | 4564 | RNF207 | 6032 | CR2 | 7500 | AMIGO2 |
| 161 | KLHDC8B | 1629 | EGLN2 | 3097 | SLC25A34 | 4565 | TMEM45B | 6033 | CT45A1 | 7501 | GLIS1 |
| 162 | NCOA3 | 1630 | GPRIN3 | 3098 | ALDH7A1 | 4566 | FKBP10 | 6034 | LHX3 | 7502 | NAA10 |
| 163 | HLA-DRB6 | 1631 | ABCF1 | 3099 | FBRS | 4567 | NTAN1 | 6035 | MRPL24 | 7503 | CCDC136 |
| 164 | SNIP1 | 1632 | SAMM50 | 3100 | HIST1H4L | 4568 | CBS | 6036 | CEP164 | 7504 | LINC00349 |
| 165 | LYRM1 | 1633 | FCGR2A | 3101 | CRABP2 | 4569 | AKAP8 | 6037 | CCDC50 | 7505 | IGSF22 |
| 166 | OLFML3 | 1634 | S100A16 | 3102 | PRRT4 | 4570 | COL15A1 | 6038 | FLYWCH2 | 7506 | DLGAP2 |
| 167 | TUBA1A | 1635 | NME4 | 3103 | LOC100128191 | 4571 | FCER2 | 6039 | RP11-606D9.1 | 7507 | BACE2 |
| 168 | CMPK1 | 1636 | FKBP1A | 3104 | C20ORF96 | 4572 | TBCA | 6040 | CD74 | 7508 | LLGL2 |
| 169 | IGLL1 | 1637 | KLHL8 | 3105 | SLC28A1 | 4573 | PAK1 | 6041 | SIK2 | 7509 | WDR60 |
| 170 | APEH | 1638 | GBA2 | 3106 | GON4L | 4574 | ATF6 | 6042 | ZNF844 | 7510 | GOLGA8A |
| 171 | MAP2K1 | 1639 | ZNF414 | 3107 | LOC100132731 | 4575 | KIFC3 | 6043 | CTNNBIP1 | 7511 | LOC101927144 |
| 172 | BAP1 | 1640 | CHST2 | 3108 | MUC5AC | 4576 | ADAMTSL1 | 6044 | ZKSCAN7 | 7512 | BAHCC1 |
| 173 | ANKIB1 | 1641 | IFITM4P | 3109 | PNPLA5 | 4577 | SYNJ2 | 6045 | MRC2 | 7513 | WNT7A |
| 174 | GBP1 | 1642 | CYBRD1 | 3110 | CDR1 | 4578 | TOP2A | 6046 | ECEL1 | 7514 | ARHGAP24 |
| 175 | PNMA1 | 1643 | SNX3 | 3111 | PPCDC | 4579 | SPINK5 | 6047 | CPNE1 | 7515 | ETV2 |
| 176 | CYBASC3 | 1644 | ELMOD2 | 3112 | LPIN3 | 4580 | PEX1 | 6048 | PCSK7 | 7516 | STK35 |
| 177 | LDHA | 1645 | ING2 | 3113 | LRRTM2 | 4581 | LRIG3 | 6049 | CA9 | 7517 | FAM65B |
| 178 | CD1A | 1646 | POTEKP | 3114 | ZNF697 | 4582 | OSBPL8 | 6050 | TMEM120A | 7518 | CCT7 |
| 179 | SERPINA3 | 1647 | SLC23A2 | 3115 | IL1RL2 | 4583 | MYO18B | 6051 | RUFY4 | 7519 | ATG4C |
| 180 | NELF | 1648 | DDX60 | 3116 | SCML4 | 4584 | ADAMTS7P1 | 6052 | LPA | 7520 | L3MBTL3 |
| 181 | FTL | 1649 | KIAA1656 | 3117 | KIAA1683 | 4585 | CACHD1 | 6053 | ITGB1BP2 | 7521 | FAM65C |
| 182 | FDPSL2A | 1650 | UBE2L6 | 3118 | ACTL8 | 4586 | MGAT4B | 6054 | SLAMF1 | 7522 | RP11-149P24.1 |
| 183 | SMPD1 | 1651 | SCYL2 | 3119 | FLJ42627 | 4587 | STAT6 | 6055 | NFXL1 | 7523 | KIAA0895 |
| 184 | PRDX1 | 1652 | TRPC7 | 3120 | LOC170425 | 4588 | SH3BP1 | 6056 | C9ORF89 | 7524 | CA10 |
| 185 | DFNA5 | 1653 | ACSM3 | 3121 | ENTPD2 | 4589 | RBM44 | 6057 | CACNA2D3-AS1 | 7525 | NDUFAF7 |
| 186 | RABGGTA | 1654 | RIOK1 | 3122 | PTCH1 | 4590 | SERPINI1 | 6058 | TIMD4 | 7526 | PLA2G4F |
| 187 | WDR61 | 1655 | SYNGR1 | 3123 | FAM106CP | 4591 | POLR1B | 6059 | TAPBPL | 7527 | CAPRIN2 |
| 188 | TRIM36 | 1656 | SPG21 | 3124 | SERP2 | 4592 | FYCO1 | 6060 | CTD-2337A12.1 | 7528 | HTR3D |
| 189 | COLEC11 | 1657 | SDHAF2 | 3125 | RAPGEF4 | 4593 | SLC7A5 | 6061 | LINC00960 | 7529 | FIG4 |
| 190 | FBXO22 | 1658 | AKIRIN1 | 3126 | LOC100653325 | 4594 | DIRC3 | 6062 | KRT12 | 7530 | KCNN1 |
| 191 | TUBA1C | 1659 | AP1AR | 3127 | GRIA1 | 4595 | WRAP73 | 6063 | DACT1 | 7531 | CD36 |
| 192 | ADCK1 | 1660 | PDCD2 | 3128 | TMSB15A | 4596 | LINC00862 | 6064 | RP1 | 7532 | TRMT1 |
| 193 | KIF7 | 1661 | LRR1 | 3129 | CEP68 | 4597 | STAR | 6065 | FBXW8 | 7533 | ATP2C2 |
| 194 | ESRRA | 1662 | CLPTM1L | 3130 | RCL1 | 4598 | LOC102546299 | 6066 | ABCG4 | 7534 | ENTPD5 |
| 195 | LSM4 | 1663 | PSME4 | 3131 | RNF39 | 4599 | TRAF1 | 6067 | SLC22A18 | 7535 | SCGB1A1 |
| 196 | PGS1 | 1664 | MAEA | 3132 | LOC100128366 | 4600 | SLC22A25 | 6068 | KRBOX1 | 7536 | PLEKHO2 |
| 197 | TAGLN2 | 1665 | HMGCL | 3133 | YY2 | 4601 | KPNA1 | 6069 | TNMD | 7537 | LAMB1 |
| 198 | PSMC3 | 1666 | STAT3 | 3134 | ZNF589 | 4602 | NAV2 | 6070 | HMGN2P15 | 7538 | DHRS1 |
| 199 | PPARG | 1667 | GPX1 | 3135 | CPLX2 | 4603 | POLG | 6071 | ZNF568 | 7539 | IDE |
| 200 | ACSF3 | 1668 | SFT2D1 | 3136 | ANK3 | 4604 | RRN3P2 | 6072 | MIA2 | 7540 | SENP3-EIF4A1 |
| 201 | WLS | 1669 | DKK3 | 3137 | KLF7 | 4605 | CEP70 | 6073 | SLC25A1 | 7541 | MYBBP1A |
| 202 | DPYSL4 | 1670 | PLP2 | 3138 | SUGP2 | 4606 | PHTF1 | 6074 | C7ORF43 | 7542 | LOC100507562 |
| 203 | PANK4 | 1671 | NAT1 | 3139 | NID2 | 4607 | NSRP1 | 6075 | TCFL5 | 7543 | IL7 |
| 204 | IL10RB | 1672 | TPSAB1 | 3140 | HDAC8 | 4608 | DENND4B | 6076 | FKBP15 | 7544 | PATZ1 |
| 205 | ETF1 | 1673 | GDE1 | 3141 | LAMB2P1 | 4609 | SWT1 | 6077 | ROPN1L | 7545 | TAF1C |
| 206 | CLPTM1 | 1674 | TBC1D5 | 3142 | RNU105A | 4610 | CLDN12 | 6078 | NOC4L | 7546 | DHX37 |
| 207 | MRFAP1 | 1675 | RANGRF | 3143 | OR10H1 | 4611 | WDR62 | 6079 | PIEZO1 | 7547 | KDM4B |
| 208 | SEMA3C | 1676 | TM9SF1 | 3144 | PALLD | 4612 | TBCE | 6080 | AC013733.3 | 7548 | OPRM1 |
| 209 | UBE2J2 | 1677 | CCDC69 | 3145 | OPCML | 4613 | RBPMS | 6081 | LIMCH1 | 7549 | IFNAR2 |
| 210 | CTHRC1 | 1678 | FADS6 | 3146 | ZNF792 | 4614 | WDR59 | 6082 | LIFR | 7550 | CCDC108 |
| 211 | JMJD6 | 1679 | GNB5 | 3147 | ZDHHC22 | 4615 | ARMC4 | 6083 | LPAR2 | 7551 | DST |
| 212 | ACTG1 | 1680 | THG1L | 3148 | PCDHA5 | 4616 | QKI | 6084 | ACP1 | 7552 | NAT14 |
| 213 | ALS2 | 1681 | AKTIP | 3149 | PCDHA6 | 4617 | NAV1 | 6085 | TM2D2 | 7553 | TMOD1 |
| 214 | ZNF184 | 1682 | C9ORF46 | 3150 | LOC100288069 | 4618 | GBE1 | 6086 | VTN | 7554 | STIM1 |
| 215 | C8ORF4 | 1683 | BAZ2B | 3151 | TMEM26 | 4619 | BAIAP2L1 | 6087 | ZNF304 | 7555 | RNF128 |
| 216 | GRTP1 | 1684 | HEATR5A | 3152 | LPHN1 | 4620 | GALT | 6088 | TRIB3 | 7556 | SYT1 |
| 217 | PRRG1 | 1685 | PFKP | 3153 | THUMPD2 | 4621 | MN1 | 6089 | LAMA1 | 7557 | CERS4 |
| 218 | E2F4 | 1686 | HSD17B4 | 3154 | GGTLC1 | 4622 | QSER1 | 6090 | PPM1F | 7558 | ATG16L1 |
| 219 | OBFC2B | 1687 | FPR3 | 3155 | LOC283922 | 4623 | ARPP21 | 6091 | LINC00320 | 7559 | GULP1 |
| 220 | KRT17 | 1688 | EPRS | 3156 | LOC643406 | 4624 | KIF23 | 6092 | UNC13A | 7560 | IGKV1D-27 |
| 221 | VWA1 | 1689 | MANSC1 | 3157 | CHD5 | 4625 | OGDH | 6093 | R3HCC1 | 7561 | OSBPL9 |
| 222 | RPLP0 | 1690 | SLC39A7 | 3158 | CNNM3 | 4626 | FCER1A | 6094 | CLCA1 | 7562 | PTPRF |
| 223 | ARF6 | 1691 | NFKBID | 3159 | PLEKHA6 | 4627 | RBKS | 6095 | CCDC163P | 7563 | ATP4A |
| 224 | COX4I1 | 1692 | RPS18 | 3160 | CIITA | 4628 | METTL3 | 6096 | TMTC2 | 7564 | HP |
| 225 | CES1P2 | 1693 | RAF1 | 3161 | KU-MEL-3 | 4629 | LTBP1 | 6097 | COMMD3-BMI1 | 7565 | LOC101929551 |
| 226 | WDR46 | 1694 | LOC284023 | 3162 | C1ORF195 | 4630 | ALDH1L1 | 6098 | FRZB | 7566 | IGFBP2 |
| 227 | CSRNP1 | 1695 | RIPK3 | 3163 | ZHX3 | 4631 | SHANK2 | 6099 | GPI | 7567 | CNGA4 |
| 228 | MCOLN1 | 1696 | CAND1 | 3164 | PYHIN1 | 4632 | TSPAN7 | 6100 | SLC6A4 | 7568 | CCAR2 |
| 229 | TNPO1 | 1697 | PSMD13 | 3165 | PRKACG | 4633 | POLN | 6101 | C1ORF185 | 7569 | NARS2 |
| 230 | PCDH12 | 1698 | CASC4 | 3166 | LOC100289090 | 4634 | F13A1 | 6102 | G6PC2 | 7570 | NOL4L |
| 231 | PRKAG1 | 1699 | MYOF | 3167 | CXORF65 | 4635 | RALGAPA2 | 6103 | PDLIM2 | 7571 | TNIK |
| 232 | C19ORF59 | 1700 | PPP5C | 3168 | ZNF705G | 4636 | ICA1L | 6104 | ADRM1 | 7572 | LINC00710 |
| 233 | PACSIN3 | 1701 | FBXO5 | 3169 | FLJ14186 | 4637 | MICAL1 | 6105 | TEX13A | 7573 | EFNA4 |
| 234 | PTPRZ1 | 1702 | PAN3 | 3170 | MATL2963 | 4638 | CST7 | 6106 | STMN1 | 7574 | BCL11B |
| 235 | FABP4 | 1703 | MFSD10 | 3171 | ZNF676 | 4639 | FHDC1 | 6107 | IQSEC2 | 7575 | TPD52L1 |
| 236 | ZNF323 | 1704 | H1FX | 3172 | ZFHX4 | 4640 | VNN1 | 6108 | ZFP69B | 7576 | BRF1 |
| 237 | SEC22C | 1705 | CTR9 | 3173 | LIMS3L | 4641 | SMARCA2 | 6109 | CAMSAP3 | 7577 | MIPEP |
| 238 | NR1H2 | 1706 | SEPW1 | 3174 | FOXE3 | 4642 | ASTN1 | 6110 | CD300LG | 7578 | NT5C |
| 239 | C20ORF27 | 1707 | DHCR7 | 3175 | LOC286087 | 4643 | KIF19 | 6111 | AQP1 | 7579 | KLHL5 |
| 240 | IGLL5 | 1708 | FEZ2 | 3176 | MTUS1 | 4644 | MEI1 | 6112 | ERICH6 | 7580 | FRMPD2 |
| 241 | MRPS7 | 1709 | ADPRHL2 | 3177 | C4ORF21 | 4645 | ARPC3 | 6113 | MAP4K1 | 7581 | HOXA7 |
| 242 | RAC1 | 1710 | TBC1D12 | 3178 | DRD4 | 4646 | CCDC67 | 6114 | ACHE | 7582 | DNAH12 |
| 243 | PHLDA1 | 1711 | DTNBP1 | 3179 | LRRC4B | 4647 | SLC16A10 | 6115 | USP4 | 7583 | C21ORF58 |
| 244 | HTR2B | 1712 | ABHD13 | 3180 | LOC729966 | 4648 | DGKK | 6116 | VIPR2 | 7584 | NELFA |
| 245 | FBLN1 | 1713 | ENDOD1 | 3181 | BARHL1 | 4649 | INTS2 | 6117 | RASL11B | 7585 | DCDC5 |
| 246 | KLF10 | 1714 | SP2 | 3182 | LOC150381 | 4650 | CLIC5 | 6118 | STX18-AS1 | 7586 | PLOD1 |
| 247 | TTC38 | 1715 | SLC1A5 | 3183 | SHBG | 4651 | SCUBE1 | 6119 | ZNF195 | 7587 | HLA-A |
| 248 | PCDH17 | 1716 | ARFRP1 | 3184 | FLJ41484 | 4652 | EMX1 | 6120 | ATP8B1 | 7588 | COL25A1 |
| 249 | LOC731424 | 1717 | PAGE2B | 3185 | LOC729041 | 4653 | THRA | 6121 | EFEMP2 | 7589 | HLA-DQA1 |
| 250 | ARF1 | 1718 | RPS9 | 3186 | LOC100147773 | 4654 | DMXL1 | 6122 | CTTNBP2 | 7590 | KCNQ4 |
| 251 | SLC10A4 | 1719 | VPS52 | 3187 | ADAMTS19 | 4655 | LINC00293 | 6123 | C1ORF141 | 7591 | MBD6 |
| 252 | SDHB | 1720 | USP3 | 3188 | LPHN2 | 4656 | ETFA | 6124 | SLC22A6 | 7592 | FOLR3 |
| 253 | HAS2 | 1721 | KIAA0196 | 3189 | ABLIM3 | 4657 | RP11-324O2.3 | 6125 | EFNA1 | 7593 | NLRC3 |
| 254 | GNB2 | 1722 | PAFAH1B2 | 3190 | MAP4 | 4658 | PPP1R12A | 6126 | MAN1C1 | 7594 | ANKRD26 |
| 255 | CA12 | 1723 | ARRDC3 | 3191 | HS1BP3 | 4659 | CLUH | 6127 | MAPK3 | 7595 | SPG7 |
| 256 | CHPF2 | 1724 | CD1C | 3192 | DCLK2 | 4660 | MFN1 | 6128 | CENPA | 7596 | CTNND2 |
| 257 | DCTN3 | 1725 | GORASP1 | 3193 | ZIK1 | 4661 | MEGF8 | 6129 | PEX6 | 7597 | LOC105374809 |
| 258 | SIRT2 | 1726 | POLA2 | 3194 | IRX3 | 4662 | LTBP2 | 6130 | XXYAC-YM21GA2.3 | 7598 | LOC101929219 |
| 259 | POLD2 | 1727 | MAPRE1 | 3195 | SASH1 | 4663 | ST18 | 6131 | SSFA2 | 7599 | SMIM1 |
| 260 | COMP | 1728 | CCND2 | 3196 | RGMB | 4664 | CREM | 6132 | PXYLP1 | 7600 | MIR612 |
| 261 | HBD | 1729 | RAD23B | 3197 | SERPINB7 | 4665 | MPP2 | 6133 | ARSB | 7601 | LOC100505650 |
| 262 | ODC1 | 1730 | FBXO28 | 3198 | AKAP9 | 4666 | LRP12 | 6134 | ANKMY2 | 7602 | RICTOR |
| 263 | KBTBD2 | 1731 | TSTA3 | 3199 | GUSBP1 | 4667 | TRPC4 | 6135 | TMEM261 | 7603 | FMR1 |
| 264 | IRF4 | 1732 | ADSS | 3200 | CROT | 4668 | MTHFD2L | 6136 | KLF8 | 7604 | RABEP1 |
| 265 | CYTL1 | 1733 | ADNP | 3201 | LOC389906 | 4669 | RP11-557H15.3 | 6137 | AURKC | 7605 | SNORD73A |
| 266 | MYC | 1734 | PLCB1 | 3202 | LOC100132249 | 4670 | GBP1P1 | 6138 | MED6 | 7606 | SP3 |
| 267 | PLA2G7 | 1735 | USP8 | 3203 | NXN | 4671 | BAI1 | 6139 | CCNB3 | 7607 | TIA1 |
| 268 | ENG | 1736 | PID1 | 3204 | LOC100128001 | 4672 | FGGY | 6140 | PLK1 | 7608 | CLEC4D |
| 269 | CAPG | 1737 | C15ORF48 | 3205 | SPACA1 | 4673 | FAM151B | 6141 | IDUA | 7609 | CARD8 |
| 270 | USP19 | 1738 | SH2B1 | 3206 | RC3H2 | 4674 | SEL1L3 | 6142 | PRR26 | 7610 | MARCHF1 |
| 271 | PGD | 1739 | MORC2 | 3207 | IGF1R | 4675 | NMU | 6143 | TRIQK | 7611 | CHD2 |
| 272 | RAB9BP1 | 1740 | FLII | 3208 | C14ORF48 | 4676 | SLC41A2 | 6144 | TCEB2 | 7612 | PPIP5K2 |
| 273 | KIDINS220 | 1741 | UFC1 | 3209 | C17ORF53 | 4677 | TRAPPC9 | 6145 | SESTD1 | 7613 | HMGB1 |
| 274 | ERRFI1 | 1742 | KDR | 3210 | LOC728437 | 4678 | LRBA | 6146 | SRRM4 | 7614 | SUB1 |
| 275 | C18ORF32 | 1743 | SOCS3 | 3211 | ZMAT1 | 4679 | ILF3-AS1 | 6147 | ANGPT2 | 7615 | CRISP3 |
| 276 | CNOT8 | 1744 | NAT2 | 3212 | NCK2 | 4680 | LRRC9 | 6148 | SLC39A5 | 7616 | UBE2J1 |
| 277 | SPAG7 | 1745 | ELOVL6 | 3213 | LOC728316 | 4681 | LCN10 | 6149 | KCNQ5 | 7617 | UBA3 |
| 278 | SFRP2 | 1746 | GDI2 | 3214 | POM121 | 4682 | JAZF1-AS1 | 6150 | TGFB3 | 7618 | C1ORF186 |
| 279 | PTCHD1 | 1747 | FHL2 | 3215 | KANK3 | 4683 | TGM1 | 6151 | RP11-700E23.2 | 7619 | NEAT1 |
| 280 | ZFP91 | 1748 | PAPSS2 | 3216 | AGSK1 | 4684 | NSUN4 | 6152 | CEP135 | 7620 | PHIP |
| 281 | TM7SF2 | 1749 | THADA | 3217 | MAN1A2 | 4685 | IL6R | 6153 | PGRMC2 | 7621 | PARP8 |
| 282 | ATP2C1 | 1750 | ZNF274 | 3218 | DIAPH1 | 4686 | FAM101B | 6154 | BST1 | 7622 | ZNF267 |
| 283 | DDX21 | 1751 | ATXN1L | 3219 | ADAM7 | 4687 | NUP188 | 6155 | PGAP3 | 7623 | CHURC1 |
| 284 | MAN2B1 | 1752 | LOC100128252 | 3220 | WNT9A | 4688 | GRIN1 | 6156 | LOC100129637 | 7624 | CAPZA2 |
| 285 | DNAJA1 | 1753 | PRKACB | 3221 | GPSM1 | 4689 | DIAPH3 | 6157 | FAM63A | 7625 | MCTP1 |
| 286 | MFAP2 | 1754 | ZFP36 | 3222 | C2ORF51 | 4690 | CCDC186 | 6158 | SLFNL1-AS1 | 7626 | CLEC7A |
| 287 | DENND1B | 1755 | LYRM4 | 3223 | DSEL | 4691 | CD82 | 6159 | LINC00207 | 7627 | FAM76B |
| 288 | CRY1 | 1756 | GCOM1 | 3224 | LOC728178 | 4692 | C10ORF55 | 6160 | ICAM4 | 7628 | RASA2 |
| 289 | MAF1 | 1757 | SLC4A7 | 3225 | BAGE4 | 4693 | DALRD3 | 6161 | PPIL4 | 7629 | CEACAM8 |
| 290 | KDM3A | 1758 | RPL10 | 3226 | ZNF667 | 4694 | ITIH4 | 6162 | GRID1 | 7630 | CRBN |
| 291 | KIF11 | 1759 | GOLT1B | 3227 | RNU1-5 | 4695 | CKAP2L | 6163 | MPPED1 | 7631 | NAMPT |
| 292 | ZNF227 | 1760 | GSTO2 | 3228 | TLE4 | 4696 | ABCB4 | 6164 | ZNRD1-AS1 | 7632 | ITGAV |
| 293 | BTD | 1761 | LOC285000 | 3229 | ATP8B2 | 4697 | MYBL2 | 6165 | TSR2 | 7633 | TMX1 |
| 294 | ELOF1 | 1762 | CYP2S1 | 3230 | SFMBT2 | 4698 | LTBP4 | 6166 | VTCN1 | 7634 | CLK4 |
| 295 | CYHR1 | 1763 | SPAST | 3231 | ACIN1 | 4699 | ITSN1 | 6167 | PGBD5 | 7635 | ZRANB2 |
| 296 | CD93 | 1764 | PRICKLE3 | 3232 | KRTAP10-10 | 4700 | EHMT2 | 6168 | MYO7B | 7636 | RGS18 |
| 297 | VSIG1 | 1765 | AKR7A2P1 | 3233 | BRSK1 | 4701 | LTF | 6169 | FAM13A-AS1 | 7637 | SLPI |
| 298 | WDR47 | 1766 | TMEM131 | 3234 | GAGE1 | 4702 | CAB39L | 6170 | GUCD1 | 7638 | COX7B |
| 299 | ST3GAL4 | 1767 | B9D1 | 3235 | DNM3 | 4703 | FAM110A | 6171 | TOMM6 | 7639 | FAM126B |
| 300 | P4HB | 1768 | MIR155HG | 3236 | SUPT3H | 4704 | JADE2 | 6172 | RPS23 | 7640 | LRRN3 |
| 301 | MAPKAP1 | 1769 | C18ORF23 | 3237 | LOC100129397 | 4705 | ULK4 | 6173 | PRX | 7641 | TMEM176A |
| 302 | FOXM1 | 1770 | C11ORF73 | 3238 | OLFM1 | 4706 | FAM185A | 6174 | CPSF6 | 7642 | TMEM176B |
| 303 | CD79B | 1771 | ROR2 | 3239 | GLIPR1L2 | 4707 | LINC00877 | 6175 | PHOSPHO2 | 7643 | BTNL3 |
| 304 | PCCB | 1772 | RGS16 | 3240 | SEPT9 | 4708 | ASIC2 | 6176 | MGST2 | 7644 | CXCL12 |
| 305 | SLC2A3 | 1773 | HIC1 | 3241 | LAIR2 | 4709 | TRIM3 | 6177 | CHIA | 7645 | GOLIM4 |
| 306 | FILIP1L | 1774 | UTS2R | 3242 | SH3RF2 | 4710 | EXOSC10 | 6178 | HNRNPF | 7646 | IGHD |
| 307 | SNUPN | 1775 | C9ORF16 | 3243 | SLC34A3 | 4711 | STRBP | 6179 | PDE1B | 7647 | ERAP2 |
| 308 | PLLP | 1776 | PPP2CB | 3244 | SULT4A1 | 4712 | MROH2A | 6180 | PLEKHD1 | 7648 | MIR675 |
| 309 | RGS1 | 1777 | CLSTN3 | 3245 | CHST4 | 4713 | SCART1 | 6181 | MIR1237 | 7649 | IGLC1 |
| 310 | INO80 | 1778 | CIB4 | 3246 | GABRE | 4714 | COL16A1 | 6182 | TEKT1 | 7650 | IGHV4-31 |
| 311 | RNLS | 1779 | ATP6V0E2 | 3247 | TTC39B | 4715 | VAV2 | 6183 | RP11-211G23.1 | 7651 | MEDAG |
| 312 | CHN1 | 1780 | GOLT1A | 3248 | GPC6 | 4716 | TTLL9 | 6184 | PPFIBP2 | 7652 | IL1R2 |
| 313 | CANT1 | 1781 | SEC24A | 3249 | PDE4DIP | 4717 | FHOD1 | 6185 | SPAG4 | 7653 | MT1M |
| 314 | PIGW | 1782 | CBWD5 | 3250 | NPFF | 4718 | OPA1 | 6186 | DVL2 | 7654 | CCL11 |
| 315 | HADHA | 1783 | HPS3 | 3251 | SSH2 | 4719 | MVK | 6187 | BIRC7 | 7655 | WT1 |
| 316 | TXNL4B | 1784 | RBP1 | 3252 | KIAA1671 | 4720 | SLAMF7 | 6188 | S100A13 | 7656 | AVPR1A |
| 317 | AKR7A2 | 1785 | TRIM37 | 3253 | ADCY2 | 4721 | CLEC1B | 6189 | GLDC | 7657 | FCAR |
| 318 | PPP4R4 | 1786 | TMED3 | 3254 | NXF3 | 4722 | AVL9 | 6190 | KLHDC3 | 7658 | GFPT2 |
| 319 | CALM1 | 1787 | LOC100129196 | 3255 | IRX2 | 4723 | BPIFB1 | 6191 | GSE1 | 7659 | MT1G |
| 320 | PNMA2 | 1788 | TIGD6 | 3256 | SLC4A1 | 4724 | CHKA | 6192 | TCTE1 | 7660 | MT1X |
| 321 | HERC6 | 1789 | RN28S1 | 3257 | HDAC11 | 4725 | PRKCB | 6193 | TMPRSS5 | 7661 | ANKRD22 |
| 322 | DVL1 | 1790 | KRT76 | 3258 | MRPS17 | 4726 | EFCAB12 | 6194 | ETS1 | 7662 | GPR171 |
| 323 | CRYM | 1791 | LRCH1 | 3259 | MLLT4 | 4727 | DEDD | 6195 | SURF2 | 7663 | NTRK2 |
| 324 | MRPL40 | 1792 | FAM125A | 3260 | BNIP3L | 4728 | NPHS1 | 6196 | FLJ41757 | 7664 | S100P |
| 325 | CCND1 | 1793 | BRWD1 | 3261 | C9ORF84 | 4729 | ARHGEF7 | 6197 | PAX8 | 7665 | ELL2 |
| 326 | ITGB5 | 1794 | LOC143188 | 3262 | PIK3IP1 | 4730 | TEX33 | 6198 | MARCO | 7666 | CTSE |
| 327 | ZNF473 | 1795 | FLJ33544 | 3263 | ZNF625 | 4731 | CNTNAP5 | 6199 | SAP30 | 7667 | LOC100129518 |
| 328 | BCL2A1 | 1796 | CUBN | 3264 | GRIK3 | 4732 | CLIP4 | 6200 | EVA1C | 7668 | RFX2 |
| 329 | SLC7A1 | 1797 | TRIL | 3265 | CNN2 | 4733 | SEC24C | 6201 | C8ORF37-AS1 | 7669 | MT1F |
| 330 | LOC148413 | 1798 | WASF1 | 3266 | TMEM2 | 4734 | GNB3 | 6202 | FAM214A | 7670 | KCNE4 |
| 331 | ANKRD46 | 1799 | TCEANC2 | 3267 | ANKFY1 | 4735 | RPGRIP1 | 6203 | SLC2A4 | 7671 | CCDC71L |
| 332 | CBFB | 1800 | TMEM89 | 3268 | C11ORF42 | 4736 | SMG1P3 | 6204 | THAP9 | 7672 | HLA-DOB |
| 333 | STOML2 | 1801 | LOC100129617 | 3269 | CACNA1A | 4737 | AGPAT6 | 6205 | DGKG | 7673 | CLDN1 |
| 334 | MIF4GD | 1802 | ACSM2B | 3270 | C9ORF106 | 4738 | ASPM | 6206 | TMEM186 | 7674 | BDKRB1 |
| 335 | GALE | 1803 | ATF7IP | 3271 | H2AFB2 | 4739 | ATF2 | 6207 | AMPH | 7675 | LOC285043 |
| 336 | TMEM222 | 1804 | LOC648570 | 3272 | NEURL1B | 4740 | DGKA | 6208 | MIR205HG | 7676 | PPP1R3C |
| 337 | ASPN | 1805 | YY1AP1 | 3273 | C16ORF86 | 4741 | HCFC2 | 6209 | LOC100996291 | 7677 | PHEX |
| 338 | LDHB | 1806 | LRRC3C | 3274 | MRPS18C | 4742 | SGSM2 | 6210 | TCEB3 | 7678 | RAB12 |
| 339 | CD47 | 1807 | GABRA3 | 3275 | GBP6 | 4743 | RAB36 | 6211 | OR51B5 | 7679 | FLJ35700 |
| 340 | OR2A5 | 1808 | FLJ44477 | 3276 | LOC100129111 | 4744 | NUCB2 | 6212 | CPNE7 | 7680 | IGHA1 |
| 341 | LOC100134229 | 1809 | LGALS12 | 3277 | ST7L | 4745 | NMD3 | 6213 | RGS12 | 7681 | SOD2 |
| 342 | TXNDC16 | 1810 | C9ORF128 | 3278 | LOC284379 | 4746 | IL36G | 6214 | POLH | 7682 | CYORF15B |
| 343 | MGST1 | 1811 | IKBKB | 3279 | POLR2D | 4747 | TMEM234 | 6215 | WBSCR17 | 7683 | FLJ36031 |
| 344 | TRIM32 | 1812 | EXOSC6 | 3280 | LOC100652869 | 4748 | LINC-PINT | 6216 | ACSM4 | 7684 | SLITRK6 |
| 345 | RPS2 | 1813 | ZNF780B | 3281 | P2RY1 | 4749 | ARHGAP9 | 6217 | LOC101929529 | 7685 | BEX5 |
| 346 | C15ORF17 | 1814 | ZNF765 | 3282 | SLC10A7 | 4750 | TANC1 | 6218 | PSEN2 | 7686 | DLEC1 |
| 347 | PEG10 | 1815 | TTC9B | 3283 | FGD2 | 4751 | ZBTB10 | 6219 | ATP4B | 7687 | PNP |
| 348 | S100B | 1816 | ZNF843 | 3284 | PTCHD3 | 4752 | LEPRE1 | 6220 | NSMCE4A | 7688 | LRRC48 |
| 349 | DOHH | 1817 | MC3R | 3285 | UCP3 | 4753 | IQGAP3 | 6221 | PROSER1 | 7689 | PTGS2 |
| 350 | GLS2 | 1818 | EMILIN3 | 3286 | TAP2 | 4754 | MYH10 | 6222 | BAIAP3 | 7690 | IL1B |
| 351 | ZNF75D | 1819 | PLG | 3287 | IL1F10 | 4755 | RP11-162J8.3 | 6223 | TMEM128 | 7691 | IL8 |
| 352 | SF3B2 | 1820 | ZNF80 | 3288 | PAPLN | 4756 | DHX29 | 6224 | MT1H | 7692 | MUC4 |
| 353 | LAMC3 | 1821 | CFHR1 | 3289 | LOC727869 | 4757 | RHOF | 6225 | COMTD1 | 7693 | CXCL1 |
| 354 | PKIG | 1822 | OTOP1 | 3290 | LRRC37BP1 | 4758 | POLD1 | 6226 | ABCC3 | 7694 | S100A3 |
| 355 | PLCXD1 | 1823 | LOC730102 | 3291 | SLC5A12 | 4759 | CCDC64 | 6227 | ARHGEF17 | 7695 | CHST9 |
| 356 | PKN1 | 1824 | LOC100130357 | 3292 | ENTPD1 | 4760 | BAZ2A | 6228 | SIGLEC17P | 7696 | CDHR3 |
| 357 | SCTR | 1825 | FAM117B | 3293 | LOC339874 | 4761 | CREBRF | 6229 | MFAP5 | 7697 | TAC1 |
| 358 | CPD | 1826 | LOC100128496 | 3294 | HIST1H4E | 4762 | FTCD | 6230 | LDHD | 7698 | TTC18 |
| 359 | LRP10 | 1827 | SNORD3B-1 | 3295 | RN18S1 | 4763 | ZNF772 | 6231 | GPR98 | 7699 | FOSL1 |
| 360 | PRMT1 | 1828 | ROBO2 | 3296 | RDM1 | 4764 | UBE2Q1 | 6232 | DHX35 | 7700 | C1ORF192 |
| 361 | DAG1 | 1829 | NR6A1 | 3297 | DNAJC27-AS1 | 4765 | PRKCG | 6233 | ANKRD13B | 7701 | FAM183A |
| 362 | METAP2 | 1830 | PRTG | 3298 | SCARNA10 | 4766 | CDCA5 | 6234 | TNF | 7702 | VIPR1 |
| 363 | HPCAL1 | 1831 | HP1BP3 | 3299 | LOC645249 | 4767 | FAM115A | 6235 | SLC38A3 | 7703 | SOSTDC1 |
| 364 | ADC | 1832 | LOC90784 | 3300 | LZTS2 | 4768 | GABRP | 6236 | CASP10 | 7704 | MS4A8B |
| 365 | SYNDIG1L | 1833 | ARHGEF10 | 3301 | BRD3 | 4769 | ARMC6 | 6237 | PKD2L1 | 7705 | C9ORF24 |
| 366 | GARS | 1834 | TMEM30B | 3302 | HMX2 | 4770 | SFTPB | 6238 | MSRB1 | 7706 | C10ORF79 |
| 367 | TMEM9 | 1835 | AIM1L | 3303 | DCAF8 | 4771 | WIPI1 | 6239 | ZNF788 | 7707 | C20ORF85 |
| 368 | PSMA5 | 1836 | LOC100130285 | 3304 | TAB3 | 4772 | OVGP1 | 6240 | PNPLA3 | 7708 | FOXF1 |
| 369 | LAMA2 | 1837 | CDK15 | 3305 | LINC00328 | 4773 | TREML2 | 6241 | MTMR2 | 7709 | ACTG2 |
| 370 | DNASE2 | 1838 | LOC644794 | 3306 | GTF3C3 | 4774 | MCUR1 | 6242 | TCF7L2 | 7710 | CAPS |
| 371 | NSMCE1 | 1839 | MZF1 | 3307 | RYR2 | 4775 | CDH9 | 6243 | GTPBP10 | 7711 | TMEM100 |
| 372 | TRIM34 | 1840 | CNTNAP3B | 3308 | ST8SIA6 | 4776 | GPR132 | 6244 | CACTIN | 7712 | SMAD6 |
| 373 | LOC440934 | 1841 | SLFN5 | 3309 | THSD4 | 4777 | ITPR1 | 6245 | FAT1 | 7713 | SCGB3A1 |
| 374 | TMEM188 | 1842 | BGLAP | 3310 | LINC00426 | 4778 | TMEM59L | 6246 | ADAMTS10 | 7714 | SEMA3G |
| 375 | FAM108A1 | 1843 | WFIKKN2 | 3311 | RP1L1 | 4779 | KIAA1524 | 6247 | ARTN | 7715 | ZBBX |
| 376 | MVD | 1844 | LOC648044 | 3312 | C3ORF54 | 4780 | DAAM1 | 6248 | MSRB3 | 7716 | C15ORF23 |
| 377 | TRIM2 | 1845 | BCORP1 | 3313 | LOC401164 | 4781 | SERINC5 | 6249 | RINL | 7717 | STON1 |
| 378 | DAP | 1846 | WDR33 | 3314 | RIT2 | 4782 | MPP5 | 6250 | GRIA4 | 7718 | DLK2 |
| 379 | RIC8A | 1847 | ZNF717 | 3315 | MAFIP | 4783 | CEACAM1 | 6251 | CUL3 | 7719 | C1ORF94 |
| 380 | SLC46A2 | 1848 | SLC6A3 | 3316 | LOC100128126 | 4784 | SFTPD | 6252 | MPHOSPH8 | 7720 | SLC17A7 |
| 381 | LYL1 | 1849 | PCDHGB7 | 3317 | LOC440337 | 4785 | PPP1R3E | 6253 | TMEM184A | 7721 | SH3KBP1 |
| 382 | UTP11L | 1850 | TRIM41 | 3318 | FLG | 4786 | HBS1L | 6254 | EMR1 | 7722 | PRG2 |
| 383 | CPB2 | 1851 | LRFN3 | 3319 | IGSF9B | 4787 | SSRP1 | 6255 | CACNG7 | 7723 | IL6ST |
| 384 | NHEJ1 | 1852 | ATR | 3320 | ZNF846 | 4788 | BANK1 | 6256 | NME7 | 7724 | TMEM143 |
| 385 | CYC1 | 1853 | DYTN | 3321 | LOC338799 | 4789 | LPIN1 | 6257 | HMX1 | 7725 | KIR2DL4 |
| 386 | CENPH | 1854 | LOC401286 | 3322 | LOC100216546 | 4790 | RALA | 6258 | SEMA4D | 7726 | CRYBB3 |
| 387 | ZFPL1 | 1855 | LOC100131434 | 3323 | ANKRD30BL | 4791 | NFATC4 | 6259 | QTRT1 | 7727 | ZNF446 |
| 388 | PSMB9 | 1856 | KCNC3 | 3324 | NT5C2 | 4792 | CTD-2292M16.8 | 6260 | NUPR1 | 7728 | CNTLN |
| 389 | SARDH | 1857 | ACR | 3325 | KRT31 | 4793 | PLXNA3 | 6261 | AKR1E2 | 7729 | ZNF586 |
| 390 | DNASE2B | 1858 | LOC100134713 | 3326 | CORO6 | 4794 | DEPDC1B | 6262 | ANKS6 | 7730 | OR10H3 |
| 391 | PVRL2 | 1859 | ZNF266 | 3327 | RHBDD1 | 4795 | RIT1 | 6263 | SHC1 | 7731 | LCA10 |
| 392 | MPND | 1860 | ZFAT | 3328 | DUSP21 | 4796 | RFX4 | 6264 | SEC14L5 | 7732 | N4BP3 |
| 393 | AGTR1 | 1861 | KRTAP4-8 | 3329 | ARGFX | 4797 | MICALL1 | 6265 | COL8A1 | 7733 | TPP2 |
| 394 | PHF10 | 1862 | MIER1 | 3330 | FXYD5 | 4798 | HMCN2 | 6266 | CORO7-PAM16 | 7734 | STAP1 |
| 395 | PPP1CC | 1863 | OR5B17 | 3331 | TRIM78P | 4799 | SLCO4C1 | 6267 | FLJ46906 | 7735 | BACH2 |
| 396 | EDARADD | 1864 | WDR52 | 3332 | ANKRD43 | 4800 | VPRBP | 6268 | PTK2B | 7736 | SNN |
| 397 | PLIN2 | 1865 | TAF3 | 3333 | XKR6 | 4801 | AGAP2 | 6269 | DRG2 | 7737 | C9ORF68 |
| 398 | PPP2CA | 1866 | SCN9A | 3334 | LOC731275 | 4802 | C9ORF114 | 6270 | ZNF582-AS1 | 7738 | NDUFAF1 |
| 399 | KLHL2 | 1867 | ANKRD20A5P | 3335 | ANP32A-IT1 | 4803 | FAM84A | 6271 | KIAA0355 | 7739 | NAA38 |
| 400 | POLR2K | 1868 | KCTD3 | 3336 | SPRY3 | 4804 | PZP | 6272 | PPM1M | 7740 | MTMR7 |
| 401 | GNAS | 1869 | UPF2 | 3337 | MAN1B1 | 4805 | ANKHD1 | 6273 | ZSCAN12 | 7741 | IKBKAP |
| 402 | EEF2 | 1870 | BEGAIN | 3338 | PLP1 | 4806 | POLR2M | 6274 | ARHGEF10L | 7742 | TM6SF2 |
| 403 | FUNDC2 | 1871 | PSORS1C2 | 3339 | ARID5A | 4807 | HELLS | 6275 | MED26 | 7743 | C10ORF88 |
| 404 | NPC1 | 1872 | FSD1 | 3340 | MTAP | 4808 | RTN4IP1 | 6276 | LY86 | 7744 | N4BP2L2 |
| 405 | TM9SF2 | 1873 | ATP6AP1L | 3341 | INTS6 | 4809 | UPB1 | 6277 | RP11-30L8.1 | 7745 | KLHL30 |
| 406 | PLA2G4A | 1874 | ANKRD26P1 | 3342 | INTS4 | 4810 | DNMT3B | 6278 | EFCAB6 | 7746 | IL20RB |
| 407 | ZNF627 | 1875 | WDR86 | 3343 | C4ORF6 | 4811 | ADGB | 6279 | TKT | 7747 | HERPUD1 |
| 408 | CDIPT | 1876 | EFTUD1 | 3344 | PYY | 4812 | NCOR2 | 6280 | OPLAH | 7748 | HAUS1 |
| 409 | IDH2 | 1877 | ABCG8 | 3345 | DDI2 | 4813 | BIRC3 | 6281 | ZNF559 | 7749 | DYM |
| 410 | FAM118B | 1878 | LOC283788 | 3346 | AKR7L | 4814 | PLOD2 | 6282 | LINC00472 | 7750 | TTLL10 |
| 411 | FLVCR2 | 1879 | ANKRD13C | 3347 | BTNL8 | 4815 | BIN3 | 6283 | OASL | 7751 | FRS3 |
| 412 | CAPN1 | 1880 | IPO7 | 3348 | LOC100131662 | 4816 | TBCD | 6284 | LOC101928448 | 7752 | CTDSPL2 |
| 413 | RRAGD | 1881 | USP17 | 3349 | PRKG2 | 4817 | C3ORF65 | 6285 | BCAP31 | 7753 | C20ORF12 |
| 414 | PIGS | 1882 | TSSC1 | 3350 | POLR2J2 | 4818 | MFSD3 | 6286 | CCDC33 | 7754 | CYP7A1 |
| 415 | TES | 1883 | LOC100129763 | 3351 | ZNF578 | 4819 | C1ORF159 | 6287 | PADI6 | 7755 | SERPINA9 |
| 416 | THAP11 | 1884 | MYCL1 | 3352 | UNC13B | 4820 | SHISA5 | 6288 | PRR22 | 7756 | ZNF69 |
| 417 | AKR1B1 | 1885 | LOC100652763 | 3353 | MMP26 | 4821 | PORCN | 6289 | TP73 | 7757 | AQP7 |
| 418 | ALAD | 1886 | NKAPP1 | 3354 | COL20A1 | 4822 | MAP3K11 | 6290 | INTS12 | 7758 | VSX1 |
| 419 | RPL5 | 1887 | ABCA2 | 3355 | NRK | 4823 | TCF4 | 6291 | GZMA | 7759 | ZNF200 |
| 420 | ATP5A1 | 1888 | HSF1 | 3356 | PALM3 | 4824 | RPTOR | 6292 | PTDSS2 | 7760 | TIGD5 |
| 421 | ADORA2A | 1889 | DLG1 | 3357 | LOC91450 | 4825 | NUDT12 | 6293 | RANBP17 | 7761 | PDZD4 |
| 422 | PDGFD | 1890 | RRBP1 | 3358 | SART1 | 4826 | PEAK1 | 6294 | DDX20 | 7762 | IGF2AS |
| 423 | MFSD4 | 1891 | NFATC2IP | 3359 | KCNJ13 | 4827 | GALC | 6295 | ZNHIT3 | 7763 | HOXC13 |
| 424 | RMND5A | 1892 | SEPT6 | 3360 | DUSP9 | 4828 | ZNF827 | 6296 | BRD8 | 7764 | SPINK8 |
| 425 | TMBIM4 | 1893 | ZNF608 | 3361 | ZNF471 | 4829 | DSCAM | 6297 | WDR34 | 7765 | C19ORF62 |
| 426 | IER3IP1 | 1894 | ANKDD1A | 3362 | NOG | 4830 | KIAA1731 | 6298 | IPO13 | 7766 | FAM63B |
| 427 | TIMP4 | 1895 | LOC648691 | 3363 | WWC2 | 4831 | PLAU | 6299 | JPH4 | 7767 | IL27RA |
| 428 | RETSAT | 1896 | KIF12 | 3364 | REP15 | 4832 | PLEKHA5 | 6300 | ENDOU | 7768 | KDM2A |
| 429 | CLEC14A | 1897 | GATAD2B | 3365 | LOC340357 | 4833 | ITGA2 | 6301 | LAMC2 | 7769 | ZBTB25 |
| 430 | UCKL1 | 1898 | CA5B | 3366 | TP53AIP1 | 4834 | MED13L | 6302 | FLJ42200 | 7770 | KLHL11 |
| 431 | TP53BP2 | 1899 | LOC100130175 | 3367 | CASP3 | 4835 | TSPAN5 | 6303 | NFIB | 7771 | MDC1 |
| 432 | CCRL2 | 1900 | SLC44A5 | 3368 | ECEL1P2 | 4836 | RPP21 | 6304 | POLR3D | 7772 | NHEDC2 |
| 433 | SDF2 | 1901 | SCAF11 | 3369 | KRTAP4-11 | 4837 | CAPN13 | 6305 | C12ORF55 | 7773 | FBXW7 |
| 434 | RAPGEF1 | 1902 | SERP1 | 3370 | ZC3H13 | 4838 | ZNF140 | 6306 | PPT2 | 7774 | SIRPG |
| 435 | PTGS1 | 1903 | FAM48A | 3371 | DOK3 | 4839 | CABLES1 | 6307 | LOC729970 | 7775 | CAMK2A |
| 436 | C2ORF28 | 1904 | MAVS | 3372 | TMTC1 | 4840 | PAK6 | 6308 | CRHR1 | 7776 | SEL1L |
| 437 | KIAA1199 | 1905 | ZNF503 | 3373 | NTNG1 | 4841 | BNIPL | 6309 | FYTTD1 | 7777 | SECTM1 |
| 438 | SELS | 1906 | FLJ11292 | 3374 | PLEKHG4B | 4842 | NLRP2 | 6310 | DCP1B | 7778 | C14ORF37 |
| 439 | IDI1 | 1907 | IQCJ | 3375 | C7ORF51 | 4843 | ALG12 | 6311 | PLCE1 | 7779 | C6ORF62 |
| 440 | ACADS | 1908 | LOC100132593 | 3376 | LOC100128675 | 4844 | RALGAPA1 | 6312 | KCNC4 | 7780 | SHF |
| 441 | RAB32 | 1909 | C9ORF146 | 3377 | YBX2 | 4845 | SH3PXD2B | 6313 | SLC17A1 | 7781 | DEM1 |
| 442 | PISD | 1910 | FAM99A | 3378 | PLEKHH2 | 4846 | UBE4B | 6314 | HOXA-AS2 | 7782 | TBPL1 |
| 443 | LMAN2 | 1911 | MRPL42P5 | 3379 | LOC148696 | 4847 | MALT1 | 6315 | RBMS2 | 7783 | PRHOXNB |
| 444 | MTHFD2 | 1912 | SNAPC3 | 3380 | PFKFB3 | 4848 | IFT27 | 6316 | RNFT2 | 7784 | KRTAP24-1 |
| 445 | IDH3B | 1913 | RFPL4A | 3381 | LOC100131180 | 4849 | DGKD | 6317 | CNTN5 | 7785 | KRT23 |
| 446 | HM13 | 1914 | NAPB | 3382 | LOC100131234 | 4850 | ATP11B | 6318 | TOP1MT | 7786 | C12ORF34 |
| 447 | KLB | 1915 | CLCC1 | 3383 | YIPF3 | 4851 | NUP210L | 6319 | PAX8-AS1 | 7787 | TIMM22 |
| 448 | H2AFZ | 1916 | NTN1 | 3384 | C20ORF112 | 4852 | LRRK1 | 6320 | VARS2 | 7788 | ZNF883 |
| 449 | MCM9 | 1917 | ZNF517 | 3385 | RBAK | 4853 | KIF20A | 6321 | DUSP13 | 7789 | CISH |
| 450 | THBS2 | 1918 | MDGA1 | 3386 | DDRGK1 | 4854 | GGT5 | 6322 | DDO | 7790 | RBM8A |
| 451 | DOPEY2 | 1919 | PCDHGB2 | 3387 | LOC100128477 | 4855 | EIF3A | 6323 | EIF4ENIF1 | 7791 | SHC3 |
| 452 | ZKSCAN5 | 1920 | SEL1L2 | 3388 | LOC100130453 | 4856 | UBR4 | 6324 | COL9A1 | 7792 | BSG |
| 453 | ZNF207 | 1921 | PTRF | 3389 | PRRC2B | 4857 | KDM4C | 6325 | HEMK1 | 7793 | ADORA3 |
| 454 | PDCD10 | 1922 | CEP350 | 3390 | ABCA1 | 4858 | ERN1 | 6326 | TLDC1 | 7794 | TRNP1 |
| 455 | TRAPPC2P1 | 1923 | MYO1C | 3391 | KLRC4 | 4859 | NCAM2 | 6327 | HAUS3 | 7795 | LEO1 |
| 456 | ZNF434 | 1924 | C20ORF62 | 3392 | FLJ11710 | 4860 | NINL | 6328 | WDR44 | 7796 | ASCC3 |
| 457 | CHTF8 | 1925 | CXORF36 | 3393 | KLF17 | 4861 | TENM4 | 6329 | SYT5 | 7797 | XKR9 |
| 458 | PPIA | 1926 | CHST6 | 3394 | OR14C36 | 4862 | ZDHHC6 | 6330 | AKT1 | 7798 | CHD1 |
| 459 | MAGED1 | 1927 | ARL11 | 3395 | C7ORF54 | 4863 | STX1A | 6331 | CTBP1 | 7799 | AP1M1 |
| 460 | MAGEF1 | 1928 | SGK223 | 3396 | OR6K2 | 4864 | LRRFIP2 | 6332 | GALNT14 | 7800 | TGFB2 |
| 461 | LINC00244 | 1929 | FAM60A | 3397 | GVINP1 | 4865 | CSPP1 | 6333 | KRT35 | 7801 | CABP2 |
| 462 | NUDT15 | 1930 | PAGE3 | 3398 | ZNF577 | 4866 | LRP8 | 6334 | CFH | 7802 | RLBP1 |
| 463 | SAR1A | 1931 | FBXO17 | 3399 | LINC00410 | 4867 | AGAP3 | 6335 | CPE | 7803 | PKIB |
| 464 | DNAJB9 | 1932 | QRFPR | 3400 | ZNF718 | 4868 | RCOR3 | 6336 | LOC101928105 | 7804 | HSD11B1L |
| 465 | DCAF15 | 1933 | DHRS4L1 | 3401 | COQ4 | 4869 | ARR3 | 6337 | PHF5A | 7805 | PLEKHG6 |
| 466 | C6ORF48 | 1934 | DNAJC30 | 3402 | HERC2 | 4870 | ASXL1 | 6338 | PPP2R3A | 7806 | FBXO2 |
| 467 | GCM2 | 1935 | HERC2P2 | 3403 | COL4A6 | 4871 | CCDC65 | 6339 | THAP7 | 7807 | CDC7 |
| 468 | SELT | 1936 | LOC729706 | 3404 | LOC644145 | 4872 | HPS4 | 6340 | GLTSCR2 | 7808 | THSD7B |
| 469 | C20ORF30 | 1937 | LINC00482 | 3405 | KRTAP5-8 | 4873 | GPLD1 | 6341 | C12ORF80 | 7809 | TNFRSF10C |
| 470 | ETS2 | 1938 | CHN2 | 3406 | CALY | 4874 | KIAA1407 | 6342 | C9ORF43 | 7810 | MTFMT |
| 471 | SDF4 | 1939 | HIF3A | 3407 | GTPBP3 | 4875 | KIF21A | 6343 | RBM34 | 7811 | C9ORF171 |
| 472 | PRDX2 | 1940 | LOC100133130 | 3408 | POMZP3 | 4876 | MMP14 | 6344 | CPM | 7812 | SLC20A1 |
| 473 | ZMAT5 | 1941 | CCL14 | 3409 | PLCH2 | 4877 | KLHL21 | 6345 | FAM213B | 7813 | INPP5J |
| 474 | DLC1 | 1942 | CNOT1 | 3410 | LOC153684 | 4878 | HIF1A-AS2 | 6346 | LINC00263 | 7814 | RCCD1 |
| 475 | GTF2H4 | 1943 | KANK1 | 3411 | SCN8A | 4879 | C7ORF49 | 6347 | PGM2L1 | 7815 | TMEM217 |
| 476 | ID3 | 1944 | PIGM | 3412 | NF1P2 | 4880 | COPS7A | 6348 | NOP14-AS1 | 7816 | WBP11 |
| 477 | EXOSC5 | 1945 | C6ORF186 | 3413 | LOC254057 | 4881 | MVB12B | 6349 | PIK3C2A | 7817 | PCDHB6 |
| 478 | C8ORF48 | 1946 | LINC00265 | 3414 | WNT6 | 4882 | SH2D6 | 6350 | LRRC71 | 7818 | C16ORF70 |
| 479 | FAM96A | 1947 | LINC00162 | 3415 | ARSH | 4883 | RGPD4 | 6351 | SLC6A18 | 7819 | FBN2 |
| 480 | CCNYL1 | 1948 | CXORF68 | 3416 | DYRK1A | 4884 | SNX12 | 6352 | STK39 | 7820 | ZNF222 |
| 481 | TAX1BP3 | 1949 | ZNF542 | 3417 | RAB39B | 4885 | MIR29A | 6353 | ZUFSP | 7821 | NR2C2AP |
| 482 | HLA-DRB3 | 1950 | RNF170 | 3418 | PATE3 | 4886 | MAPK8IP3 | 6354 | ZNF37BP | 7822 | GAL3ST4 |
| 483 | PLA2G3 | 1951 | RAD51D | 3419 | ARL17A | 4887 | DBN1 | 6355 | CDK20 | 7823 | APOO |
| 484 | JAG2 | 1952 | GPR144 | 3420 | PDCD1LG2 | 4888 | MICAL2 | 6356 | NUTM2A | 7824 | MGAT3 |
| 485 | RND1 | 1953 | MEIS1 | 3421 | LINC00032 | 4889 | TDP1 | 6357 | ASIC1 | 7825 | ANKK1 |
| 486 | MCL1 | 1954 | CPLX3 | 3422 | C9ORF153 | 4890 | ELN | 6358 | ZNF10 | 7826 | MUL1 |
| 487 | NXT2 | 1955 | DCST2 | 3423 | ASAP3 | 4891 | TNFSF14 | 6359 | RAD21L1 | 7827 | VPS26B |
| 488 | ELF3 | 1956 | NKTR | 3424 | KLHL33 | 4892 | BRDT | 6360 | ACVR2A | 7828 | TMEM151B |
| 489 | TMEM111 | 1957 | PHKA1 | 3425 | HTR1B | 4893 | SEMA7A | 6361 | SEC16B | 7829 | TRIM8 |
| 490 | HDHD3 | 1958 | ZNF445 | 3426 | ZC3H11A | 4894 | IFT140 | 6362 | HPGDS | 7830 | SRRT |
| 491 | NUDCD1 | 1959 | BTN2A3P | 3427 | PCYT1B | 4895 | PABPN1L | 6363 | PDCD6IPP2 | 7831 | KIAA1377 |
| 492 | DARC | 1960 | SYNRG | 3428 | FOXP3 | 4896 | RNF213 | 6364 | SFTPC | 7832 | CCL7 |
| 493 | FNDC4 | 1961 | IGFL2 | 3429 | TSIX | 4897 | MTO1 | 6365 | ICK | 7833 | EIF6 |
| 494 | DUSP2 | 1962 | LOC150622 | 3430 | LOC100129662 | 4898 | ZNF384 | 6366 | BAI2 | 7834 | ZNF428 |
| 495 | PSAP | 1963 | NPHP3 | 3431 | N4BP2L1 | 4899 | ZDHHC17 | 6367 | AP1G2 | 7835 | C2CD4A |
| 496 | HLA-DPA1 | 1964 | HCN4 | 3432 | NADKD1 | 4900 | ARFGAP1 | 6368 | ALDH2 | 7836 | CAMK2D |
| 497 | TYRP1 | 1965 | OR5K1 | 3433 | KRTAP4-7 | 4901 | RP11-655C2.3 | 6369 | FBXO27 | 7837 | LCA5L |
| 498 | AP1B1 | 1966 | ATP13A2 | 3434 | KLRD1 | 4902 | LOC374443 | 6370 | KCNA3 | 7838 | CPT2 |
| 499 | C1QTNF1 | 1967 | CHD6 | 3435 | EDA2R | 4903 | DECR1 | 6371 | SCAMP3 | 7839 | RNF19B |
| 500 | RAB13 | 1968 | PKN2 | 3436 | LOC100126584 | 4904 | ACTL6A | 6372 | RAB30-AS1 | 7840 | DPY19L2 |
| 501 | ADAMTSL2 | 1969 | VPS13B | 3437 | C1ORF168 | 4905 | CCL13 | 6373 | DPF3 | 7841 | HSPC159 |
| 502 | ALKBH1 | 1970 | LOC100286979 | 3438 | RS1 | 4906 | SPOP | 6374 | LOC101928782 | 7842 | SYT13 |
| 503 | HPN | 1971 | C8ORF66 | 3439 | SOX6 | 4907 | MIB2 | 6375 | IER5 | 7843 | MIDN |
| 504 | PCGF3 | 1972 | ZNF462 | 3440 | UBE2DNL | 4908 | MAP3K13 | 6376 | ZNF215 | 7844 | WNT11 |
| 505 | LMF2 | 1973 | PLEC | 3441 | HLA-DOA | 4909 | PKD1L2 | 6377 | ARIH2 | 7845 | ZCCHC3 |
| 506 | ACOT7 | 1974 | MAGEE1 | 3442 | LUZP2 | 4910 | CCDC134 | 6378 | EDN2 | 7846 | FAM134B |
| 507 | TOMM70A | 1975 | CCDC165 | 3443 | HTR1F | 4911 | MIR4451 | 6379 | FLOT2 | 7847 | BCLAF1 |
| 508 | PHLDA2 | 1976 | GPR151 | 3444 | PARP10 | 4912 | ACOX2 | 6380 | GMPPA | 7848 | NIPAL4 |
| 509 | LOC727916 | 1977 | MDM4 | 3445 | TFF2 | 4913 | JUP | 6381 | PHYHIP | 7849 | URM1 |
| 510 | LEPR | 1978 | IL16 | 3446 | IMPA1 | 4914 | PIR-FIGF | 6382 | RP11-410L14.2 | 7850 | MPV17L2 |
| 511 | NUDT14 | 1979 | ENGASE | 3447 | LOC100132356 | 4915 | SLC43A3 | 6383 | CD8A | 7851 | MYOT |
| 512 | EEF1A1 | 1980 | C8ORF39 | 3448 | WNK1 | 4916 | CREB5 | 6384 | LINC00346 | 7852 | C14ORF102 |
| 513 | PSMB1 | 1981 | SH2D4B | 3449 | CEP44 | 4917 | SMTN | 6385 | LHX5 | 7853 | ODZ2 |
| 514 | NBN | 1982 | STK11 | 3450 | LOC100128184 | 4918 | IP6K2 | 6386 | TBX6 | 7854 | MFHAS1 |
| 515 | ARCN1 | 1983 | ZC3H3 | 3451 | RPL36AP33 | 4919 | B4GALT3 | 6387 | SGK494 | 7855 | DYDC2 |
| 516 | ASB8 | 1984 | AP1S3 | 3452 | ERBB2 | 4920 | DNAH6 | 6388 | SLC6A19 | 7856 | C18ORF22 |
| 517 | CENPE | 1985 | PCDHGB1 | 3453 | ATP10B | 4921 | ATG4B | 6389 | TMCC2 | 7857 | PPP2R2D |
| 518 | SMPD2 | 1986 | LOC100289200 | 3454 | LOC728061 | 4922 | TMC5 | 6390 | LST1 | 7858 | HPD |
| 519 | PDZRN3 | 1987 | ANGPTL1 | 3455 | B4GALNT3 | 4923 | MYOM3 | 6391 | KLK15 | 7859 | RBMXL2 |
| 520 | SLCO4A1 | 1988 | MIP | 3456 | EBLN2 | 4924 | CLDN11 | 6392 | SKAP1 | 7860 | KCNJ8 |
| 521 | CIAPIN1 | 1989 | SNX24 | 3457 | CEP104 | 4925 | PRICKLE4 | 6393 | PTX3 | 7861 | CCDC152 |
| 522 | HMGCLL1 | 1990 | RPAP2 | 3458 | PDE7A | 4926 | FER1L4 | 6394 | ASB4 | 7862 | VIT |
| 523 | KANSL3 | 1991 | LOC100132273 | 3459 | LOC149134 | 4927 | SMS | 6395 | LOC284930 | 7863 | GOLGA7B |
| 524 | DHX38 | 1992 | PKP1 | 3460 | FAM160A1 | 4928 | TSPAN3 | 6396 | ZNF638 | 7864 | PRM1 |
| 525 | AP1S2 | 1993 | FLJ14107 | 3461 | SCN7A | 4929 | CALCOCO1 | 6397 | FGD4 | 7865 | RSPO2 |
| 526 | SNX21 | 1994 | MASTL | 3462 | SLC25A27 | 4930 | CD72 | 6398 | SEC24B | 7866 | TRPV3 |
| 527 | RAB20 | 1995 | PDPR | 3463 | HEATR8 | 4931 | CELSR3 | 6399 | CASZ1 | 7867 | LRRC26 |
| 528 | BBIP1 | 1996 | USF2 | 3464 | C19ORF33 | 4932 | PCSK1 | 6400 | KBTBD7 | 7868 | GGCT |
| 529 | CCL3L3 | 1997 | TLE1 | 3465 | KLC4 | 4933 | ADD2 | 6401 | HEATR2 | 7869 | IL13 |
| 530 | IWS1 | 1998 | ABHD1 | 3466 | LOC100131150 | 4934 | KIR3DX1 | 6402 | SLC25A14 | 7870 | PAXIP1 |
| 531 | COL5A2 | 1999 | RALGDS | 3467 | SNAR-C3 | 4935 | RNU6-1003P | 6403 | MAG | 7871 | CENPV |
| 532 | ALAS1 | 2000 | C14ORF23 | 3468 | RND2 | 4936 | CYP27B1 | 6404 | MYT1L | 7872 | FAM155A |
| 533 | FUS | 2001 | SERPINA13 | 3469 | AMZ1 | 4937 | ABCA13 | 6405 | RGS9 | 7873 | SSTR2 |
| 534 | TSPAN9 | 2002 | IL26 | 3470 | USP42 | 4938 | PCIF1 | 6406 | LOC100130700 | 7874 | BATF3 |
| 535 | ASPH | 2003 | LOC100128554 | 3471 | PCDHGA5 | 4939 | GPS2 | 6407 | RP11-768G7.2 | 7875 | CLEC1A |
| 536 | BTF3P11 | 2004 | C6ORF170 | 3472 | LOC90246 | 4940 | SARAF | 6408 | CENPU | 7876 | NEK6 |
| 537 | DBNL | 2005 | OR52I2 | 3473 | FAM53B | 4941 | TIE1 | 6409 | AKR1C4 | 7877 | PPP1R1A |
| 538 | BRK1 | 2006 | CNTN6 | 3474 | LOC152024 | 4942 | CPSF3L | 6410 | BPHL | 7878 | PNPLA2 |
| 539 | GHR | 2007 | FZD9 | 3475 | DKFZP586B0319 | 4943 | GRHL2 | 6411 | NEU1 | 7879 | TIFA |
| 540 | COQ10B | 2008 | TNRC18 | 3476 | PCDHGB4 | 4944 | PTDSS1 | 6412 | EYA2 | 7880 | HRH1 |
| 541 | ZFAND2B | 2009 | SLC27A6 | 3477 | FAM201B | 4945 | LMNB1 | 6413 | PEBP4 | 7881 | KCNJ16 |
| 542 | AMPD2 | 2010 | GCN1L1 | 3478 | GDAP1 | 4946 | CELSR2 | 6414 | LAMTOR2 | 7882 | C11ORF93 |
| 543 | DIP2B | 2011 | ZNF836 | 3479 | CHRDL1 | 4947 | COL5A3 | 6415 | KLHL12 | 7883 | LYVE1 |
| 544 | DYRK3 | 2012 | HSD52 | 3480 | HIPK2 | 4948 | VILL | 6416 | LOC100289019 | 7884 | TMEM5 |
| 545 | MFAP3 | 2013 | PML | 3481 | COPS7B | 4949 | DIEXF | 6417 | GRIA3 | 7885 | ANGPTL5 |
| 546 | PTEN | 2014 | C8ORF77 | 3482 | ZCCHC11 | 4950 | PPIL2 | 6418 | ROBO3 | 7886 | LOC729991 |
| 547 | TFG | 2015 | LOC442421 | 3483 | NDP | 4951 | F2RL2 | 6419 | TCEA3 | 7887 | CHMP5 |
| 548 | CD200 | 2016 | MLL4 | 3484 | RBMY3AP | 4952 | SLIT1 | 6420 | SPAG16 | 7888 | GGCX |
| 549 | FXR2 | 2017 | CENPP | 3485 | ZNF248 | 4953 | DNMBP | 6421 | NOL6 | 7889 | NPTX2 |
| 550 | SRMS | 2018 | MON2 | 3486 | CD3G | 4954 | ZNF704 | 6422 | PTPN4 | 7890 | IFITM5 |
| 551 | SAMD4B | 2019 | CNST | 3487 | OGDHL | 4955 | NEB | 6423 | KIF28P | 7891 | C1QTNF3 |
| 552 | PPFIA3 | 2020 | TCF7 | 3488 | ZBTB1 | 4956 | TRPM7 | 6424 | SGMS2 | 7892 | CDKN2A |
| 553 | FAM175B | 2021 | LOC283075 | 3489 | HIP1R | 4957 | IFT122 | 6425 | RGSL1 | 7893 | OVCH2 |
| 554 | PARP3 | 2022 | HOXA10 | 3490 | C9ORF102 | 4958 | LINC00114 | 6426 | TMEM56 | 7894 | PLIN4 |
| 555 | KIF20B | 2023 | ZDHHC11 | 3491 | LOC729088 | 4959 | PIN1 | 6427 | ZNF474 | 7895 | PIK3R3 |
| 556 | ZNF513 | 2024 | LOC729626 | 3492 | CBWD7 | 4960 | SERPINB9 | 6428 | LIMA1 | 7896 | GSC2 |
| 557 | MED10 | 2025 | MARS | 3493 | LINC00511 | 4961 | TMEM19 | 6429 | FSD1L | 7897 | GABRB2 |
| 558 | ECE1 | 2026 | CHST9-AS1 | 3494 | LOC729324 | 4962 | ATAD2B | 6430 | KANK2 | 7898 | DCTN5 |
| 559 | RAP2C | 2027 | MCTS1 | 3495 | SPTAN1 | 4963 | CC2D1A | 6431 | ATP8A2 | 7899 | NDUFV3 |
| 560 | LPCAT1 | 2028 | LOC283731 | 3496 | ASAP1-IT1 | 4964 | TP53I13 | 6432 | HAUS7 | 7900 | MPI |
| 561 | ZBTB9 | 2029 | CHM | 3497 | C8ORF75 | 4965 | USP49 | 6433 | PHC2 | 7901 | DNM1L |
| 562 | TSNAX | 2030 | TRMT2B | 3498 | LOC284939 | 4966 | ABLIM2 | 6434 | INTS9 | 7902 | DDX11 |
| 563 | LMO3 | 2031 | LOC349196 | 3499 | TBC1D28 | 4967 | EPM2A | 6435 | RAD9A | 7903 | DDIT4 |
| 564 | SH3BP5 | 2032 | RPL23AP32 | 3500 | FRMD3 | 4968 | RECK | 6436 | LOC100507330 | 7904 | IRF1 |
| 565 | OSGIN1 | 2033 | COBL | 3501 | GOLGB1 | 4969 | MLIP | 6437 | HAL | 7905 | GPR142 |
| 566 | GJA1 | 2034 | AAK1 | 3502 | LOC100129148 | 4970 | TTLL3 | 6438 | MROH5 | 7906 | AGAP1 |
| 567 | C2ORF43 | 2035 | ZNF818P | 3503 | SLC17A4 | 4971 | SLC25A42 | 6439 | LANCL2 | 7907 | FBXO11 |
| 568 | ARMC9 | 2036 | LOC100127946 | 3504 | GML | 4972 | WFDC21P | 6440 | LOC101926960 | 7908 | ISM1 |
| 569 | ANKRD10 | 2037 | LIMS2 | 3505 | LOC284630 | 4973 | RP11-196E1.3 | 6441 | LOC101928834 | 7909 | CYP27C1 |
| 570 | LOC100130430 | 2038 | RGPD5 | 3506 | VAPB | 4974 | FAM198B | 6442 | TSNAXIP1 | 7910 | SP1 |
| 571 | SYNM | 2039 | PPM1B | 3507 | FAM21C | 4975 | FAM20A | 6443 | SLC38A10 | 7911 | FAM155B |
| 572 | CUL2 | 2040 | C17ORF76 | 3508 | ABP1 | 4976 | DTL | 6444 | RP1-213J1P__B.1 | 7912 | POP5 |
| 573 | DPAGT1 | 2041 | SLC9A3R2 | 3509 | BTN3A1 | 4977 | TADA2A | 6445 | DBX1 | 7913 | PPM1L |
| 574 | PARP9 | 2042 | LCN9 | 3510 | TAF1B | 4978 | HMGXB3 | 6446 | E2F8 | 7914 | PTPN5 |
| 575 | DNAJB2 | 2043 | POLR1C | 3511 | LOC254100 | 4979 | COL12A1 | 6447 | GLP2R | 7915 | PI16 |
| 576 | GLT25D1 | 2044 | CFL1P1 | 3512 | PRSS47 | 4980 | G2E3 | 6448 | CAMK4 | 7916 | ADIPOQ |
| 577 | TNFRSF18 | 2045 | PRLH | 3513 | ODF3 | 4981 | DMKN | 6449 | NPRL2 | 7917 | CDH12 |
| 578 | ZFP57 | 2046 | ZNF208 | 3514 | CIZ1 | 4982 | FRA10AC1 | 6450 | C5ORF24 | 7918 | IGFBP6 |
| 579 | C19ORF54 | 2047 | FAM25C | 3515 | CLASP1 | 4983 | LRP6 | 6451 | ZC3HC1 | 7919 | FAM24A |
| 580 | ERGIC3 | 2048 | MYZAP | 3516 | HCRT | 4984 | AMELX | 6452 | DMPK | 7920 | SAA1 |
| 581 | DPH3 | 2049 | MPRIP | 3517 | LOC100131829 | 4985 | MRPS6 | 6453 | SLCO2B1 | 7921 | TDRD10 |
| 582 | FERMT3 | 2050 | LINC00158 | 3518 | CYP4F11 | 4986 | SNRNP27 | 6454 | PFKM | 7922 | INHBB |
| 583 | CHST14 | 2051 | LOC100653296 | 3519 | LOC100289333 | 4987 | SLCO5A1 | 6455 | ACVRL1 | 7923 | GRRP1 |
| 584 | MAOB | 2052 | OR10AG1 | 3520 | OR6C4 | 4988 | ODF2 | 6456 | SAMD12 | 7924 | GCC2 |
| 585 | LOC100133050 | 2053 | YIF1B | 3521 | CNTNAP3 | 4989 | AC108025.2 | 6457 | IGIP | 7925 | FCGR3A |
| 586 | AGFG1 | 2054 | INTU | 3522 | RNF215 | 4990 | C10ORF54 | 6458 | DUSP6 | 7926 | BUB3 |
| 587 | ADAMTS9 | 2055 | ERBB3 | 3523 | LOC442366 | 4991 | PLEKHO1 | 6459 | PTN | 7927 | SKP2 |
| 588 | ATP5B | 2056 | RUFY2 | 3524 | TRPM6 | 4992 | BAIAP2 | 6460 | BAG2 | 7928 | CTNNB1 |
| 589 | COL6A2 | 2057 | MGAT5B | 3525 | SLMAP | 4993 | D2HGDH | 6461 | TEAD3 | 7929 | RBMXL1 |
| 590 | LSM10 | 2058 | ZNF192 | 3526 | CXORF51A | 4994 | NELL1 | 6462 | KCTD21-AS1 | 7930 | FAM220A |
| 591 | RPS21 | 2059 | FRG2C | 3527 | CNTRL | 4995 | WDR73 | 6463 | ACOT8 | 7931 | HNRNPCL2 |
| 592 | TMEM184C | 2060 | GRM8 | 3528 | S100Z | 4996 | POMT2 | 6464 | MORN2 | 7932 | RAP1B |
| 593 | COPE | 2061 | ZNF235 | 3529 | SNORA62 | 4997 | ALCAM | 6465 | STAB2 | 7933 | BPGM |
| 594 | C17ORF101 | 2062 | CACNG4 | 3530 | MAP3K4 | 4998 | SUPT20H | 6466 | NDUFS3 | 7934 | TOP1P1 |
| 595 | GABARAPL2 | 2063 | OCLN | 3531 | KRTAP5-5 | 4999 | PIAS3 | 6467 | LINC01410 | 7935 | CCT6P1 |
| 596 | HSP90AB2P | 2064 | LOC100506390 | 3532 | ZNFX1 | 5000 | ARHGAP31 | 6468 | CDHR5 | 7936 | P2RY14 |
| 597 | CHRM4 | 2065 | LENG9 | 3533 | REG1P | 5001 | ATP1A1-AS1 | 6469 | LINC00452 | 7937 | BOD1L1 |
| 598 | CTSL1 | 2066 | MPP3 | 3534 | C14ORF82 | 5002 | KIT | 6470 | HNF4A | 7938 | NNT |
| 599 | PPA1 | 2067 | ZSCAN30 | 3535 | AQP8 | 5003 | ETNPPL | 6471 | BEND2 | 7939 | ARL13B |
| 600 | WDR36 | 2068 | LOC285389 | 3536 | LETM1 | 5004 | CNGB1 | 6472 | FOXK1 | 7940 | ZNF124 |
| 601 | SIX4 | 2069 | TLR8-AS1 | 3537 | MGA | 5005 | DRC1 | 6473 | ALOX12B | 7941 | VEZT |
| 602 | C6ORF211 | 2070 | PSITPTE22 | 3538 | PHLPP2 | 5006 | GSTM2 | 6474 | COL26A1 | 7942 | LUC7L3 |
| 603 | RPS2P32 | 2071 | LMTK3 | 3539 | PKDCC | 5007 | ZFYVE20 | 6475 | MKNK2 | 7943 | GTF2H2 |
| 604 | CD2BP2 | 2072 | SPATA3 | 3540 | KRTAP5-7 | 5008 | HGFAC | 6476 | RP11-521M14.2 | 7944 | STX7 |
| 605 | ZNF185 | 2073 | KIR2DS4 | 3541 | BAGE | 5009 | TXK | 6477 | KIF14 | 7945 | ESM1 |
| 606 | CHCHD3 | 2074 | DGCR8 | 3542 | DGKE | 5010 | ACP6 | 6478 | NDUFAB1 | 7946 | ING3 |
| 607 | ZDHHC12 | 2075 | MYEOV | 3543 | SENP7 | 5011 | ESPL1 | 6479 | SLC34A2 | 7947 | RBMS1 |
| 608 | WNT7B | 2076 | APC | 3544 | STAT4 | 5012 | ZNF491 | 6480 | PRKAR2B | 7948 | C1ORF27 |
| 609 | G6PD | 2077 | SLC18A3 | 3545 | ZNF488 | 5013 | DLL4 | 6481 | CLNS1A | 7949 | LY75 |
| 610 | C2ORF18 | 2078 | PRPS2 | 3546 | SPACA4 | 5014 | MTHFR | 6482 | RHOQ | 7950 | BCHE |
| 611 | EIF2S2 | 2079 | LOC100505933 | 3547 | SNX29 | 5015 | TMPRSS3 | 6483 | VWA8 | 7951 | TOPORS |
| 612 | C2 | 2080 | TAS2R5 | 3548 | MYH14 | 5016 | HIRA | 6484 | BFSP2 | 7952 | SOCS4 |
| 613 | HRAS | 2081 | DOCK5 | 3549 | CENPO | 5017 | MMP7 | 6485 | GNA11 | 7953 | TSPAN2 |
| 614 | PCK2 | 2082 | PPP1R8 | 3550 | SLC9A4 | 5018 | ALS2CL | 6486 | COL7A1 | 7954 | MPHOSPH10 |
| 615 | ATG13 | 2083 | MTMR14 | 3551 | GAPVD1 | 5019 | ERAL1 | 6487 | ZBTB47 | 7955 | FNDC1 |
| 616 | TFRC | 2084 | ZSCAN1 | 3552 | C5ORF56 | 5020 | AK5 | 6488 | AIPL1 | 7956 | SNRPE |
| 617 | SIRT3 | 2085 | KDM6A | 3553 | TIAL1 | 5021 | MFSD12 | 6489 | EXOC6B | 7957 | NUDT21 |
| 618 | C12ORF44 | 2086 | KLK6 | 3554 | ATXN3 | 5022 | C9ORF142 | 6490 | MSX1 | 7958 | ACOT2 |
| 619 | METTL11A | 2087 | OR4F15 | 3555 | SEC61A2 | 5023 | ZCCHC8 | 6491 | TERT | 7959 | NAP1L1 |
| 620 | YES1 | 2088 | KAT6A | 3556 | NHSL2 | 5024 | MLXIPL | 6492 | ENTPD8 | 7960 | CPEB3 |
| 621 | OXA1L | 2089 | PURG | 3557 | LINC00302 | 5025 | RBM45 | 6493 | C14ORF166 | 7961 | SEPT3 |
| 622 | HSD17B7 | 2090 | TRAF2 | 3558 | SNORA74B | 5026 | CYFIP1 | 6494 | RADIL | 7962 | MLX |
| 623 | EIF2B4 | 2091 | HSD17B3 | 3559 | OR8B4 | 5027 | DHTKD1 | 6495 | ART1 | 7963 | ELOVL7 |
| 624 | TIMP1 | 2092 | GLI4 | 3560 | SYDE2 | 5028 | BUB1 | 6496 | KAZN | 7964 | CCDC82 |
| 625 | CDCP1 | 2093 | ACTB | 3561 | LOC285629 | 5029 | CACNA1C | 6497 | RP11-131H24.4 | 7965 | KIF1BP |
| 626 | AIFM3 | 2094 | PYGO1 | 3562 | DDX17 | 5030 | CPQ | 6498 | CCDC157 | 7966 | PPP1R15B |
| 627 | CRMP1 | 2095 | LHB | 3563 | SEPT12 | 5031 | ZNF341 | 6499 | AAMDC | 7967 | HNRNPH1 |
| 628 | WBP5 | 2096 | UBQLNL | 3564 | TMEM146 | 5032 | GPR68 | 6500 | TNIP3 | 7968 | CTAGE6 |
| 629 | MLST8 | 2097 | GHRL | 3565 | BTN2A1 | 5033 | TTYH3 | 6501 | POLQ | 7969 | NDUFA5 |
| 630 | HNRNPA2B1 | 2098 | AIFM2 | 3566 | DND1 | 5034 | SLC13A3 | 6502 | MINK1 | 7970 | ARL4A |
| 631 | TMEM85 | 2099 | BCL9 | 3567 | GRM5 | 5035 | SNX14 | 6503 | PHKA2 | 7971 | SMAD5 |
| 632 | PPIAL4A | 2100 | ANKRD18A | 3568 | SULT1C4 | 5036 | PAX3 | 6504 | SMYD4 | 7972 | FAM229B |
| 633 | CTSA | 2101 | KRTAP10-8 | 3569 | RSC1A1 | 5037 | OTUD4 | 6505 | FFAR4 | 7973 | TMA16 |
| 634 | HSPA2 | 2102 | SPPL2B | 3570 | RREB1 | 5038 | TRAF3 | 6506 | NDUFB9 | 7974 | MAP9 |
| 635 | LAPTM5 | 2103 | LATS1 | 3571 | CLEC17A | 5039 | DECR2 | 6507 | ZNF671 | 7975 | ANXA2P1 |
| 636 | ARSE | 2104 | RALB | 3572 | MGC16142 | 5040 | C12ORF29 | 6508 | TNN | 7976 | ALG13 |
| 637 | TYMS | 2105 | PCDH11Y | 3573 | ESYT3 | 5041 | PRKCI | 6509 | MYO6 | 7977 | SRSF3 |
| 638 | B3GNT9 | 2106 | MOG | 3574 | ZNF646 | 5042 | PTGDS | 6510 | CMTR1 | 7978 | MIR130A |
| 639 | SAMSN1 | 2107 | ZNF81 | 3575 | ARHGEF35 | 5043 | CRB1 | 6511 | C11ORF74 | 7979 | POSTN |
| 640 | FAH | 2108 | MAGEB5 | 3576 | SLC26A7 | 5044 | ZNF559-ZNF177 | 6512 | DNAH2 | 7980 | TPRKB |
| 641 | EMG1 | 2109 | SSC5D | 3577 | KIAA1841 | 5045 | NCAPG2 | 6513 | USH2A | 7981 | SYF2 |
| 642 | CDC123 | 2110 | PLA2G6 | 3578 | LOC100127940 | 5046 | ALDOB | 6514 | PPM1N | 7982 | PLA2G12A |
| 643 | RPL15 | 2111 | ZNF382 | 3579 | LOC100134409 | 5047 | CD59 | 6515 | RPL3L | 7983 | CXORF21 |
| 644 | LRRC16A | 2112 | TECPR2 | 3580 | FLJ37201 | 5048 | LOC102724094 | 6516 | RAB14 | 7984 | SLC25A32 |
| 645 | MAGEL2 | 2113 | LOC100128498 | 3581 | GPR35 | 5049 | KDM7A | 6517 | CACNA1D | 7985 | C4ORF32 |
| 646 | MRPL43 | 2114 | ANKRD11 | 3582 | LOC284215 | 5050 | AASDH | 6518 | C8ORF82 | 7986 | RPEL1 |
| 647 | TRPC4AP | 2115 | HDGF | 3583 | ASH1L | 5051 | ABCC1 | 6519 | FUZ | 7987 | PPP1CB |
| 648 | RPSA | 2116 | PDZD7 | 3584 | ZBTB40 | 5052 | GJB2 | 6520 | RP11-108O10.2 | 7988 | EEF1B2 |
| 649 | PITHD1 | 2117 | NARG2 | 3585 | IL4R | 5053 | FAM160A2 | 6521 | GGA3 | 7989 | KATNBL1 |
| 650 | PFN2 | 2118 | SCARNA9 | 3586 | FEZF2 | 5054 | AVIL | 6522 | IMPDH1 | 7990 | TUBA3D |
| 651 | KIAA0319L | 2119 | RFX3 | 3587 | PLEKHM3 | 5055 | RASIP1 | 6523 | DDX56 | 7991 | EPG5 |
| 652 | KRT18 | 2120 | ZNF703 | 3588 | PCA3 | 5056 | KIAA0825 | 6524 | ADRBK1 | 7992 | TCAF2 |
| 653 | LOC440292 | 2121 | VASP | 3589 | HYAL4 | 5057 | CBY1 | 6525 | MYPN | 7993 | RBBP4 |
| 654 | COL6A3 | 2122 | LOC340335 | 3590 | SLC24A1 | 5058 | MECR | 6526 | FAM120C | 7994 | SENP6 |
| 655 | TEX264 | 2123 | RAB2B | 3591 | NKX6-2 | 5059 | OTOGL | 6527 | DLEU7 | 7995 | PTPN2 |
| 656 | SLC6A12 | 2124 | GTF2I | 3592 | PRPF40A | 5060 | ARMC3 | 6528 | TMEM45A | 7996 | LSM5 |
| 657 | SNCA | 2125 | APTX | 3593 | PRPF18 | 5061 | M6PR | 6529 | CHRND | 7997 | SNX7 |
| 658 | PITPNM1 | 2126 | JAK3 | 3594 | FBXL7 | 5062 | RHOU | 6530 | TINF2 | 7998 | UTP23 |
| 659 | RPL28 | 2127 | CRYGD | 3595 | LOC100131820 | 5063 | GPR124 | 6531 | BCL6B | 7999 | ABL1 |
| 660 | TACO1 | 2128 | C10ORF112 | 3596 | C16ORF3 | 5064 | DGKZ | 6532 | FRMPD4 | 8000 | DUSP26 |
| 661 | TAF5 | 2129 | SLC9A5 | 3597 | MSL1 | 5065 | GDI1 | 6533 | ITGAL | 8001 | APH1A |
| 662 | LAPTM4B | 2130 | AIF1L | 3598 | KRTAP10-9 | 5066 | HDLBP | 6534 | IL1A | 8002 | SNORD87 |
| 663 | MAN2B2 | 2131 | ZNF740 | 3599 | MS4A15 | 5067 | ADAMTS6 | 6535 | CSPG4 | 8003 | ORC5 |
| 664 | BHLHE40 | 2132 | NFYA | 3600 | APP | 5068 | PRKG1-AS1 | 6536 | RRP12 | 8004 | ANP32C |
| 665 | SLC35B1 | 2133 | YEATS2 | 3601 | PROX2 | 5069 | JAG1 | 6537 | TMCO4 | 8005 | SLC9A6 |
| 666 | C2CD2 | 2134 | STK3 | 3602 | FAM40A | 5070 | GRK6 | 6538 | SGOL1 | 8006 | PI4K2B |
| 667 | STEAP2 | 2135 | FAM122B | 3603 | TMEM211 | 5071 | LOC400499 | 6539 | TLX3 | 8007 | ERP44 |
| 668 | ZNF281 | 2136 | JRK | 3604 | LOC283575 | 5072 | FABP3 | 6540 | PVR | 8008 | PIAS2 |
| 669 | MOB4 | 2137 | ATG16L2 | 3605 | PHF21B | 5073 | PRR5-ARHGAP8 | 6541 | TAF4 | 8009 | SUCO |
| 670 | PRPS1 | 2138 | ZC3HAV1L | 3606 | C6ORF204 | 5074 | WSB2 | 6542 | MCAM | 8010 | EIF1AD |
| 671 | VAX1 | 2139 | ND4L | 3607 | VEGFA | 5075 | RELB | 6543 | C5ORF60 | 8011 | TAF4B |
| 672 | MEST | 2140 | GBP2 | 3608 | PIH1D2 | 5076 | HIF1A-AS1 | 6544 | LOC100506258 | 8012 | ANP32A |
| 673 | LOC100507547 | 2141 | LOC284260 | 3609 | C12ORF33 | 5077 | TMEM262 | 6545 | SEMA4A | 8013 | POTEE |
| 674 | BCL11A | 2142 | PNPLA7 | 3610 | FLJ44342 | 5078 | PTPN21 | 6546 | SLC6A1 | 8014 | MED21 |
| 675 | FAM135A | 2143 | FLJ41327 | 3611 | PABPC5 | 5079 | NDRG1 | 6547 | LRP11 | 8015 | PTGR2 |
| 676 | POLR3F | 2144 | GNAO1 | 3612 | SV2C | 5080 | MAST2 | 6548 | SLC46A1 | 8016 | SP110 |
| 677 | FADS3 | 2145 | BMS1 | 3613 | IL20RA | 5081 | NPHP4 | 6549 | CERS3 | 8017 | NAA25 |
| 678 | RHOV | 2146 | OR2A7 | 3614 | C3ORF35 | 5082 | EHHADH | 6550 | RPH3AL | 8018 | PSG9 |
| 679 | SDHC | 2147 | ATAD5 | 3615 | THAP7-AS1 | 5083 | BMP6 | 6551 | RBM17 | 8019 | KPNA2 |
| 680 | STOM | 2148 | IMMT | 3616 | PPIH | 5084 | CDCA3 | 6552 | DEFB133 | 8020 | SERINC3 |
| 681 | OVOL1 | 2149 | ZNF551 | 3617 | FOXA3 | 5085 | POMGNT1 | 6553 | TRPM1 | 8021 | MIGA1 |
| 682 | SMG9 | 2150 | LOC100133161 | 3618 | DENND1A | 5086 | SETD6 | 6554 | CLSPN | 8022 | PPIL3 |
| 683 | CHSY3 | 2151 | LINC00113 | 3619 | LOC100128402 | 5087 | RP11-114G22.1 | 6555 | PRDM15 | 8023 | RANBP6 |
| 684 | PPFIA1 | 2152 | EFCAB8 | 3620 | TJP2 | 5088 | PKP3 | 6556 | LOC101927257 | 8024 | UBE2W |
| 685 | PRRC2C | 2153 | TOMM20L | 3621 | CFLAR | 5089 | LINC00461 | 6557 | UNC80 | 8025 | SNORA32 |
| 686 | PYDC1 | 2154 | OR6B3 | 3622 | SOX13 | 5090 | MCCC2 | 6558 | LGR4 | 8026 | UQCRH |
| 687 | TBL3 | 2155 | NSF | 3623 | RABGAP1 | 5091 | GPX8 | 6559 | CFD | 8027 | DDHD1 |
| 688 | SMG1 | 2156 | PTPRC | 3624 | SLAMF6 | 5092 | FANCI | 6560 | C15ORF40 | 8028 | DYNC1LI1 |
| 689 | ENO1 | 2157 | BCAS3 | 3625 | DIO3OS | 5093 | MON1A | 6561 | CCRN4L | 8029 | AK6 |
| 690 | RCAN1 | 2158 | ZMYM6 | 3626 | C14ORF41 | 5094 | C17ORF70 | 6562 | ACYP2 | 8030 | SUMO1 |
| 691 | CDCA7 | 2159 | C1ORF226 | 3627 | DMTF1 | 5095 | TP53INP1 | 6563 | PKHD1L1 | 8031 | CEP63 |
| 692 | GUK1 | 2160 | LOC728147 | 3628 | SLC19A3 | 5096 | UCHL1 | 6564 | PCYT2 | 8032 | SPHAR |
| 693 | 15-Sep | 2161 | PDCL2 | 3629 | TGM7 | 5097 | UBXN7 | 6565 | PDGFRB | 8033 | ERI1 |
| 694 | PIP5K1B | 2162 | LSM14B | 3630 | HEATR7A | 5098 | CTNNAL1 | 6566 | CACNA2D2 | 8034 | SCARA3 |
| 695 | MAPK10 | 2163 | SNORA73A | 3631 | LOC151009 | 5099 | FAM193A | 6567 | RTCA | 8035 | KLF5 |
| 696 | PQBP1 | 2164 | ZSWIM6 | 3632 | DUX4L4 | 5100 | MUC2 | 6568 | ABCC9 | 8036 | DCTN6 |
| 697 | EAF1 | 2165 | LINC00092 | 3633 | PHACTR1 | 5101 | GGT3P | 6569 | PLA1A | 8037 | COMMD8 |
| 698 | SLAIN1 | 2166 | ZSCAN23 | 3634 | LOC100131231 | 5102 | DNHD1 | 6570 | RNF157 | 8038 | FAM76A |
| 699 | TULP1 | 2167 | LOC730338 | 3635 | DNMT3A | 5103 | LRP5 | 6571 | FAM86C2P | 8039 | RANBP9 |
| 700 | SLBP | 2168 | WDFY4 | 3636 | TSNARE1 | 5104 | RP11-484O2.1 | 6572 | TEKT4 | 8040 | HERPUD2 |
| 701 | C10ORF46 | 2169 | DPRX | 3637 | LOC100128563 | 5105 | VSTM4 | 6573 | RP11-1143G9.4 | 8041 | SLC4A4 |
| 702 | RRP1 | 2170 | C5ORF63 | 3638 | C5ORF25 | 5106 | CASK | 6574 | VWA7 | 8042 | PIK3R1 |
| 703 | HLA-DQB2 | 2171 | ZNF563 | 3639 | LOC100129888 | 5107 | GATA1 | 6575 | LOC100505592 | 8043 | ZNF735 |
| 704 | PEA15 | 2172 | OR6B2 | 3640 | SPATA9 | 5108 | POLR2G | 6576 | FAM161A | 8044 | ZNF85 |
| 705 | ESR1 | 2173 | NAALADL2 | 3641 | C10ORF129 | 5109 | MED13 | 6577 | CDYL | 8045 | HIKESHI |
| 706 | TMED9 | 2174 | PIDD | 3642 | MB | 5110 | PDE4A | 6578 | HNF1A | 8046 | LIG4 |
| 707 | ADPGK | 2175 | HSPG2 | 3643 | LOC643441 | 5111 | ABHD6 | 6579 | MPP6 | 8047 | TP53TG1 |
| 708 | SRM | 2176 | FLJ13773 | 3644 | GOLGA6L6 | 5112 | GPRASP1 | 6580 | PREB | 8048 | ITM2A |
| 709 | TJP3 | 2177 | CDK13 | 3645 | SETD3 | 5113 | UBE3B | 6581 | FLRT3 | 8049 | ADI1 |
| 710 | FAM83D | 2178 | RPS6KA2 | 3646 | PKD1L3 | 5114 | ABLIM1 | 6582 | DXO | 8050 | NCOA1 |
| 711 | TFPI | 2179 | EFNA2 | 3647 | TAS2R4 | 5115 | PAPD5 | 6583 | SHQ1 | 8051 | CNEP1R1 |
| 712 | SH3TC1 | 2180 | LPAR4 | 3648 | FLJ21369 | 5116 | PIP4K2C | 6584 | NFIA | 8052 | TDP2 |
| 713 | ADCY6 | 2181 | GTF2IRD2 | 3649 | LOC100129373 | 5117 | ZFYVE1 | 6585 | ARHGEF19 | 8053 | MCFD2 |
| 714 | EXOC2 | 2182 | ZNF192P1 | 3650 | IKZF1 | 5118 | P2RY6 | 6586 | TRIM39-RPP21 | 8054 | OTUD6B-AS1 |
| 715 | KIF18A | 2183 | LOC648149 | 3651 | GSX1 | 5119 | C18ORF63 | 6587 | SYTL1 | 8055 | EAPP |
| 716 | CPVL | 2184 | LOC729020 | 3652 | LOC284581 | 5120 | ILDR2 | 6588 | PLCB2 | 8056 | GIMAP7 |
| 717 | ASF1A | 2185 | FAR1 | 3653 | ODF2L | 5121 | EDNRB | 6589 | LINC00982 | 8057 | MFSD14A |
| 718 | HPSE | 2186 | NEIL3 | 3654 | C9ORF47 | 5122 | CDC25A | 6590 | EML6 | 8058 | PPIG |
| 719 | MTHFD1L | 2187 | KRT33A | 3655 | GGN | 5123 | STOX2 | 6591 | PLEKHA4 | 8059 | HACD4 |
| 720 | LOC389023 | 2188 | RBM15 | 3656 | LOC100128670 | 5124 | HELZ2 | 6592 | AURKB | 8060 | GGH |
| 721 | CAPN12 | 2189 | CDCP2 | 3657 | RAB19 | 5125 | ARFGAP2 | 6593 | MBNL2 | 8061 | TRMT6 |
| 722 | RAPGEF3 | 2190 | USP17L2 | 3658 | TAB2 | 5126 | SLC35D1 | 6594 | INPP5E | 8062 | SNAP23 |
| 723 | MID1 | 2191 | LOC100129572 | 3659 | MGC21881 | 5127 | AACSP1 | 6595 | COMMD2 | 8063 | DNAJC2 |
| 724 | ZW10 | 2192 | LOC200830 | 3660 | ZSWIM4 | 5128 | PPIF | 6596 | MPZL2 | 8064 | DUSP11 |
| 725 | VPS39 | 2193 | SVEP1 | 3661 | KISS1 | 5129 | CCDC88B | 6597 | CCT6B | 8065 | MRPL3 |
| 726 | SAT1 | 2194 | CBX5 | 3662 | TRAPPC10 | 5130 | DNAJB12 | 6598 | TENM2 | 8066 | VPS26A |
| 727 | RUVBL2 | 2195 | SLC35B4 | 3663 | SNORA23 | 5131 | TM4SF19 | 6599 | CADPS2 | 8067 | C2ORF69 |
| 728 | HTATIP2 | 2196 | EXD2 | 3664 | MLLT6 | 5132 | EMB | 6600 | JPH2 | 8068 | RIDA |
| 729 | GALNT2 | 2197 | CHRM2 | 3665 | NCAN | 5133 | BAIAP2L2 | 6601 | RMDN3 | 8069 | KAT2B |
| 730 | UQCR10 | 2198 | EIF4G3 | 3666 | GRIK2 | 5134 | RUSC2 | 6602 | UNC45B | 8070 | MED7 |
| 731 | MPHOSPH6 | 2199 | LOC100130950 | 3667 | C21ORF88 | 5135 | PLA2G4C | 6603 | POU2F1 | 8071 | EIF1AX |
| 732 | KCNE3 | 2200 | UNKL | 3668 | VPS37A | 5136 | CYFIP2 | 6604 | VWA3B | 8072 | MRPL47 |
| 733 | SMAD2 | 2201 | FOXP2 | 3669 | LOC100128333 | 5137 | MNX1 | 6605 | KIF9 | 8073 | LINC00526 |
| 734 | BOD1L | 2202 | SLC24A4 | 3670 | POLE | 5138 | TIAM2 | 6606 | LOC101928190 | 8074 | BZW1 |
| 735 | SUN2 | 2203 | CHMP1A | 3671 | BCAR4 | 5139 | UBASH3B | 6607 | DKK4 | 8075 | IRF2 |
| 736 | ITGB1BP1 | 2204 | LOC649201 | 3672 | JPX | 5140 | CNKSR2 | 6608 | RHOBTB1 | 8076 | LSM6 |
| 737 | HMGCS1 | 2205 | PIK3C2B | 3673 | CYP11B1 | 5141 | GHSR | 6609 | RP11-342M1.3 | 8077 | ZFP36L1 |
| 738 | SNHG13 | 2206 | YPEL4 | 3674 | LOC100127909 | 5142 | RAPGEF5 | 6610 | BTNL2 | 8078 | PLAGL1 |
| 739 | PHLPP1 | 2207 | TMEM194B | 3675 | HIST1H2BA | 5143 | BASP1 | 6611 | KIAA1279 | 8079 | RAB33B |
| 740 | COMMD4 | 2208 | FLJ36000 | 3676 | MAMLD1 | 5144 | STXBP5L | 6612 | RASL11A | 8080 | TAF1D |
| 741 | HERC5 | 2209 | POM121L1P | 3677 | OBP2B | 5145 | PDLIM4 | 6613 | KIAA1107 | 8081 | UGP2 |
| 742 | DCUN1D4 | 2210 | MYCNOS | 3678 | LOC100130581 | 5146 | MRPL2 | 6614 | RPL6 | 8082 | NDUFAF4 |
| 743 | NMNAT3 | 2211 | LOC399753 | 3679 | C9ORF4 | 5147 | CGN | 6615 | PTPRT | 8083 | PAK1IP1 |
| 744 | BCKDK | 2212 | CXCR2P1 | 3680 | PXT1 | 5148 | MDGA2 | 6616 | TMPRSS9 | 8084 | ZNF420 |
| 745 | POLR2J4 | 2213 | TMEM150C | 3681 | ZNF493 | 5149 | UBA5 | 6617 | DLG4 | 8085 | TMEM27 |
| 746 | C13ORF15 | 2214 | FLJ40039 | 3682 | PTGDR | 5150 | PLEKHG7 | 6618 | LOC101929488 | 8086 | BLVRB |
| 747 | C1ORF131 | 2215 | TRDMT1 | 3683 | APOBEC3D | 5151 | ENTPD7 | 6619 | FUT9 | 8087 | BTG3 |
| 748 | RBCK1 | 2216 | LOC728323 | 3684 | RBM10 | 5152 | CHRD | 6620 | PCDH8 | 8088 | CFAP36 |
| 749 | PAIP2 | 2217 | ZNF254 | 3685 | GZMH | 5153 | SPRY2 | 6621 | LRRC27 | 8089 | RFK |
| 750 | RAB5A | 2218 | CACNA1H | 3686 | ANKRD36B | 5154 | MUSTN1 | 6622 | CNOT10 | 8090 | ZFP1 |
| 751 | PCGF6 | 2219 | UHRF1 | 3687 | LOC100133089 | 5155 | RAD54L | 6623 | ZDHHC7 | 8091 | LARP4 |
| 752 | PCMT1 | 2220 | FRG2B | 3688 | DCDC2C | 5156 | VSIG8 | 6624 | WDR70 | 8092 | HCCS |
| 753 | DIS3L2 | 2221 | LINC00487 | 3689 | LRRC42 | 5157 | PARP2 | 6625 | SMO | 8093 | KLHL7 |
| 754 | ALG2 | 2222 | C4ORF40 | 3690 | PCDHGB3 | 5158 | SLC2A11 | 6626 | ASTL | 8094 | PRKDC |
| 755 | KIAA0415 | 2223 | LOC100130539 | 3691 | KHSRP | 5159 | SETD1A | 6627 | NT5C3B | 8095 | SLC30A5 |
| 756 | ARID5B | 2224 | UMOD | 3692 | ISLR2 | 5160 | ALLC | 6628 | AMBP | 8096 | ATP2B1 |
| 757 | TUBB4B | 2225 | SEMA3B | 3693 | LOC644450 | 5161 | PIGN | 6629 | TOX2 | 8097 | ARL6 |
| 758 | TGIF2 | 2226 | GREB1 | 3694 | C7ORF31 | 5162 | ACAT2 | 6630 | SIDT2 | 8098 | CDC37L1 |
| 759 | DPYD | 2227 | SH3PXD2A | 3695 | CALCRL | 5163 | GPR183 | 6631 | NEK11 | 8099 | MOB1B |
| 760 | CARS | 2228 | NCR3 | 3696 | FAT3 | 5164 | USP28 | 6632 | MCM5 | 8100 | UFM1 |
| 761 | CUL4B | 2229 | REST | 3697 | BRD4 | 5165 | SLC23A1 | 6633 | DAAM2 | 8101 | FAM72D |
| 762 | SNX6 | 2230 | VN1R4 | 3698 | PRDM2 | 5166 | RNU6-522P | 6634 | SELENBP1 | 8102 | LIN7C |
| 763 | SGSM3 | 2231 | KRTAP5-11 | 3699 | MCART6 | 5167 | LINGO2 | 6635 | UBTF | 8103 | LRRC40 |
| 764 | USP5 | 2232 | IGF2-AS | 3700 | PCDHA11 | 5168 | ALDOC | 6636 | OTX1 | 8104 | ABHD17B |
| 765 | OR4K14 | 2233 | ZDHHC20 | 3701 | UCA1 | 5169 | SC5D | 6637 | GABRB3 | 8105 | AGR3 |
| 766 | ADAT1 | 2234 | WTAP | 3702 | KRTAP5-6 | 5170 | DMRTC1 | 6638 | KSR1 | 8106 | ACAD8 |
| 767 | ABCF2 | 2235 | FP588 | 3703 | DKFZP451A211 | 5171 | CENPC | 6639 | MUT | 8107 | RBBP6 |
| 768 | HACE1 | 2236 | CSNK2A1 | 3704 | OPRL1 | 5172 | FRMD5 | 6640 | C15ORF27 | 8108 | LOC101930164 |
| 769 | RNH1 | 2237 | RSPH6A | 3705 | HIST2H2BE | 5173 | SLC25A25 | 6641 | AC005027.3 | 8109 | EIF4E |
| 770 | OSCP1 | 2238 | SPATC1 | 3706 | MRGPRE | 5174 | LPAR6 | 6642 | STAC3 | 8110 | DDX3X |
| 771 | RNF111 | 2239 | PRR3 | 3707 | NAV2-AS4 | 5175 | LOC100506459 | 6643 | CPXM1 | 8111 | DBR1 |
| 772 | NAPRT1 | 2240 | GNN | 3708 | LOC283888 | 5176 | SPHK1 | 6644 | DEFB1 | 8112 | SUMO2 |
| 773 | PRKY | 2241 | UBQLN4 | 3709 | KRTAP4-3 | 5177 | LINC00972 | 6645 | CNGA3 | 8113 | CCDC91 |
| 774 | EPHA2 | 2242 | ING5 | 3710 | EFCAB4B | 5178 | SLC17A3 | 6646 | ANKRD45 | 8114 | ZNF430 |
| 775 | BRMS1 | 2243 | COL27A1 | 3711 | SHANK3 | 5179 | STRC | 6647 | CTDSPL | 8115 | CKAP2 |
| 776 | NINJ2 | 2244 | LOC285762 | 3712 | FLJ42393 | 5180 | CSMD2 | 6648 | HDAC6 | 8116 | TMEM133 |
| 777 | OAF | 2245 | TNFRSF25 | 3713 | FLJ32224 | 5181 | UBE2D3 | 6649 | RAB11FIP4 | 8117 | NUP54 |
| 778 | SFTPA1 | 2246 | FLJ26332 | 3714 | MYO3B | 5182 | MAP3K19 | 6650 | SNX25 | 8118 | CAT |
| 779 | RPL22 | 2247 | SCN5A | 3715 | LOC728254 | 5183 | ACTR6 | 6651 | SEPT5 | 8119 | YTHDC1 |
| 780 | C17ORF62 | 2248 | ASCL4 | 3716 | SCARNA1 | 5184 | MRVI1 | 6652 | HGH1 | 8120 | ARHGAP18 |
| 781 | PRRC2A | 2249 | LOC643797 | 3717 | SNORD8 | 5185 | DPH2 | 6653 | KIAA0513 | 8121 | C3ORF14 |
| 782 | FAM134A | 2250 | HAND2 | 3718 | CBFA2T2 | 5186 | FLOT1 | 6654 | IZUMO1 | 8122 | BCAS2 |
| 783 | PCOLCE | 2251 | PRCC | 3719 | NUAK1 | 5187 | LTBP3 | 6655 | LOC646670 | 8123 | EPB41L2 |
| 784 | RPA2 | 2252 | CCDC132 | 3720 | SNORA16B | 5188 | DTNB | 6656 | PM20D1 | 8124 | FLI1 |
| 785 | DCK | 2253 | C6ORF163 | 3721 | SCARNA23 | 5189 | BRAF | 6657 | TAGLN3 | 8125 | PURA |
| 786 | TPM1 | 2254 | TMEM135 | 3722 | LOC142937 | 5190 | WDR16 | 6658 | C9ORF3 | 8126 | ZBTB2 |
| 787 | UNC50 | 2255 | PRO1596 | 3723 | DKFZP547G183 | 5191 | PDGFA | 6659 | TUBGCP5 | 8127 | HOMER1 |
| 788 | YIF1A | 2256 | LOC400752 | 3724 | EPHA6 | 5192 | CCDC93 | 6660 | B4GALNT1 | 8128 | GNG10 |
| 789 | SPATA2 | 2257 | NLRP8 | 3725 | TP53TG3 | 5193 | BRIX1 | 6661 | PEX5 | 8129 | IGBP1P1 |
| 790 | KIAA2013 | 2258 | FNIP1 | 3726 | LOC286109 | 5194 | CD209 | 6662 | FSTL1 | 8130 | SEPP1 |
| 791 | FIBP | 2259 | SAMD4A | 3727 | KIAA1875 | 5195 | ZWILCH | 6663 | SLC5A5 | 8131 | LEP |
| 792 | DHPS | 2260 | C8G | 3728 | LOC100130992 | 5196 | SLC37A3 | 6664 | SLC26A4 | 8132 | TUSC3 |
| 793 | SH2B3 | 2261 | ATP1A1 | 3729 | DNM1 | 5197 | CCDC175 | 6665 | ITLN2 | 8133 | NCBP2 |
| 794 | C2ORF76 | 2262 | LPPR4 | 3730 | UPK3A | 5198 | EIF4B | 6666 | RALGAPB | 8134 | SNORD80 |
| 795 | CHID1 | 2263 | FAT4 | 3731 | ELK1 | 5199 | CCDC120 | 6667 | LOC400997 | 8135 | GTF2H1 |
| 796 | LHX9 | 2264 | GTF2IRD2B | 3732 | MECOM | 5200 | FAM132A | 6668 | THYN1 | 8136 | IL17RD |
| 797 | CD300C | 2265 | NOTO | 3733 | LOC285965 | 5201 | NUP98 | 6669 | STYK1 | 8137 | ZNRF2 |
| 798 | LYSMD2 | 2266 | ERGIC1 | 3734 | LOC401037 | 5202 | TTLL2 | 6670 | LOC101929216 | 8138 | TUBE1 |
| 799 | SLC39A14 | 2267 | CHIC1 | 3735 | OIT3 | 5203 | SSU72 | 6671 | FCRL6 | 8139 | HSPA13 |
| 800 | GJB1 | 2268 | ZNF138 | 3736 | PTCD3 | 5204 | SMPDL3B | 6672 | PHACTR3 | 8140 | TSC22D1 |
| 801 | SNAR-B1 | 2269 | FARP1 | 3737 | NKD2 | 5205 | DRD3 | 6673 | TSPAN8 | 8141 | FAHD2A |
| 802 | ALPL | 2270 | P2RY4 | 3738 | AXIN2 | 5206 | RABEPK | 6674 | GLI3 | 8142 | KCNS3 |
| 803 | TRIM16L | 2271 | ARHGAP21 | 3739 | CYYR1 | 5207 | SCLT1 | 6675 | AFP | 8143 | CCAR1 |
| 804 | PRPF31 | 2272 | GRIP1 | 3740 | OFD1 | 5208 | KRAS | 6676 | COL4A4 | 8144 | POT1 |
| 805 | UAP1 | 2273 | NRG2 | 3741 | LDHAL6A | 5209 | TIMM9 | 6677 | ITIH6 | 8145 | FAM122A |
| 806 | HNRNPH2 | 2274 | SCAND2 | 3742 | AADACL3 | 5210 | TEX10 | 6678 | LYZ | 8146 | MIAT |
| 807 | SKIV2L | 2275 | FAM190A | 3743 | CELF6 | 5211 | HHIPL2 | 6679 | GALK1 | 8147 | DHFR2 |
| 808 | GPR137 | 2276 | C9ORF64 | 3744 | AFF3 | 5212 | ZFAND4 | 6680 | MAP2K3 | 8148 | CASP6 |
| 809 | PARP16 | 2277 | ACER2 | 3745 | OPN5 | 5213 | TARS | 6681 | MLXIP | 8149 | AKAP12 |
| 810 | LGMN | 2278 | ZNF793 | 3746 | ABCA8 | 5214 | GUSB | 6682 | STPG2 | 8150 | KITLG |
| 811 | SSR2 | 2279 | PDX1 | 3747 | RABGAP1L | 5215 | TLX1 | 6683 | SIAE | 8151 | PTMA |
| 812 | PEX11G | 2280 | FAM129C | 3748 | COL5A1 | 5216 | MAP3K12 | 6684 | SENP5 | 8152 | LYSMD3 |
| 813 | YKT6 | 2281 | WIPF1 | 3749 | FAM118A | 5217 | RUNX3 | 6685 | SUPT5H | 8153 | HDAC4 |
| 814 | C10ORF25 | 2282 | LOC100128501 | 3750 | FBXW12 | 5218 | VCAN | 6686 | INTS10 | 8154 | PAPSS1 |
| 815 | EGF | 2283 | IL28A | 3751 | OR5AR1 | 5219 | ACAP1 | 6687 | EBF4 | 8155 | RPLP1 |
| 816 | ARL6IP1 | 2284 | DEFA9P | 3752 | EPS15 | 5220 | TTK | 6688 | CA11 | 8156 | DCAF13 |
| 817 | PSMB8 | 2285 | LOC619207 | 3753 | SSPO | 5221 | LILRB5 | 6689 | RAB6B | 8157 | SBDS |
| 818 | HDDC2 | 2286 | STAM | 3754 | KIR2DL2 | 5222 | ATP6V1C1 | 6690 | MEIS2 | 8158 | NLK |
| 819 | RBM7 | 2287 | SON | 3755 | TBC1D10B | 5223 | RP11-120C12.3 | 6691 | TLE6 | 8159 | PNRC2 |
| 820 | NDUFA9 | 2288 | CCDC129 | 3756 | SH3GL3 | 5224 | AGRN | 6692 | NT5DC1 | 8160 | POU2AF1 |
| 821 | HK1 | 2289 | LOC494150 | 3757 | APBA2 | 5225 | RDH14 | 6693 | GPATCH2 | 8161 | TSEN15 |
| 822 | SDAD1 | 2290 | EEF2K | 3758 | SMG5 | 5226 | MKKS | 6694 | ADRB2 | 8162 | FAIM |
| 823 | MGAT1 | 2291 | MUC6 | 3759 | SRGAP2P2 | 5227 | HMHA1 | 6695 | RBL2 | 8163 | SNORD36A |
| 824 | CHST11 | 2292 | KLK2 | 3760 | KRTAP20-1 | 5228 | FRMD7 | 6696 | MCHR2-AS1 | 8164 | CAPZA1 |
| 825 | CD1E | 2293 | LOC100130894 | 3761 | AUTS2 | 5229 | ENTPD1-AS1 | 6697 | LOC101928220 | 8165 | MSANTD2 |
| 826 | RGNEF | 2294 | DCT | 3762 | MYOZ3 | 5230 | CLUL1 | 6698 | CTB-118N6.3 | 8166 | ROCK1 |
| 827 | TREM1 | 2295 | SPIN4 | 3763 | LOC100128869 | 5231 | MAOA | 6699 | CNTNAP2 | 8167 | H3F3AP4 |
| 828 | MTX2 | 2296 | ACVR1C | 3764 | PCDH11X | 5232 | C2CD5 | 6700 | BCS1L | 8168 | NAA50 |
| 829 | DLGAP5 | 2297 | PTGFR | 3765 | ZNF527 | 5233 | NPHP1 | 6701 | TBC1D4 | 8169 | PCDHB9 |
| 830 | C1ORF38 | 2298 | ZNF141 | 3766 | KRT25 | 5234 | RAPGEF6 | 6702 | LINC00536 | 8170 | SLC40A1 |
| 831 | MGMT | 2299 | SCARNA7 | 3767 | LOC100132495 | 5235 | OBFC1 | 6703 | TRUB1 | 8171 | PPP3CA |
| 832 | EPHX4 | 2300 | PACSIN1 | 3768 | SLC38A1 | 5236 | UPK1B | 6704 | MUM1 | 8172 | KCNK1 |
| 833 | ATP6V1B2 | 2301 | SORBS1 | 3769 | OR4F6 | 5237 | WDR65 | 6705 | SACS | 8173 | C11ORF58 |
| 834 | KCTD14 | 2302 | TMEM125 | 3770 | CECR9 | 5238 | C7 | 6706 | TMEM53 | 8174 | HMGN3 |
| 835 | SLC25A11 | 2303 | SBK1 | 3771 | MS4A10 | 5239 | TMPRSS15 | 6707 | NTN3 | 8175 | MTF2 |
| 836 | RIC8B | 2304 | C11ORF9 | 3772 | TBXA2R | 5240 | NOS2 | 6708 | L3MBTL4 | 8176 | FBXO3 |
| 837 | PIKFYVE | 2305 | ZNF599 | 3773 | HIP1 | 5241 | PPEF2 | 6709 | PKHD1 | 8177 | ZBTB44 |
| 838 | PPP2R2A | 2306 | CYP2W1 | 3774 | INADL | 5242 | ZGRF1 | 6710 | DLG5 | 8178 | FAM26F |
| 839 | EI24 | 2307 | LOC100129113 | 3775 | ZNF710 | 5243 | TESC | 6711 | PSMD9 | 8179 | MICU3 |
| 840 | LYPLA1 | 2308 | MATN3 | 3776 | TRIM39 | 5244 | LINC00992 | 6712 | SUGT1 | 8180 | CSNK1A1 |
| 841 | RAD17 | 2309 | ZNF621 | 3777 | SLC25A2 | 5245 | MMD2 | 6713 | OR7E47P | 8181 | HNRNPC |
| 842 | OAS1 | 2310 | NPAS3 | 3778 | AKNA | 5246 | ZFYVE21 | 6714 | TNC | 8182 | MPLKIP |
| 843 | AHSA1 | 2311 | VSTM2A | 3779 | SCARNA6 | 5247 | HAPLN3 | 6715 | BRCA2 | 8183 | FTH1P3 |
| 844 | POLR2B | 2312 | ZNF573 | 3780 | HOXB3 | 5248 | SPOPL | 6716 | ELP2 | 8184 | PLCL1 |
| 845 | MUM1L1 | 2313 | ARHGAP23 | 3781 | ZNF486 | 5249 | TSPY1 | 6717 | SPR | 8185 | CA3 |
| 846 | TAS1R3 | 2314 | PCDHB12 | 3782 | HULC | 5250 | AKR1C3 | 6718 | TOR3A | 8186 | CYP51A1 |
| 847 | NR3C2 | 2315 | LOC286063 | 3783 | KRTAP10-2 | 5251 | ARHGEF12 | 6719 | THEG | 8187 | SLC25A16 |
| 848 | PRMT10 | 2316 | MRPL23-AS1 | 3784 | LOC374890 | 5252 | SLA | 6720 | SYP | 8188 | NTN4 |
| 849 | ADORA1 | 2317 | SNRPD3 | 3785 | RPA4 | 5253 | KMT2D | 6721 | PTK2 | 8189 | SMPDL3A |
| 850 | PDLIM1 | 2318 | COL23A1 | 3786 | C19ORF26 | 5254 | GCKR | 6722 | SSX1 | 8190 | DPYS |
| 851 | MED30 | 2319 | LOC145783 | 3787 | LRCH3 | 5255 | TMEM65 | 6723 | ABCC6 | 8191 | CNBP |
| 852 | NCEH1 | 2320 | MAP3K10 | 3788 | SNX22 | 5256 | PPP2R4 | 6724 | WDR55 | 8192 | CD302 |
| 853 | GNB2L1 | 2321 | GPR156 | 3789 | KRTAP10-4 | 5257 | IL17RA | 6725 | INSR | 8193 | RPL21P28 |
| 854 | CDKN3 | 2322 | LOC100128818 | 3790 | TRNT1 | 5258 | IL17REL | 6726 | EPS8L3 | 8194 | ANKRD44 |
| 855 | PPP1CA | 2323 | BLOC1S3 | 3791 | CNTN4 | 5259 | ACOT9 | 6727 | ATG2B | 8195 | MAPK9 |
| 856 | CLIC4 | 2324 | ZNF182 | 3792 | SPTBN4 | 5260 | CARS2 | 6728 | SRRM2-AS1 | 8196 | KIAA1033 |
| 857 | HSDL1 | 2325 | BMP8B | 3793 | APOL4 | 5261 | CAMTA1 | 6729 | TMCO6 | 8197 | GPBP1L1 |
| 858 | METTL23 | 2326 | LOC440864 | 3794 | PAR1 | 5262 | ARHGEF15 | 6730 | RP11-283C24.1 | 8198 | RPL23AP64 |
| 859 | RUFY1 | 2327 | SRP54 | 3795 | TSPYL5 | 5263 | FAM71D | 6731 | EPHB6 | 8199 | B3GNT5 |
| 860 | BLMH | 2328 | ATP7A | 3796 | ZNF560 | 5264 | CRTC2 | 6732 | KLHL18 | 8200 | CEP126 |
| 861 | CXCL17 | 2329 | SLC35G5 | 3797 | EWSR1 | 5265 | SLIT2 | 6733 | WSCD1 | 8201 | UTP3 |
| 862 | LOC286359 | 2330 | SNAP91 | 3798 | DCX | 5266 | TNNT3 | 6734 | CTGF | 8202 | KCTD12 |
| 863 | RSL24D1 | 2331 | SYT7 | 3799 | LOC143286 | 5267 | LMAN1 | 6735 | CPSF4 | 8203 | ARFGEF1 |
| 864 | KRCC1 | 2332 | LACTBL1 | 3800 | TBX21 | 5268 | GRB10 | 6736 | CCDC88C | 8204 | UNC5A |
| 865 | PDLIM3 | 2333 | KIAA1598 | 3801 | SRCIN1 | 5269 | GSAP | 6737 | CCDC159 | 8205 | SYAP1 |
| 866 | RELA | 2334 | TRIM25 | 3802 | FRMD4A | 5270 | ENTPD4 | 6738 | IPO8 | 8206 | ZNF181 |
| 867 | GTF3C1 | 2335 | OR11G2 | 3803 | OPHN1 | 5271 | AOX2P | 6739 | TMEM39B | 8207 | LOC728554 |
| 868 | PPME1 | 2336 | DDX6 | 3804 | SGK2 | 5272 | RAB4B-EGLN2 | 6740 | HHATL | 8208 | EPCAM |
| 869 | SEC63 | 2337 | SNORA80 | 3805 | MCART1 | 5273 | PRDM4 | 6741 | NOX5 | 8209 | GIMAP2 |
| 870 | SH3BGRL2 | 2338 | SPDYE5 | 3806 | TNNT1 | 5274 | EIF3H | 6742 | VCP | 8210 | PERP |
| 871 | MEMO1 | 2339 | KIR2DL5A | 3807 | BCOR | 5275 | ITGB4 | 6743 | TGM5 | 8211 | SMN1 |
| 872 | ST13 | 2340 | MFN2 | 3808 | SEC14L3 | 5276 | 8-Mar | 6744 | SLC12A9 | 8212 | ROBO1 |
| 873 | PNMA3 | 2341 | KSR2 | 3809 | NPLOC4 | 5277 | TECTA | 6745 | DCDC1 | 8213 | YY1 |
| 874 | MRPL36 | 2342 | RTN3 | 3810 | DUSP5P | 5278 | NCS1 | 6746 | SPTB | 8214 | TSPAN13 |
| 875 | GPN1 | 2343 | DSC3 | 3811 | TTTY19 | 5279 | ANKS3 | 6747 | PHF7 | 8215 | ARMC8 |
| 876 | ZNF581 | 2344 | LOC653160 | 3812 | AGBL4 | 5280 | NR4A1 | 6748 | DHRS9 | 8216 | TLR1 |
| 877 | TIMM23 | 2345 | FBP2 | 3813 | C4ORF50 | 5281 | EDC4 | 6749 | TMEM214 | 8217 | FBXO38 |
| 878 | SDHD | 2346 | ABTB1 | 3814 | C6ORF174 | 5282 | SPATA20 | 6750 | ACSM6 | 8218 | CWC22 |
| 879 | LCN8 | 2347 | CYTH1 | 3815 | COX6B2 | 5283 | MCM10 | 6751 | FAM207A | 8219 | ABAT |
| 880 | LSM2 | 2348 | RGS7BP | 3816 | LRRC37A2 | 5284 | LOC101926963 | 6752 | PCDH7 | 8220 | IDS |
| 881 | PLEKHJ1 | 2349 | RAD54B | 3817 | LOC100506191 | 5285 | RP11-554E23.2 | 6753 | CHRNE | 8221 | MYADM |
| 882 | QPCT | 2350 | RPP30 | 3818 | NOXRED1 | 5286 | ABCF3 | 6754 | CDK4 | 8222 | RNF219 |
| 883 | SCG2 | 2351 | LRRC7 | 3819 | HERC2P4 | 5287 | PCLO | 6755 | GDPD5 | 8223 | BLK |
| 884 | FUCA1 | 2352 | CUX2 | 3820 | ZNF587 | 5288 | NTRK1 | 6756 | RPL34-AS1 | 8224 | DNAJC1 |
| 885 | RPN1 | 2353 | RORA | 3821 | RASGEF1C | 5289 | ZNF8 | 6757 | ABCD3 | 8225 | ZNF681 |
| 886 | GBP3 | 2354 | LOC100134240 | 3822 | RN7SL1 | 5290 | IDO2 | 6758 | TBX22 | 8226 | TMEM67 |
| 887 | RGS2 | 2355 | REXO1L1 | 3823 | GLI2 | 5291 | DLEU1 | 6759 | KDM4A | 8227 | RPS6KA3 |
| 888 | MLF1IP | 2356 | NRN1 | 3824 | ANO1 | 5292 | OLFM3 | 6760 | NEK4 | 8228 | ATXN1 |
| 889 | SLC25A4 | 2357 | CNOT3 | 3825 | SRSF6 | 5293 | SDK2 | 6761 | SLC25A24P1 | 8229 | PLA2G2D |
| 890 | DMBT1 | 2358 | ZNF224 | 3826 | CPAMD8 | 5294 | SLC35F5 | 6762 | TEX11 | 8230 | MZT1 |
| 891 | TMED1 | 2359 | RNF216 | 3827 | LOC100129198 | 5295 | THRB | 6763 | ANO4 | 8231 | ATF1 |
| 892 | CFHR3 | 2360 | LOC728093 | 3828 | BCL2L11 | 5296 | REG3G | 6764 | TXLNA | 8232 | IL1RAP |
| 893 | ADAMTS4 | 2361 | INTS1 | 3829 | KRT78 | 5297 | KLHL22 | 6765 | ACCS | 8233 | ARHGAP29 |
| 894 | CCDC56 | 2362 | TTC16 | 3830 | ZNF385C | 5298 | C12ORF4 | 6766 | MTA1 | 8234 | MATN2 |
| 895 | CCDC24 | 2363 | KIAA0889 | 3831 | PLAC8 | 5299 | SH3RF1 | 6767 | ABCC8 | 8235 | BMP2K |
| 896 | SHMT1 | 2364 | ASMT | 3832 | LOC727721 | 5300 | ARAP3 | 6768 | OTOF | 8236 | ETAA1 |
| 897 | LTBR | 2365 | PLAGL2 | 3833 | NBLA00301 | 5301 | CDK2 | 6769 | KLRC1 | 8237 | ZNF23 |
| 898 | CRTC3 | 2366 | RNU4ATAC | 3834 | ZCCHC23 | 5302 | MCF2 | 6770 | AR | 8238 | FAM49B |
| 899 | SRSF2 | 2367 | LOC152578 | 3835 | TAC3 | 5303 | NR4A3 | 6771 | RNU1-122P | 8239 | THAP1 |
| 900 | AAMP | 2368 | MUC20 | 3836 | CLNK | 5304 | HMBS | 6772 | RP11-867G2.6 | 8240 | ACTG1P4 |
| 901 | GRAMD1A | 2369 | GRK5 | 3837 | ZSCAN2 | 5305 | ETV7 | 6773 | FOXH1 | 8241 | ACTR3 |
| 902 | TRAM1 | 2370 | LOC100129726 | 3838 | SHANK1 | 5306 | PAH | 6774 | ZNF804A | 8242 | NUDCD2 |
| 903 | GSDMD | 2371 | RTKN2 | 3839 | NOS1 | 5307 | SSPN | 6775 | CPA5 | 8243 | TOP1P2 |
| 904 | TPRG1L | 2372 | CTRC | 3840 | DKFZP547J222 | 5308 | ZNF268 | 6776 | LOC101927948 | 8244 | HSPA1A |
| 905 | TNFRSF1B | 2373 | ZNF664-FAM101A | 3841 | C9ORF170 | 5309 | CEP89 | 6777 | LIAS | 8245 | ARHGAP12 |
| 906 | MAPKAPK2 | 2374 | SKI | 3842 | LOC645202 | 5310 | TNFRSF9 | 6778 | SEMA6A | 8246 | ILK |
| 907 | GALM | 2375 | LOC100499221 | 3843 | OLFML2A | 5311 | KIAA1522 | 6779 | STYXL1 | 8247 | MSTO1 |
| 908 | LOC400657 | 2376 | GNAQ | 3844 | CECR3 | 5312 | LRP1B | 6780 | KLHL13 | 8248 | PHF3 |
| 909 | ZNF28 | 2377 | BMS1P1 | 3845 | OCR1 | 5313 | RNF32 | 6781 | ABCB5 | 8249 | ADAM10 |
| 910 | ELOVL1 | 2378 | LOC388948 | 3846 | TCF23 | 5314 | TMEM80 | 6782 | USP21 | 8250 | NEDD9 |
| 911 | ST3GAL3 | 2379 | MLL | 3847 | LOC100509196 | 5315 | AGXT2 | 6783 | NBEA | 8251 | SEPT7 |
| 912 | AIDA | 2380 | LOC100188947 | 3848 | PRY2 | 5316 | DNA2 | 6784 | CCDC151 | 8252 | RABGEF1 |
| 913 | SPRY4 | 2381 | POF1B | 3849 | TMED8 | 5317 | TGFBR1 | 6785 | SCARF2 | 8253 | USP25 |
| 914 | KCNQ2 | 2382 | MEAF6 | 3850 | BHLHA9 | 5318 | ASIC4 | 6786 | ERI3 | 8254 | RPL23AP87 |
| 915 | DPY19L1 | 2383 | RAPH1 | 3851 | LOC729737 | 5319 | NTM | 6787 | LOXL2 | 8255 | TSPAN12 |
| 916 | SLC35B2 | 2384 | LINC00494 | 3852 | DNAH14 | 5320 | LASP1 | 6788 | ZFP90 | 8256 | AGTPBP1 |
| 917 | BAI3 | 2385 | IGSF5 | 3853 | KLK8 | 5321 | ZNF624 | 6789 | MCMDC2 | 8257 | TMEM167A |
| 918 | ALOX5AP | 2386 | HDGFRP2 | 3854 | C15ORF5 | 5322 | SH3D19 | 6790 | STON2 | 8258 | ARL5B |
| 919 | TMEM126B | 2387 | CNRIP1 | 3855 | GLT25D2 | 5323 | EIF4E3 | 6791 | RBFOX1 | 8259 | MLF1 |
| 920 | UBAC2 | 2388 | LOC255480 | 3856 | NOS1AP | 5324 | MTMR11 | 6792 | MYLK | 8260 | FAM179B |
| 921 | TCEA2 | 2389 | HPSE2 | 3857 | LOC100131066 | 5325 | PDK1 | 6793 | SEMA3D | 8261 | CCNH |
| 922 | TMCO3 | 2390 | ANKRD20A9P | 3858 | PPP1R12B | 5326 | CNTNAP1 | 6794 | PROM1 | 8262 | ATXN10 |
| 923 | ERGIC2 | 2391 | C17ORF66 | 3859 | LOC643923 | 5327 | ALB | 6795 | TMOD4 | 8263 | SLTM |
| 924 | PLEKHG1 | 2392 | FBXL18 | 3860 | FLJ37786 | 5328 | KLHL36 | 6796 | IFT172 | 8264 | OMA1 |
| 925 | TMEM33 | 2393 | NUDT9P1 | 3861 | LOC338667 | 5329 | FBN1 | 6797 | UCHL5 | 8265 | PMAIP1 |
| 926 | ITGAE | 2394 | PDHA1 | 3862 | GAL | 5330 | BBS1 | 6798 | PPP2R5A | 8266 | AMY2B |
| 927 | CNPY3 | 2395 | LOC100131831 | 3863 | PIP5K1A | 5331 | MTTP | 6799 | ARSG | 8267 | GAS1 |
| 928 | GZF1 | 2396 | LOC283352 | 3864 | CST9L | 5332 | CLDND1 | 6800 | ZNF534 | 8268 | PIK3AP1 |
| 929 | PPM1H | 2397 | CDC14B | 3865 | FLJ39653 | 5333 | STK32B | 6801 | MS4A8 | 8269 | CDKN1B |
| 930 | CREBL2 | 2398 | COL11A2 | 3866 | FAM167A | 5334 | STK11IP | 6802 | TMEM132E | 8270 | ZNF302 |
| 931 | KIAA1244 | 2399 | GHRLOS | 3867 | SMCR6 | 5335 | HSPBAP1 | 6803 | GPR110 | 8271 | PLCG1 |
| 932 | C5ORF15 | 2400 | LOC100132790 | 3868 | ZMYND11 | 5336 | PCSK5 | 6804 | DNAJC27 | 8272 | MOSPD1 |
| 933 | TUB | 2401 | LOC158435 | 3869 | ATOH8 | 5337 | SMARCA1 | 6805 | GNG12-AS1 | 8273 | GPBP1 |
| 934 | SEC23A | 2402 | MED12L | 3870 | KRT74 | 5338 | IGHMBP2 | 6806 | TSFM | 8274 | WRB |
| 935 | LOC100130348 | 2403 | TCOF1 | 3871 | NTNG2 | 5339 | LOC101928576 | 6807 | VANGL1 | 8275 | ID4 |
| 936 | MANBAL | 2404 | RLIM | 3872 | LOC653061 | 5340 | HAX1 | 6808 | MUC5B | 8276 | ZNF204P |
| 937 | RNF14 | 2405 | THSD1 | 3873 | NRADDP | 5341 | CDKL5 | 6809 | BBS7 | 8277 | PCDHB10 |
| 938 | IL34 | 2406 | LOC401098 | 3874 | LINC00173 | 5342 | GOLGA8CP | 6810 | ERLIN2 | 8278 | ARMT1 |
| 939 | PSMF1 | 2407 | RECQL | 3875 | CACNB2 | 5343 | ABHD17C | 6811 | OR3A2 | 8279 | UEVLD |
| 940 | DUSP1 | 2408 | SUZ12P | 3876 | PGF | 5344 | ITPR3 | 6812 | PCDH15 | 8280 | FABP5 |
| 941 | AIMP2 | 2409 | WWTR1 | 3877 | AATK | 5345 | GPHN | 6813 | CRISPLD1 | 8281 | SLC2A14 |
| 942 | CCNB1 | 2410 | LOC731932 | 3878 | FAM163B | 5346 | ULK1 | 6814 | WNT5B | 8282 | MAPT |
| 943 | HSD17B6 | 2411 | PHLDB3 | 3879 | SNX31 | 5347 | TTC27 | 6815 | SLC9C1 | 8283 | PDE8A |
| 944 | RPS3A | 2412 | ATRX | 3880 | KLF14 | 5348 | SLC38A9 | 6816 | RP4-537K23.4 | 8284 | PSG3 |
| 945 | PAMR1 | 2413 | SPAG9 | 3881 | ARGFXP2 | 5349 | NELL2 | 6817 | DFFA | 8285 | COPS2 |
| 946 | COPS8 | 2414 | RASA4 | 3882 | FAM106A | 5350 | CEP290 | 6818 | POLA1 | 8286 | R3HDM1 |
| 947 | BSCL2 | 2415 | CELF5 | 3883 | LOC146880 | 5351 | TECPR1 | 6819 | TTBK1 | 8287 | DAPP1 |
| 948 | TWF1 | 2416 | PRDM11 | 3884 | ZCCHC6 | 5352 | HMGXB4 | 6820 | ZNF280C | 8288 | GKAP1 |
| 949 | MAP1LC3C | 2417 | MS4A14 | 3885 | LOC389607 | 5353 | ZBTB17 | 6821 | LSAMP | 8289 | FBXO30 |
| 950 | RPS3 | 2418 | LOC401557 | 3886 | CCNC | 5354 | COL14A1 | 6822 | TLR3 | 8290 | LOC644936 |
| 951 | POR | 2419 | ANKAR | 3887 | RNASET2 | 5355 | EIF2AK3 | 6823 | SLC25A12 | 8291 | RABL2A |
| 952 | NHP2L1 | 2420 | RUNX1 | 3888 | C10ORF113 | 5356 | NUMA1 | 6824 | LYPD6B | 8292 | SGCD |
| 953 | COPS5 | 2421 | VCY | 3889 | LHX1 | 5357 | KIFC2 | 6825 | ANKRD23 | 8293 | ZC3H7A |
| 954 | RPP38 | 2422 | LOC100130276 | 3890 | H2BFXP | 5358 | ZBTB8A | 6826 | WFDC3 | 8294 | TMEM263 |
| 955 | SQSTM1 | 2423 | EYA3 | 3891 | LOC100128398 | 5359 | OR2H1 | 6827 | LOC284395 | 8295 | ZEB2 |
| 956 | LYPLA2 | 2424 | PSMD5 | 3892 | GPR114 | 5360 | SPECC1 | 6828 | CSTF2T | 8296 | PTGES3 |
| 957 | KDELR3 | 2425 | ZC3H14 | 3893 | TSPYL6 | 5361 | PLCB4 | 6829 | TRAPPC6A | 8297 | THUMPD1 |
| 958 | CCND3 | 2426 | SF3A2 | 3894 | LOC731789 | 5362 | CALML6 | 6830 | LOC101928102 | 8298 | ZNF658 |
| 959 | PPP4R1 | 2427 | DENND3 | 3895 | LOC100128811 | 5363 | CASP5 | 6831 | ACTR3B | 8299 | NKAP |
| 960 | CRIP2 | 2428 | SCD5 | 3896 | LUC7L | 5364 | ATP6V1G2-DDX39B | 6832 | PSD | 8300 | RAX2 |
| 961 | PLSCR3 | 2429 | LINC00052 | 3897 | LOC100288884 | 5365 | FNBP1L | 6833 | VWF | 8301 | EPS8 |
| 962 | NIPSNAP3B | 2430 | HN1L | 3898 | POM121L8P | 5366 | ACCSL | 6834 | PLA2G16 | 8302 | DSTYK |
| 963 | ARPP19 | 2431 | ATMIN | 3899 | SORCS3 | 5367 | GAS2L1 | 6835 | PVRL3 | 8303 | FZD6 |
| 964 | OAT | 2432 | MARK2 | 3900 | NPCDR1 | 5368 | NHSL1 | 6836 | KIAA1109 | 8304 | ST13P4 |
| 965 | DUSP14 | 2433 | RALBP1 | 3901 | ZNF2 | 5369 | IQCJ-SCHIP1 | 6837 | DPP10 | 8305 | PDIA3P1 |
| 966 | MRPL38 | 2434 | FLJ36777 | 3902 | BCL9L | 5370 | ATXN7L1 | 6838 | FAM19A4 | 8306 | GNA13 |
| 967 | HLA-DRA | 2435 | SPDYE1 | 3903 | RAB37 | 5371 | MRPS22 | 6839 | USP36 | 8307 | HS2ST1 |
| 968 | LOC100505564 | 2436 | TFPI2 | 3904 | EGFEM1P | 5372 | CYP1A1 | 6840 | DDX27 | 8308 | NACAP1 |
| 969 | LEPROT | 2437 | ZSWIM7 | 3905 | CD99L2 | 5373 | RNU7-40P | 6841 | PRPF19 | 8309 | GLT8D1 |
| 970 | PTP4A1 | 2438 | PCNXL2 | 3906 | LOC100190986 | 5374 | CCDC180 | 6842 | HSPE1-MOB4 | 8310 | CCL19 |
| 971 | RNGTT | 2439 | ST3GAL5 | 3907 | PRRT2 | 5375 | LOC102724663 | 6843 | TP53TG5 | 8311 | ANXA4 |
| 972 | PFKL | 2440 | ZNF169 | 3908 | MGC39584 | 5376 | CLIP2 | 6844 | FGF10 | 8312 | TRMT1L |
| 973 | KCNJ5 | 2441 | LOC283713 | 3909 | TAF5L | 5377 | CTCFL | 6845 | AC005276.1 | 8313 | ATP11C |
| 974 | PARP1 | 2442 | PRO0611 | 3910 | CELF1 | 5378 | LOC102724629 | 6846 | ELAC2 | 8314 | MEOX2 |
| 975 | PSMC4 | 2443 | PNISR | 3911 | NYNRIN | 5379 | PLD1 | 6847 | RPA3 | 8315 | ECT2 |
| 976 | ELMOD3 | 2444 | MXD3 | 3912 | RN7SK | 5380 | TMCC3 | 6848 | PIBF1 | 8316 | TP63 |
| 977 | SNX9 | 2445 | LOC642826 | 3913 | BOD1P | 5381 | AC091729.8 | 6849 | HDAC10 | 8317 | SREK1 |
| 978 | CPEB4 | 2446 | ORMDL1 | 3914 | TNFSF8 | 5382 | TTLL4 | 6850 | CDKL3 | 8318 | TSPAN10 |
| 979 | VTA1 | 2447 | ABCC6P1 | 3915 | ARL17B | 5383 | PLEKHG3 | 6851 | GPR85 | 8319 | PABPC3 |
| 980 | CRK | 2448 | HAPLN2 | 3916 | RPS16P5 | 5384 | BTNL9 | 6852 | NECAB3 | 8320 | RAB6A |
| 981 | ALDH5A1 | 2449 | PLEKHM1 | 3917 | H2AFB3 | 5385 | ITGA7 | 6853 | PROK1 | 8321 | CCNL1 |
| 982 | C11ORF96 | 2450 | SEMA6C | 3918 | LINC00355 | 5386 | CASKIN2 | 6854 | P4HA2 | 8322 | LCOR |
| 983 | MXRA7 | 2451 | SLITRK2 | 3919 | LOC100132077 | 5387 | DEPDC5 | 6855 | ARL5A | 8323 | ZNF226 |
| 984 | JUNB | 2452 | ACACB | 3920 | ZNF847P | 5388 | PACRG | 6856 | USP39 | 8324 | RBM47 |
| 985 | GLS | 2453 | ZNF677 | 3921 | IGLON5 | 5389 | SPAG5 | 6857 | SLC13A5 | 8325 | DICER1 |
| 986 | LOC285943 | 2454 | HBZ | 3922 | LINC00273 | 5390 | SMARCC1 | 6858 | PLXDC2 | 8326 | CENPQ |
| 987 | TGFB1 | 2455 | ZNF496 | 3923 | HTATSF1P2 | 5391 | EMCN | 6859 | LAMB2 | 8327 | HECA |
| 988 | FCHO2 | 2456 | KLHL4 | 3924 | TEPP | 5392 | RPL8 | 6860 | RP11-1069G10.1 | 8328 | CLTC |
| 989 | RRM2B | 2457 | ABCA9 | 3925 | TACR1 | 5393 | SLC15A2 | 6861 | RGPD2 | 8329 | ASUN |
| 990 | SDHA | 2458 | MACROD2 | 3926 | MASP2 | 5394 | RIMBP2 | 6862 | PPP1R37 | 8330 | FAM162A |
| 991 | INTS5 | 2459 | POGZ | 3927 | LOC100133857 | 5395 | DCN | 6863 | PRMT7 | 8331 | NOL8 |
| 992 | MAST4 | 2460 | GEMIN2 | 3928 | LOC146795 | 5396 | PLXDC1 | 6864 | LNP1 | 8332 | TATDN1 |
| 993 | PCBP4 | 2461 | GTF2A1 | 3929 | LOC100128164 | 5397 | CRADD | 6865 | RNF150 | 8333 | AZIN1 |
| 994 | MAGI2 | 2462 | GNAI3 | 3930 | CYMP | 5398 | MAP2K5 | 6866 | FCRL1 | 8334 | CD19 |
| 995 | FBXO6 | 2463 | SPACA7 | 3931 | LOC644727 | 5399 | NRDE2 | 6867 | ZMYM3 | 8335 | MYBL1 |
| 996 | OXCT1 | 2464 | ARHGAP19 | 3932 | GNRHR | 5400 | RFX8 | 6868 | ARHGEF25 | 8336 | PCDHB16 |
| 997 | RBM24 | 2465 | CHORDC1 | 3933 | PSPC1 | 5401 | CCDC9 | 6869 | DENND6B | 8337 | DCAF6 |
| 998 | ZNF259 | 2466 | OPTC | 3934 | FLJ45340 | 5402 | SUV39H1 | 6870 | DOCK1 | 8338 | CXCR5 |
| 999 | DNAJB6 | 2467 | FBXL2 | 3935 | LOC441493 | 5403 | TMEM241 | 6871 | C19ORF83 | 8339 | TRAPPC6B |
| 1000 | PEMT | 2468 | WWOX | 3936 | FRAS1 | 5404 | RAVER2 | 6872 | KIAA1211L | 8340 | DPP4 |
| 1001 | GRB2 | 2469 | DUX4 | 3937 | C14ORF135 | 5405 | MGAT4C | 6873 | OVCH1 | 8341 | CCDC90B |
| 1002 | SYPL1 | 2470 | FAM166A | 3938 | ADAMTS7 | 5406 | DHCR24 | 6874 | CDC42BPA | 8342 | CXCL11 |
| 1003 | FEN1 | 2471 | TSPAN14 | 3939 | ZBTB8OS | 5407 | VASH2 | 6875 | CDAN1 | 8343 | TTC30B |
| 1004 | GNAI1 | 2472 | UNQ9370 | 3940 | RNU105B | 5408 | EXTL3-AS1 | 6876 | SNX30 | 8344 | BPIFA1 |
| 1005 | TXNRD2 | 2473 | LOC651337 | 3941 | LOC100129846 | 5409 | HTR3E-AS1 | 6877 | MALRD1 | 8345 | MBLAC2 |
| 1006 | EP400 | 2474 | INO80E | 3942 | PHYHIPL | 5410 | MYO10 | 6878 | ZFR | 8346 | MMP11 |
| 1007 | FURIN | 2475 | C22ORF37 | 3943 | LOC100130193 | 5411 | CCDC154 | 6879 | SVOP | 8347 | SFRP4 |
| 1008 | PTBP1 | 2476 | ERG | 3944 | FBN3 | 5412 | UPF3B | 6880 | GALNT15 | 8348 | MSL2 |
| 1009 | OGG1 | 2477 | C20ORF26 | 3945 | HHLA2 | 5413 | SLU7 | 6881 | FRMPD1 | 8349 | CLECL1 |
| 1010 | PARD6B | 2478 | OR10A5 | 3946 | LOC441025 | 5414 | MS4A12 | 6882 | LRRC75A-AS1 | 8350 | BBOF1 |
| 1011 | NADK | 2479 | CRYGN | 3947 | USHBP1 | 5415 | TNFAIP8 | 6883 | FAM105A | 8351 | DENND4C |
| 1012 | COMT | 2480 | LOC646034 | 3948 | CLDN9 | 5416 | STKLD1 | 6884 | SEC23IP | 8352 | G3BP1 |
| 1013 | EIF4G1 | 2481 | KIAA1654 | 3949 | LOC400743 | 5417 | SLC38A7 | 6885 | CEBPZ | 8353 | ARMCX1 |
| 1014 | PHF20L1 | 2482 | DDX52 | 3950 | C8ORF50 | 5418 | MUC19 | 6886 | TGFBR3 | 8354 | CD79A |
| 1015 | RNF114 | 2483 | AHNAK2 | 3951 | FLJ13197 | 5419 | CES5A | 6887 | PIGK | 8355 | RAD21 |
| 1016 | SHROOM2 | 2484 | LOC200726 | 3952 | LOC100129995 | 5420 | TULP2 | 6888 | ANO5 | 8356 | CORIN |
| 1017 | TMEM64 | 2485 | GPR173 | 3953 | RMRP | 5421 | SFSWAP | 6889 | CPED1 | 8357 | USP6 |
| 1018 | NPAT | 2486 | MGC10814 | 3954 | LOC642980 | 5422 | FCN1 | 6890 | SCN3B | 8358 | PHLDB2 |
| 1019 | ZMYM6NB | 2487 | PRKAG2 | 3955 | KCNMA1 | 5423 | GNA12 | 6891 | SWAP70 | 8359 | RANBP2 |
| 1020 | TACC1 | 2488 | RASGRP2 | 3956 | C6ORF201 | 5424 | DNTTIP2 | 6892 | USP40 | 8360 | VPREB3 |
| 1021 | USP46 | 2489 | SYNE2 | 3957 | SPRR2E | 5425 | CASP16 | 6893 | IPO4 | 8361 | CIART |
| 1022 | HNRNPH3 | 2490 | SCARNA9L | 3958 | KLF12 | 5426 | STK16 | 6894 | PDGFRL | 8362 | HSPA4 |
| 1023 | TMEM185A | 2491 | LOC440104 | 3959 | MCOLN3 | 5427 | DACH1 | 6895 | AGA | 8363 | FAM174A |
| 1024 | DARS2 | 2492 | LOC100128640 | 3960 | LRRC39 | 5428 | CTSK | 6896 | SLMO1 | 8364 | RERGL |
| 1025 | C14ORF167 | 2493 | GAB1 | 3961 | PKLR | 5429 | SLC16A6 | 6897 | ZSCAN16-AS1 | 8365 | GIMAP1 |
| 1026 | FLCN | 2494 | LOC157562 | 3962 | BZRAP1 | 5430 | GPAT2 | 6898 | C3ORF18 | 8366 | DAPL1 |
| 1027 | PAK2 | 2495 | LOC100144604 | 3963 | LOC284576 | 5431 | NPPA | 6899 | SOX30 | 8367 | OSBPL11 |
| 1028 | LIPA | 2496 | ZNF594 | 3964 | LOC100134237 | 5432 | TBC1D23 | 6900 | C3ORF58 | 8368 | SSTR1 |
| 1029 | CNPPD1 | 2497 | TTC28-AS1 | 3965 | PPP1R27 | 5433 | ZNF75A | 6901 | DENND1C | 8369 | FCN3 |
| 1030 | ASPHD2 | 2498 | LY6G6E | 3966 | LOC400756 | 5434 | LRRC16B | 6902 | RECQL4 | 8370 | CEP57 |
| 1031 | TMEM194A | 2499 | LOC100129826 | 3967 | FAM198A | 5435 | CLEC12B | 6903 | BRD9 | 8371 | ALG11 |
| 1032 | STX3 | 2500 | CACYBP | 3968 | PREX2 | 5436 | LOC100499484 | 6904 | RNASEH2B | 8372 | HSD17B11 |
| 1033 | PSMC6 | 2501 | MAML3 | 3969 | ZNF292 | 5437 | ASNSD1 | 6905 | ICE2 | 8373 | FXYD3 |
| 1034 | LOC100129502 | 2502 | SETMAR | 3970 | UGT3A1 | 5438 | ENOX2 | 6906 | LRRIQ3 | 8374 | TMEM106B |
| 1035 | PPP1R2 | 2503 | C6ORF225 | 3971 | DNM1P46 | 5439 | DGKI | 6907 | ELMO3 | 8375 | HAS1 |
| 1036 | FAM168B | 2504 | APOL5 | 3972 | LOC100132966 | 5440 | RHBDF1 | 6908 | NSUN6 | 8376 | OGN |
| 1037 | FAM127B | 2505 | C1ORF35 | 3973 | LRRC37A3 | 5441 | CIR1 | 6909 | RAB11FIP5 | 8377 | C6ORF120 |
| 1038 | CYB5R4 | 2506 | KIAA1826 | 3974 | ZNF713 | 5442 | GTF2IRD2P1 | 6910 | SKOR1 | 8378 | HSPE1 |
| 1039 | RGS3 | 2507 | AHDC1 | 3975 | LOC100128792 | 5443 | MBP | 6911 | RERG | 8379 | VPS50 |
| 1040 | SNRPB2 | 2508 | LOC221814 | 3976 | TMEM184B | 5444 | SNRNP200 | 6912 | ZFAND1 | 8380 | DCSTAMP |
| 1041 | PSEN1 | 2509 | MRE11A | 3977 | C20ORF203 | 5445 | RAP1A | 6913 | TTLL5 | 8381 | WDR11 |
| 1042 | TSN | 2510 | ARID3A | 3978 | GARNL3 | 5446 | P2RY2 | 6914 | FSIP1 | 8382 | ZNF679 |
| 1043 | MZB1 | 2511 | ICMT | 3979 | CELA3A | 5447 | KIFC1 | 6915 | CENPI | 8383 | COL10A1 |
| 1044 | PLD3 | 2512 | LOC100653080 | 3980 | TCAP | 5448 | MKL1 | 6916 | UNK | 8384 | TNFRSF17 |
| 1045 | GABARAPL1 | 2513 | EML5 | 3981 | TBX5 | 5449 | SPX | 6917 | ITGBL1 | 8385 | VAPA |
| 1046 | SNRNP25 | 2514 | PRDM1 | 3982 | FAM22D | 5450 | CD1B | 6918 | FAM110B | 8386 | ARRDC4 |
| 1047 | SLC39A6 | 2515 | VWA5B2 | 3983 | ZNF683 | 5451 | COL21A1 | 6919 | NEK1 | 8387 | PDXDC2P |
| 1048 | C4ORF19 | 2516 | TDRD9 | 3984 | RSAD2 | 5452 | DKFZP434J0226 | 6920 | ZNF609 | 8388 | LUM |
| 1049 | RNF7 | 2517 | TRIP12 | 3985 | FOXP1 | 5453 | MCM6 | 6921 | FSIP2 | 8389 | KIF16B |
| 1050 | SFN | 2518 | FAM18B2-CDRT4 | 3986 | SCRN1 | 5454 | PNPT1 | 6922 | ATP7B | 8390 | SLC39A8 |
| 1051 | LRRC41 | 2519 | RNF151 | 3987 | MGC24103 | 5455 | CARD16 | 6923 | TIMM17B | 8391 | NR4A2 |
| 1052 | CEP76 | 2520 | DKFZP564C152 | 3988 | LTN1 | 5456 | ARHGAP35 | 6924 | LEPREL2 | 8392 | CMKLR1 |
| 1053 | RHOD | 2521 | OSBPL5 | 3989 | DISP1 | 5457 | USP47 | 6925 | FAM3B | 8393 | TRABD2A |
| 1054 | CDRT15 | 2522 | PRDM13 | 3990 | C10ORF53 | 5458 | FCRLA | 6926 | RIOK2 | 8394 | BHLHE22 |
| 1055 | SYT11 | 2523 | NBPF10 | 3991 | LOC643650 | 5459 | EPN2 | 6927 | RP1-154K9.2 | 8395 | TENM3 |
| 1056 | SWI5 | 2524 | CDRT1 | 3992 | ZBTB37 | 5460 | SNAPC4 | 6928 | CD101 | 8396 | TRAF3IP3 |
| 1057 | ZFAND6 | 2525 | EPB41L4A | 3993 | LRRC55 | 5461 | U2AF1 | 6929 | LOC101926987 | 8397 | KLK10 |
| 1058 | RPL18A | 2526 | HIST1H4B | 3994 | LY6G5B | 5462 | GSKIP | 6930 | RRAGC | 8398 | SLC1A3 |
| 1059 | STK19 | 2527 | OBSL1 | 3995 | LOC652276 | 5463 | SLC7A7 | 6931 | TTLL6 | 8399 | SERPINE2 |
| 1060 | HMGN2 | 2528 | HES5 | 3996 | TCEANC | 5464 | PCNXL4 | 6932 | FBF1 | 8400 | SRPX2 |
| 1061 | SLC25A20 | 2529 | SNORA59B | 3997 | LOC100509553 | 5465 | MFI2 | 6933 | LOC400794 | 8401 | FCMR |
| 1062 | GRINA | 2530 | KRTAP9-1 | 3998 | C12ORF51 | 5466 | ANKUB1 | 6934 | RAE1 | 8402 | KRT4 |
| 1063 | COG4 | 2531 | SERPING1 | 3999 | HOGA1 | 5467 | PPM1A | 6935 | NOL4 | 8403 | GZMK |
| 1064 | SETD7 | 2532 | SLAMF9 | 4000 | KLK7 | 5468 | CFLAR-AS1 | 6936 | TBC1D9B | 8404 | RARRES1 |
| 1065 | ZNF701 | 2533 | DCUN1D2 | 4001 | LOC100127951 | 5469 | DNAL1 | 6937 | SLC44A4 | 8405 | FBLN2 |
| 1066 | PI4K2A | 2534 | NRSN2 | 4002 | SPRR2F | 5470 | TSHR | 6938 | UPF3A | 8406 | CLMP |
| 1067 | SLC31A1 | 2535 | PWWP2A | 4003 | TSC2 | 5471 | LYRM2 | 6939 | COL11A1 | 8407 | SMOC2 |
| 1068 | MTCH1 | 2536 | HIST1H4D | 4004 | DIP2C | 5472 | PSME1 | 6940 | METTL18 | 8408 | SOX21 |
| 1069 | MDM1 | 2537 | LOC200609 | 4005 | MYB | 5473 | S100A6 | 6941 | ANKRD31 | 8409 | KIF27 |
| 1070 | TCF25 | 2538 | KLKB1 | 4006 | LOC283887 | 5474 | AGPAT4 | 6942 | NALCN | 8410 | CD5 |
| 1071 | LGALS9 | 2539 | FLJ31662 | 4007 | MGEA5 | 5475 | CR1L | 6943 | C17ORF51 | 8411 | EFCAB2 |
| 1072 | UBA2 | 2540 | LOC646719 | 4008 | ARHGEF38 | 5476 | KCTD17 | 6944 | HSPBP1 | 8412 | SAMD3 |
| 1073 | CORO1B | 2541 | DDX59 | 4009 | NMBR | 5477 | BRINP2 | 6945 | RAB3IL1 | 8413 | OAS3 |
| 1074 | GBAS | 2542 | APOBEC1 | 4010 | GATA2 | 5478 | GLTSCR1 | 6946 | MASP1 | 8414 | TRAT1 |
| 1075 | LONRF1 | 2543 | GOLGA6L9 | 4011 | NT5DC4 | 5479 | USP2 | 6947 | AIG1 | 8415 | CD84 |
| 1076 | GSTO1 | 2544 | MGC4859 | 4012 | ZNRF4 | 5480 | ANO9 | 6948 | 2-Mar | 8416 | FCGR2B |
| 1077 | TLR4 | 2545 | C2ORF54 | 4013 | SHROOM3 | 5481 | MYO1B | 6949 | COLCA2 | 8417 | SULF1 |
| 1078 | AP2A2 | 2546 | CSNK1G1 | 4014 | LOC100130015 | 5482 | RNU6-645P | 6950 | CLCNKB | 8418 | CD27 |
| 1079 | FCRL5 | 2547 | SYTL5 | 4015 | S100A7A | 5483 | TULP3 | 6951 | PLCD4 | 8419 | ASIP |
| 1080 | BTBD10 | 2548 | CTU1 | 4016 | TCL1B | 5484 | PHF13 | 6952 | CFL1 | 8420 | CILP |
| 1081 | HIF1A | 2549 | SP140 | 4017 | C1ORF220 | 5485 | MAP3K8 | 6953 | ZNF213 | 8421 | NSG1 |
| 1082 | HKDC1 | 2550 | MECP2 | 4018 | GJA8 | 5486 | RAP2C-AS1 | 6954 | COL4A2 | 8422 | PTGIS |
| 1083 | SLC38A2 | 2551 | SLED1 | 4019 | C22ORF34 | 5487 | MAX | 6955 | COL6A5 | 8423 | BTLA |
| 1084 | ATG3 | 2552 | SLC22A1 | 4020 | PNMT | 5488 | DYNLT1 | 6956 | MAD2L1 | 8424 | NAV3 |
| 1085 | NDUFV2 | 2553 | LOC100287314 | 4021 | ST5 | 5489 | FUT8 | 6957 | DCTN2 | 8425 | KLHL41 |
| 1086 | REM1 | 2554 | LOC286437 | 4022 | BICC1 | 5490 | SLFN13 | 6958 | AC007131.2 | 8426 | TLR10 |
| 1087 | TTC35 | 2555 | MYO5C | 4023 | SPTSSB | 5491 | WDR19 | 6959 | STRA6 | 8427 | PVRIG |
| 1088 | UGCG | 2556 | LOC401220 | 4024 | OTUD6A | 5492 | ST6GALNAC4 | 6960 | PADI3 | 8428 | PDE1A |
| 1089 | PPARGC1B | 2557 | NBPF3 | 4025 | ZNF280D | 5493 | JADE1 | 6961 | FAN1 | 8429 | TMEM267 |
| 1090 | PCDH20 | 2558 | GRID2IP | 4026 | C1ORF138 | 5494 | RAB15 | 6962 | NWD1 | 8430 | NPL |
| 1091 | TMEM208 | 2559 | SOS1 | 4027 | MAGEC1 | 5495 | GAREML | 6963 | MIR503HG | 8431 | GYPE |
| 1092 | SLC30A9 | 2560 | NFATC2 | 4028 | CRYGC | 5496 | NASP | 6964 | HEATR6 | 8432 | CBLN4 |
| 1093 | MAFG | 2561 | SNORD116-19 | 4029 | SYT15 | 5497 | MKI67 | 6965 | ZNF143 | 8433 | LRRC31 |
| 1094 | ABCB1 | 2562 | IFNA4 | 4030 | ARGLU1 | 5498 | ITGA8 | 6966 | PTK7 | 8434 | FCRL2 |
| 1095 | PCNA | 2563 | ZNF808 | 4031 | LMX1B | 5499 | AP4E1 | 6967 | ACOXL | 8435 | TMIGD3 |
| 1096 | ZNF24 | 2564 | CNPY1 | 4032 | LOC285178 | 5500 | TAF6L | 6968 | SLC6A5 | 8436 | CD40LG |
| 1097 | SH3GLB2 | 2565 | LOC283911 | 4033 | ARNTL | 5501 | BAALCOS | 6969 | HNRNPAB | 8437 | LRRC17 |
| 1098 | C8ORF40 | 2566 | LOC1720 | 4034 | LOC100131657 | 5502 | RGS4 | 6970 | DCUN1D5 | 8438 | SELL |
| 1099 | ADD3 | 2567 | DMGDH | 4035 | LOC678655 | 5503 | LEMD2 | 6971 | KIAA0226L | 8439 | TNFRSF13B |
| 1100 | CLIC1 | 2568 | FLJ10661 | 4036 | CENPT | 5504 | ANK1 | 6972 | GRIN3B | 8440 | PTGER1 |
| 1101 | EPHA8 | 2569 | DLK1 | 4037 | LOC729121 | 5505 | SRPK1 | 6973 | SORCS1 | 8441 | TMEM106A |
| 1102 | ZCCHC24 | 2570 | GRIN2C | 4038 | TRIM71 | 5506 | ATP6V1D | 6974 | PDCD11 | 8442 | FAM180A |
| 1103 | SCAND1 | 2571 | LOC642852 | 4039 | FER1L6-AS1 | 5507 | RP11-274J7.2 | 6975 | GDA | 8443 | LOXL1 |
| 1104 | C14ORF1 | 2572 | TMEM145 | 4040 | STAG2 | 5508 | LOC101929450 | 6976 | HNMT | 8444 | JSRP1 |
| 1105 | GSTA4 | 2573 | NRG4 | 4041 | PBLD | 5509 | LIMS1 | 6977 | SNX2 | 8445 | ACKR1 |
| 1106 | RNF113B | 2574 | TSEN2 | 4042 | KLRF1 | 5510 | VENTX | 6978 | PDGFRA | 8446 | SNAP25 |
| 1107 | C7ORF13 | 2575 | PAQR3 | 4043 | C14ORF159 | 5511 | CENPJ | 6979 | SYT8 | 8447 | CYP3A5 |
| 1108 | TMPRSS4 | 2576 | TTLL7 | 4044 | CYP2B6 | 5512 | NPIPA1 | 6980 | KNG1 | 8448 | HSD17B13 |
| 1109 | BCAT1 | 2577 | LDB2 | 4045 | ADAM20 | 5513 | PKD1P1 | 6981 | LOC101929719 | 8449 | GOLGA6B |
| 1110 | BCCIP | 2578 | PPP1R2P3 | 4046 | CXORF66 | 5514 | VPS13A | 6982 | ZMYM1 | 8450 | NPTX1 |
| 1111 | ACVR1 | 2579 | SLC29A4 | 4047 | HSPB11 | 5515 | ADAMTSL5 | 6983 | POLRMT | 8451 | CHRNA1 |
| 1112 | CCDC167 | 2580 | MACF1 | 4048 | SLC2A5 | 5516 | ANK2 | 6984 | C8B | 8452 | MIR2116 |
| 1113 | STIP1 | 2581 | LOC100127885 | 4049 | CTAG2 | 5517 | LRP2 | 6985 | TEX9 | 8453 | FDCSP |
| 1114 | LOC100128682 | 2582 | SLC36A3 | 4050 | LOC338579 | 5518 | MYBPC2 | 6986 | DNAJB4 | 8454 | SCGB3A2 |
| 1115 | SRPK2 | 2583 | DNMT3L | 4051 | PLA2G2C | 5519 | THBS3 | 6987 | TEX14 | 8455 | MGARP |
| 1116 | HPS1 | 2584 | NBEAL1 | 4052 | LOC158863 | 5520 | NUP50 | 6988 | LINC01094 | 8456 | C1S |
| 1117 | COX10 | 2585 | RASSF2 | 4053 | FREM1 | 5521 | TRIM24 | 6989 | OSBPL6 | 8457 | APLNR |
| 1118 | SERTAD3 | 2586 | YWHAE | 4054 | ABO | 5522 | AASDHPPT | 6990 | MAP2 | 8458 | TXNDC5 |
| 1119 | TPX2 | 2587 | LOC100130916 | 4055 | LOC253044 | 5523 | TAB1 | 6991 | WDR20 | 8459 | ADCYAP1 |
| 1120 | C6ORF145 | 2588 | ADARB1 | 4056 | PLB1 | 5524 | MEIOB | 6992 | NXF1 | 8460 | CXCL13 |
| 1121 | KCNMB2 | 2589 | DNAH17 | 4057 | CLCN6 | 5525 | HBP1 | 6993 | PTPRN2 | 8461 | SPATA7 |
| 1122 | PPPDE1 | 2590 | C1ORF86 | 4058 | FLJ25694 | 5526 | ADAMTS2 | 6994 | ZFP28 | 8462 | FAM181A |
| 1123 | DPP3 | 2591 | C19ORF29-AS1 | 4059 | APBA1 | 5527 | DNASE1L2 | 6995 | ZDHHC21 | 8463 | RPRML |
| 1124 | CRCP | 2592 | AFF1 | 4060 | C2ORF72 | 5528 | BTBD16 | 6996 | SNAI2 | 8464 | JCHAIN |
| 1125 | ENDOV | 2593 | LOC401357 | 4061 | LOC284454 | 5529 | CCL28 | 6997 | PTGER3 | 8465 | MOXD1 |
| 1126 | WBSCR16 | 2594 | LOC100652838 | 4062 | FOXD2 | 5530 | PKMYT1 | 6998 | RAP1GAP | 8466 | LAMP5 |
| 1127 | HSP90AB1 | 2595 | KLHDC7A | 4063 | DEFB128 | 5531 | LACC1 | 6999 | CHPF | 8467 | SFRP1 |
| 1128 | C1QL2 | 2596 | DLEU2L | 4064 | SRRM2 | 5532 | COMMD3 | 7000 | ELAVL2 | 8468 | MFF |
| 1129 | RAET1E | 2597 | TAF1 | 4065 | SNORA74A | 5533 | GCDH | 7001 | LOC285191 | 8469 | DRC3 |
| 1130 | PDIA6 | 2598 | GLYATL3 | 4066 | FAM115C | 5534 | TMEM134 | 7002 | CDK12 | 8470 | SAXO2 |
| 1131 | GABPB1 | 2599 | KDM5C | 4067 | KRTAP4-12 | 5535 | RBM39 | 7003 | CMAHP | 8471 | NPIPA5 |
| 1132 | DHX15 | 2600 | RPS6KA5 | 4068 | TINAGL1 | 5536 | LOC100132735 | 7004 | MSH2 | 8472 | EHF |
| 1133 | TRMT2A | 2601 | LOC100130924 | 4069 | MST152 | 5537 | ASAP1 | 7005 | WDR27 | 8473 | MIR7113 |
| 1134 | TMED5 | 2602 | SLITRK1 | 4070 | KRT79 | 5538 | TPCN2 | 7006 | KIAA1217 | 8474 | IFITM3 |
| 1135 | C21ORF119 | 2603 | TBC1D1 | 4071 | HOXA6 | 5539 | PMM1 | 7007 | WDR63 | 8475 | LOC101930306 |
| 1136 | NCF4 | 2604 | FOXRED2 | 4072 | RBM14 | 5540 | FMN2 | 7008 | KIF13B | 8476 | TAF13 |
| 1137 | DHRS7B | 2605 | MYH7B | 4073 | CACNA1B | 5541 | GCAT | 7009 | SMIM5 | 8477 | GATAD1 |
| 1138 | GOLGA4 | 2606 | HIST1H2AM | 4074 | CHRNA4 | 5542 | GPR126 | 7010 | ZNF521 | 8478 | SMG1P5 |
| 1139 | MYD88 | 2607 | KCNA4 | 4075 | F7 | 5543 | GUCY2GP | 7011 | DYNC2H1 | 8479 | UBXN2A |
| 1140 | CLN3 | 2608 | ST7-OT4 | 4076 | EBPL | 5544 | ZNF282 | 7012 | FBLN5 | 8480 | NLN |
| 1141 | SPSB1 | 2609 | ST8SIA3 | 4077 | LOC100132292 | 5545 | PKD1 | 7013 | CTPS1 | 8481 | LUC7L2 |
| 1142 | GRHPR | 2610 | LOC440297 | 4078 | SNORA81 | 5546 | OS9 | 7014 | C2CD2L | 8482 | SLC35E1 |
| 1143 | NIF3L1 | 2611 | XRRA1 | 4079 | LOC100130429 | 5547 | RASGRF2 | 7015 | FAM71E2 | 8483 | LINC00189 |
| 1144 | C15ORF29 | 2612 | SYTL4 | 4080 | TGFBR2 | 5548 | RRNAD1 | 7016 | SESN3 | 8484 | KTN1 |
| 1145 | NR2E3 | 2613 | CYP4Z1 | 4081 | RAP1GAP2 | 5549 | CDK10 | 7017 | CATSPERD | 8485 | SLX1B-SULT1A4 |
| 1146 | HSPB1 | 2614 | DDB1 | 4082 | CFHR5 | 5550 | CDH26 | 7018 | AZIN2 | 8486 | SRGAP2 |
| 1147 | KRT28 | 2615 | SETBP1 | 4083 | KAT6B | 5551 | C8ORF44 | 7019 | CEP95 | 8487 | CFDP1 |
| 1148 | FAM75C2 | 2616 | PTPRG | 4084 | WFDC6 | 5552 | ITFG2 | 7020 | SDCCAG8 | 8488 | IFITM2 |
| 1149 | DNAJC25 | 2617 | PLXNC1 | 4085 | RHCG | 5553 | RNF103 | 7021 | ZNF767P | 8489 | TRBC1 |
| 1150 | FAM57A | 2618 | GRIK5 | 4086 | SUSD2 | 5554 | CC2D2A | 7022 | PRDM12 | 8490 | RIOK3 |
| 1151 | SGPP2 | 2619 | IRGM | 4087 | DPRXP4 | 5555 | LOC284373 | 7023 | SPECC1L | 8491 | SLC22A3 |
| 1152 | TRAF3IP2 | 2620 | LOC389676 | 4088 | LOC643802 | 5556 | CLN5 | 7024 | DOCK10 | 8492 | IQSEC1 |
| 1153 | CCNDBP1 | 2621 | RTN4RL2 | 4089 | EFHC2 | 5557 | NUP107 | 7025 | SLC15A1 | 8493 | SULT1A1 |
| 1154 | ASPSCR1 | 2622 | VPS53 | 4090 | DPP6 | 5558 | ENPP3 | 7026 | TTYH1 | 8494 | SSR3 |
| 1155 | RPL7 | 2623 | ZNF43 | 4091 | LOC100129408 | 5559 | ACE2 | 7027 | ABCA4 | 8495 | MIR3916 |
| 1156 | MPG | 2624 | DLX4 | 4092 | LOC100128668 | 5560 | PDE11A | 7028 | AUH | 8496 | MORF4L2 |
| 1157 | SLC11A1 | 2625 | HYDIN | 4093 | FAM178B | 5561 | CPA2 | 7029 | AMOTL1 | 8497 | ASXL2 |
| 1158 | CLDN7 | 2626 | C19ORF69 | 4094 | TNKS1BP1 | 5562 | TTC12 | 7030 | SEZ6 | 8498 | MIR1304 |
| 1159 | LDLR | 2627 | RNPC3 | 4095 | ACTR3C | 5563 | TNFRSF19 | 7031 | COL24A1 | 8499 | LSS |
| 1160 | FGFR3 | 2628 | NOTCH2NL | 4096 | LOC606724 | 5564 | APOBEC3G | 7032 | EFNB2 | 8500 | PMEPA1 |
| 1161 | PLEKHM2 | 2629 | TLK2 | 4097 | LOC646851 | 5565 | GYLTL1B | 7033 | LOC101928882 | 8501 | MPV17L |
| 1162 | SLMO2 | 2630 | VWA5B1 | 4098 | S100A7L2 | 5566 | PPL | 7034 | CEP192 | 8502 | SLC8A1 |
| 1163 | C7ORF60 | 2631 | RBM41 | 4099 | SCARNA5 | 5567 | LEMD3 | 7035 | ZNF365 | 8503 | APOBEC3F |
| 1164 | RPAIN | 2632 | ANAPC1 | 4100 | FLJ42351 | 5568 | DMD | 7036 | CIPC | 8504 | LPP |
| 1165 | MAPK13 | 2633 | TPM3 | 4101 | SNORA53 | 5569 | ALPK2 | 7037 | HECW2 | 8505 | NDRG3 |
| 1166 | C17ORF58 | 2634 | GRK1 | 4102 | PRO0471 | 5570 | SGPL1 | 7038 | PPY | 8506 | IFNGR1 |
| 1167 | TMEM41A | 2635 | PRIC285 | 4103 | LOC100129275 | 5571 | HSD11B1 | 7039 | WDR38 | 8507 | SIGLEC1 |
| 1168 | RTN1 | 2636 | ARIH1 | 4104 | FHL1 | 5572 | NEK5 | 7040 | SLC46A3 | 8508 | ANKRD28 |
| 1169 | IMPAD1 | 2637 | CELF4 | 4105 | ACBD7 | 5573 | MUC1 | 7041 | FRMD6 | 8509 | LOC100996506 |
| 1170 | CBR3 | 2638 | RCC1 | 4106 | LOC730755 | 5574 | CHEK1 | 7042 | TXNDC15 | 8510 | STARD10 |
| 1171 | TXNL4A | 2639 | WWP2 | 4107 | MERTK | 5575 | SCNN1A | 7043 | HELQ | 8511 | GGNBP2 |
| 1172 | WDR25 | 2640 | SNORD124 | 4108 | LOC283335 | 5576 | COL4A2-AS2 | 7044 | CKMT1B | 8512 | FNBP1 |
| 1173 | TRPV4 | 2641 | NCKAP5 | 4109 | FLJ35424 | 5577 | IRAK3 | 7045 | POLR1E | 8513 | MYLIP |
| 1174 | NSA2 | 2642 | NDST4 | 4110 | LOC100128401 | 5578 | NRG3 | 7046 | MNDA | 8514 | RASA4B |
| 1175 | SERPINF2 | 2643 | ATM | 4111 | CASC2 | 5579 | RTEL1 | 7047 | SV2B | 8515 | MSANTD3-TMEFF1 |
| 1176 | RASGRF1 | 2644 | MRGPRX4 | 4112 | ZYG11A | 5580 | CYP2R1 | 7048 | SPDYE4 | 8516 | SLC11A2 |
| 1177 | ZNF295 | 2645 | SLC2A6 | 4113 | GSTTP2 | 5581 | CBFA2T3 | 7049 | COL13A1 | 8517 | REV3L |
| 1178 | NCOA7 | 2646 | METTL10 | 4114 | MTR | 5582 | CPSF7 | 7050 | CCDC17 | 8518 | H2AFY |
| 1179 | C22ORF28 | 2647 | SLC7A10 | 4115 | MIRLET7BHG | 5583 | IGF2BP1 | 7051 | DBP | 8519 | LINC00607 |
| 1180 | HMGCR | 2648 | OVOS | 4116 | DOCK9 | 5584 | DDX25 | 7052 | CROCC | 8520 | ADAM9 |
| 1181 | RPL3 | 2649 | PHACTR2 | 4117 | KCNE1 | 5585 | GRM7 | 7053 | LRWD1 | 8521 | GMPR |
| 1182 | EIF3I | 2650 | IKZF2 | 4118 | LOC151657 | 5586 | TCTN1 | 7054 | DDX26B | 8522 | CSGALNACT2 |
| 1183 | RPL29 | 2651 | RASSF6 | 4119 | GRM2 | 5587 | ARFGAP3 | 7055 | TRIM55 | 8523 | L1TD1 |
| 1184 | IDH1 | 2652 | MAS1L | 4120 | VSTM1 | 5588 | SMAP1 | 7056 | SLC16A12 | 8524 | AMFR |
| 1185 | CATSPERB | 2653 | FAM178A | 4121 | ASPG | 5589 | C1ORF85 | 7057 | PLS3 | 8525 | OSM |
| 1186 | ZBTB34 | 2654 | SRSF11 | 4122 | FGF14-IT1 | 5590 | ACSM5 | 7058 | PRSS36 | 8526 | LOC101929612 |
| 1187 | NUFIP1 | 2655 | DSP | 4123 | GPR182 | 5591 | MYH3 | 7059 | EPHX2 | 8527 | RPS27 |
| 1188 | CDK5RAP1 | 2656 | LINC00111 | 4124 | FLJ27502 | 5592 | WDTC1 | 7060 | KMT2A | 8528 | MX1 |
| 1189 | HIATL1 | 2657 | LINC00221 | 4125 | LOC100507073 | 5593 | ARHGAP44 | 7061 | LINC00331 | 8529 | PECAM1 |
| 1190 | TP53I11 | 2658 | XCR1 | 4126 | BAALC | 5594 | ZNF598 | 7062 | CRY2 | 8530 | TRIO |
| 1191 | RPSAP52 | 2659 | KIAA1530 | 4127 | TRPV1 | 5595 | KEAP1 | 7063 | WFS1 | 8531 | FN1 |
| 1192 | CTSS | 2660 | ETV3 | 4128 | KALRN | 5596 | SCN1B | 7064 | TUBGCP4 | 8532 | ALOX5 |
| 1193 | CRTAC1 | 2661 | LOC100128348 | 4129 | KIAA0087 | 5597 | LOC642533 | 7065 | TRIM26 | 8533 | SLC39A13 |
| 1194 | FAS | 2662 | BTBD9 | 4130 | LOC100133131 | 5598 | MS4A6A | 7066 | SIDT1 | 8534 | SMARCA4 |
| 1195 | ARF4 | 2663 | GPR125 | 4131 | PRINS | 5599 | MED23 | 7067 | UBE2D4 | 8535 | SNX20 |
| 1196 | CCDC141 | 2664 | LOC100131000 | 4132 | FLJ38717 | 5600 | KATNAL1 | 7068 | CPT1B | 8536 | CLEC4A |
| 1197 | NAA20 | 2665 | C6ORF221 | 4133 | SNORA28 | 5601 | SDC3 | 7069 | ABCA17P | 8537 | NRDC |
| 1198 | MRPL17 | 2666 | MGC34796 | 4134 | LOC100131864 | 5602 | ECHDC1 | 7070 | ZNF576 | 8538 | CSF1R |
| 1199 | ANXA5 | 2667 | NFASC | 4135 | RPH3A | 5603 | ANXA3 | 7071 | TAT | 8539 | VASH1 |
| 1200 | CCT4 | 2668 | LOC729696 | 4136 | LDLRAD3 | 5604 | ATP13A3 | 7072 | FAM208B | 8540 | ALOX15B |
| 1201 | PPP1R14C | 2669 | CYP19A1 | 4137 | VPS18 | 5605 | STX4 | 7073 | GRAMD1C | 8541 | PSMD5-AS1 |
| 1202 | BARHL2 | 2670 | TRIM61 | 4138 | LINC00301 | 5606 | PPP2R5B | 7074 | TDRKH | 8542 | SDHAP2 |
| 1203 | TRIB1 | 2671 | FAM205B | 4139 | ALX4 | 5607 | SNHG11 | 7075 | SPAG17 | 8543 | HECTD1 |
| 1204 | USP22 | 2672 | GLB1L3 | 4140 | TTC24 | 5608 | NID1 | 7076 | CHRM3 | 8544 | SCAF4 |
| 1205 | SLC27A4 | 2673 | FIBCD1 | 4141 | LOC100129869 | 5609 | FAP | 7077 | MTG1 | 8545 | AOAH |
| 1206 | MEGF9 | 2674 | DNAH10 | 4142 | LOC393078 | 5610 | CCL15-CCL14 | 7078 | ACAT1 | 8546 | EPSTI1 |
| 1207 | FBLIM1 | 2675 | APC2 | 4143 | SLC5A11 | 5611 | CATIP-AS1 | 7079 | AP3B2 | 8547 | SULT1C2 |
| 1208 | COX15 | 2676 | C18ORF42 | 4144 | ZNF682 | 5612 | LOC101927896 | 7080 | ATF6B | 8548 | RTN2 |
| 1209 | SCRN2 | 2677 | LTA4H | 4145 | SOX3 | 5613 | ATP6V1C2 | 7081 | NXF2B | 8549 | LAMP1 |
| 1210 | RAD1 | 2678 | LOC100507050 | 4146 | CYP2G1P | 5614 | AMPD1 | 7082 | ANKMY1 | 8550 | MSH6 |
| 1211 | ABCA3 | 2679 | NQO2 | 4147 | NEK7 | 5615 | POPDC2 | 7083 | CEP162 | 8551 | ZCCHC7 |
| 1212 | EXOC8 | 2680 | HAUS5 | 4148 | C1ORF227 | 5616 | AGBL1 | 7084 | BOC | 8552 | YME1L1 |
| 1213 | CDC37 | 2681 | RUNX2 | 4149 | SPRR2C | 5617 | DTNA | 7085 | PAX6 | 8553 | LOC102723773 |
| 1214 | ERI2 | 2682 | LOC100132481 | 4150 | CEP120 | 5618 | KATNAL2 | 7086 | MOV10L1 | 8554 | SLC22A4 |
| 1215 | ENSA | 2683 | XKR4 | 4151 | NEBL | 5619 | ZSWIM3 | 7087 | GFI1 | 8555 | AFAP1L1 |
| 1216 | SEC13 | 2684 | LYG2 | 4152 | LOC100131826 | 5620 | BACH1-IT3 | 7088 | PAX5 | 8556 | LINS1 |
| 1217 | IL32 | 2685 | CENPC1 | 4153 | METTL21A | 5621 | CDS2 | 7089 | PIGQ | 8557 | MED31 |
| 1218 | MIA3 | 2686 | AKT2 | 4154 | PEG3 | 5622 | C12ORF79 | 7090 | TRANK1 | 8558 | ACBD3 |
| 1219 | TMEM97 | 2687 | MGC16075 | 4155 | GABBR1 | 5623 | FAM197Y2 | 7091 | RP11-128P17.2 | 8559 | SPINT1 |
| 1220 | NOP16 | 2688 | UBE3A | 4156 | SHC2 | 5624 | LRP5L | 7092 | NCOA4 | 8560 | ZMYM2 |
| 1221 | PUS1 | 2689 | CCDC57 | 4157 | TRAPPC2 | 5625 | RBBP8 | 7093 | ALDH18A1 | 8561 | GPR84 |
| 1222 | CSTB | 2690 | KIAA1751 | 4158 | A1CF | 5626 | CLCN7 | 7094 | TMEM63C | 8562 | ZMYND15 |
| 1223 | MED12 | 2691 | LOC402779 | 4159 | PSORS1C1 | 5627 | CNTN2 | 7095 | UNC93B1 | 8563 | SNORD50B |
| 1224 | CPSF1 | 2692 | LAMA5 | 4160 | PLK5 | 5628 | GLI1 | 7096 | MAP7D3 | 8564 | HLA-DRB4 |
| 1225 | TCF21 | 2693 | UBE2CBP | 4161 | SNORD17 | 5629 | GAB4 | 7097 | FAM3A | 8565 | GNB4 |
| 1226 | PAQR4 | 2694 | CYP4A11 | 4162 | TERF1 | 5630 | RPL37A | 7098 | LCT | 8566 | ETV6 |
| 1227 | LOC148709 | 2695 | TBC1D10C | 4163 | LOC340515 | 5631 | PIK3R5 | 7099 | TLL2 | 8567 | LOC101060747 |
| 1228 | ADAMTS3 | 2696 | GPR20 | 4164 | SLCO1A2 | 5632 | DPEP3 | 7100 | GDAP2 | 8568 | CEPT1 |
| 1229 | NCKAP1 | 2697 | ZNF419 | 4165 | LOC340178 | 5633 | ARHGAP4 | 7101 | ARHGEF16 | 8569 | KLF4 |
| 1230 | CKMT2 | 2698 | TXNL1 | 4166 | ACSBG1 | 5634 | LSMEM2 | 7102 | NELFE | 8570 | CBX4 |
| 1231 | MMP19 | 2699 | FLJ23867 | 4167 | SPEN | 5635 | SPATA21 | 7103 | MPO | 8571 | KIAA0907 |
| 1232 | C11ORF95 | 2700 | CYP4F2 | 4168 | ST6GAL1 | 5636 | SIGLEC15 | 7104 | MEGF10 | 8572 | FRG1JP |
| 1233 | GABARAP | 2701 | KIAA1429 | 4169 | C16ORF73 | 5637 | CALCR | 7105 | NFS1 | 8573 | PGM5 |
| 1234 | GMPR2 | 2702 | JMJD7-PLA2G4B | 4170 | LRRC2 | 5638 | SMG6 | 7106 | DAW1 | 8574 | TRRAP |
| 1235 | ANKRD5 | 2703 | LOC100133920 | 4171 | PPP6R1 | 5639 | ILVBL | 7107 | SLC29A2 | 8575 | SOAT1 |
| 1236 | C20ORF160 | 2704 | FLJ39639 | 4172 | GPR179 | 5640 | LOC101928248 | 7108 | C5 | 8576 | SECISBP2 |
| 1237 | SPOCK1 | 2705 | NCAM1 | 4173 | NDUFS7 | 5641 | ESCO2 | 7109 | TK1 | 8577 | UNC13D |
| 1238 | PHF20 | 2706 | MBTD1 | 4174 | CD86 | 5642 | DAB2IP | 7110 | STK31 | 8578 | MR1 |
| 1239 | ENOPH1 | 2707 | TET1 | 4175 | FAT2 | 5643 | SLC22A11 | 7111 | AGRP | 8579 | LOC100129406 |
| 1240 | RFNG | 2708 | HCG18 | 4176 | HMBOX1 | 5644 | 1-Mar | 7112 | PCOLCE2 | 8580 | IFIT2 |
| 1241 | GPR108 | 2709 | SYT2 | 4177 | XIST | 5645 | TMEM255B | 7113 | LOC151484 | 8581 | BAG5 |
| 1242 | LAP3 | 2710 | GRAMD2 | 4178 | SERPINB2 | 5646 | NUP35 | 7114 | ZNF512B | 8582 | CDC42-IT1 |
| 1243 | LRPPRC | 2711 | PRSS8 | 4179 | MMP12 | 5647 | NAALAD2 | 7115 | PUM1 | 8583 | TCEB1 |
| 1244 | FGF8 | 2712 | PLAC4 | 4180 | SIPA1L1 | 5648 | SLC5A3 | 7116 | SNRPF | 8584 | BAZ1A |
| 1245 | CGGBP1 | 2713 | UNC5C | 4181 | CCL24 | 5649 | FAM213A | 7117 | NYX | 8585 | PELI1 |
| 1246 | BMI1 | 2714 | SNAR-E | 4182 | ATP8B4 | 5650 | MAP3K14 | 7118 | CCDC101 | 8586 | TACSTD2 |
| 1247 | OTUD1 | 2715 | CC2D2B | 4183 | STAB1 | 5651 | ARNTL2 | 7119 | USP11 | 8587 | SNHG17 |
| 1248 | EIF4G2 | 2716 | FAM90A10 | 4184 | PDE4D | 5652 | MAD2L2 | 7120 | LLGL1 | 8588 | LGALS2 |
| 1249 | SPTLC1 | 2717 | GLYCTK | 4185 | SMOX | 5653 | CASP2 | 7121 | INSC | 8589 | ESYT2 |
| 1250 | SLC1A4 | 2718 | LEPROTL1 | 4186 | MMP9 | 5654 | TSGA13 | 7122 | MOCOS | 8590 | MINOS1P1 |
| 1251 | SLC22A18AS | 2719 | CDKAL1 | 4187 | ZMIZ1-AS1 | 5655 | AGO2 | 7123 | CHI3L2 | 8591 | MYO9B |
| 1252 | LCMT2 | 2720 | LOC100132111 | 4188 | ADAMDEC1 | 5656 | FMO2 | 7124 | AKT3 | 8592 | EIF5B |
| 1253 | SPNS1 | 2721 | C8ORF60 | 4189 | SRGAP1 | 5657 | ANKRD24 | 7125 | ACSF2 | 8593 | PSIP1 |
| 1254 | KRT18P55 | 2722 | LOC641467 | 4190 | TIMP3 | 5658 | BTBD11 | 7126 | WHAMMP3 | 8594 | TVP23B |
| 1255 | TNFSF13 | 2723 | ZBTB20 | 4191 | CRLF2 | 5659 | CNR2 | 7127 | RPS6KB2 | 8595 | STEAP3 |
| 1256 | ZFPM2 | 2724 | FOSL2 | 4192 | SPINK1 | 5660 | THPO | 7128 | AKAP7 | 8596 | AFF4 |
| 1257 | C3 | 2725 | IGF2BP2 | 4193 | MMP2 | 5661 | GRB14 | 7129 | DAGLB | 8597 | JMJD1C |
| 1258 | GTF2E1 | 2726 | CCDC102B | 4194 | FLT1 | 5662 | NDRG4 | 7130 | ANO7 | 8598 | HLA-F-AS1 |
| 1259 | SH3YL1 | 2727 | HOMER2 | 4195 | LOC100132891 | 5663 | RPS19BP1 | 7131 | MME | 8599 | TGM2 |
| 1260 | TUBB2A | 2728 | LOC389834 | 4196 | MSC | 5664 | PDP1 | 7132 | CYP4F8 | 8600 | RASSF5 |
| 1261 | PSMB5 | 2729 | PARK2 | 4197 | MET | 5665 | MREG | 7133 | LIG1 | 8601 | WNT5A |
| 1262 | MRPL37 | 2730 | LOC401400 | 4198 | ADAM28 | 5666 | LCK | 7134 | MYCBPAP | 8602 | RIN3 |
| 1263 | ASNA1 | 2731 | RAB43P1 | 4199 | BRWD3 | 5667 | NUP210 | 7135 | MAP4K2 | 8603 | SLC43A2 |
| 1264 | OTUB1 | 2732 | C17ORF80 | 4200 | IL1R1 | 5668 | CYB561D2 | 7136 | S100PBP | 8604 | HSPA1B |
| 1265 | NIM1 | 2733 | TBX3 | 4201 | CCL2 | 5669 | TMEM51 | 7137 | MYL7 | 8605 | SCARB1 |
| 1266 | IRAK2 | 2734 | LOC203274 | 4202 | DYSF | 5670 | TOMM40L | 7138 | ZNF324 | 8606 | LOC100134445 |
| 1267 | PARM1 | 2735 | C4ORF42 | 4203 | MAU2 | 5671 | PSMC2 | 7139 | MAGI3 | 8607 | CYSLTR1 |
| 1268 | BTBD3 | 2736 | LOC100131551 | 4204 | GRASP | 5672 | TAF8 | 7140 | NEK10 | 8608 | C12ORF66 |
| 1269 | ORAI2 | 2737 | LOC283392 | 4205 | MATK | 5673 | DYNLRB2 | 7141 | SSX2IP | 8609 | GADD45A |
| 1270 | TNP1 | 2738 | LOC100507199 | 4206 | IL1RL1 | 5674 | INPP5A | 7142 | NVL | 8610 | DIXDC1 |
| 1271 | ITGA6 | 2739 | CASS4 | 4207 | CAMKK2 | 5675 | CCDC149 | 7143 | HOXB6 | 8611 | KANSL1 |
| 1272 | FES | 2740 | KRTAP5-10 | 4208 | INSIG1 | 5676 | ATRIP | 7144 | FAM19A3 | 8612 | RP2 |
| 1273 | UBE2E4P | 2741 | LOC399744 | 4209 | GATM | 5677 | RP11-715C4.1 | 7145 | FAM135B | 8613 | NUP58 |
| 1274 | OSTC | 2742 | CTNS | 4210 | DOCK4 | 5678 | CTNNA1 | 7146 | ABT1 | 8614 | PDLIM7 |
| 1275 | INO80C | 2743 | NR2C2 | 4211 | SRPX | 5679 | KIAA1468 | 7147 | NOVA1-AS1 | 8615 | TMEM173 |
| 1276 | TMED10 | 2744 | ZCWPW1 | 4212 | SLC44A1 | 5680 | SLC48A1 | 7148 | RHBDL2 | 8616 | PKM |
| 1277 | CHIC2 | 2745 | ZADH2 | 4213 | SULF2 | 5681 | NSMAF | 7149 | ST8SIA1 | 8617 | ARHGDIB |
| 1278 | UBAP1 | 2746 | EGFR | 4214 | CD109 | 5682 | SGCE | 7150 | FAM107A | 8618 | KRT10 |
| 1279 | POLR3K | 2747 | MGC16121 | 4215 | CSF2RA | 5683 | CDHR2 | 7151 | PKD1L1 | 8619 | HMG20B |
| 1280 | DDX5 | 2748 | ABCC13 | 4216 | CSF1 | 5684 | SMAD7 | 7152 | ATP5C1 | 8620 | ZNF638-IT1 |
| 1281 | LOC728739 | 2749 | RASSF4 | 4217 | LOC102724833 | 5685 | ABI2 | 7153 | CES4A | 8621 | CDC42 |
| 1282 | SMAD5-AS1 | 2750 | CCDC144A | 4218 | SCIN | 5686 | RNF216P1 | 7154 | GPR64 | 8622 | ZAK |
| 1283 | PDE4B | 2751 | CYB561D1 | 4219 | SLC7A11-AS1 | 5687 | PARK7 | 7155 | PRKG1 | 8623 | IRF5 |
| 1284 | CRYZ | 2752 | OBSCN | 4220 | MST4 | 5688 | SLC12A2 | 7156 | EML1 | 8624 | PTGR1 |
| 1285 | AP4S1 | 2753 | LOC100131283 | 4221 | FBXL13 | 5689 | PRELID1 | 7157 | ARAP2 | 8625 | PON2 |
| 1286 | PRKRIR | 2754 | HIST1H3H | 4222 | JARID2 | 5690 | KIAA1804 | 7158 | MYH13 | 8626 | DLEU2 |
| 1287 | CIB1 | 2755 | AGXT2L2 | 4223 | GCLM | 5691 | H2AFV | 7159 | PLEKHG5 | 8627 | LOC114224 |
| 1288 | KDM5B | 2756 | ZNF532 | 4224 | CD226 | 5692 | VDR | 7160 | PMM2 | 8628 | ADGRE1 |
| 1289 | OR1L1 | 2757 | MICU1 | 4225 | ETV5 | 5693 | TMPRSS2 | 7161 | PNCK | 8629 | CTH |
| 1290 | ERVI-1 | 2758 | PIAS1 | 4226 | EVC | 5694 | LOC101929089 | 7162 | LOC102723562 | 8630 | ZFP36L2 |
| 1291 | ATP6V1E1 | 2759 | OSR2 | 4227 | FMN1 | 5695 | ZNF79 | 7163 | RGS6 | 8631 | LOC101929819 |
| 1292 | HIVEP1 | 2760 | ZNF814 | 4228 | TBC1D30 | 5696 | TBC1D3B | 7164 | LOXL3 | 8632 | ABCG1 |
| 1293 | SMC3 | 2761 | LYZL1 | 4229 | CXXC1 | 5697 | CYP4F22 | 7165 | MYOM1 | 8633 | UBE2D1 |
| 1294 | KIAA0664 | 2762 | ZC3H10 | 4230 | ARL4C | 5698 | KLHL17 | 7166 | SYVN1 | 8634 | SORT1 |
| 1295 | PPIB | 2763 | BRD7 | 4231 | NCR3LG1 | 5699 | ZSWIM8-AS1 | 7167 | PPHLN1 | 8635 | TLR8 |
| 1296 | NUDT5 | 2764 | HIST2H2AC | 4232 | TRAF5 | 5700 | NOX3 | 7168 | CD6 | 8636 | PPBP |
| 1297 | FOXP4 | 2765 | C17ORF57 | 4233 | ATP6V0D2 | 5701 | PPFIA4 | 7169 | KLHL29 | 8637 | JAK1 |
| 1298 | SLC39A1 | 2766 | PBX1 | 4234 | USP20 | 5702 | CIDEB | 7170 | SLX4 | 8638 | TAF15 |
| 1299 | SLC16A14 | 2767 | LOC440173 | 4235 | ABCA12 | 5703 | DIP2A | 7171 | PTH1R | 8639 | SRC |
| 1300 | RABGGTB | 2768 | OR1F2P | 4236 | SLC2A1 | 5704 | SPEF2 | 7172 | MYO15A | 8640 | LOC101930416 |
| 1301 | SAMD5 | 2769 | OAS2 | 4237 | RFTN1 | 5705 | ALKBH6 | 7173 | ERCC1 | 8641 | PRNP |
| 1302 | CAV1 | 2770 | COL19A1 | 4238 | ITGB3 | 5706 | GALNT8 | 7174 | SLC12A1 | 8642 | GSTM3 |
| 1303 | LRRC23 | 2771 | LOC100132247 | 4239 | PIK3R6 | 5707 | CHD1L | 7175 | GPR116 | 8643 | MLLT10 |
| 1304 | COCH | 2772 | LMF1 | 4240 | SDC2 | 5708 | ACKR4 | 7176 | TARSL2 | 8644 | SIRPA |
| 1305 | MB21D2 | 2773 | PABPC1L | 4241 | TROVE2 | 5709 | GRIK1-AS2 | 7177 | COL2A1 | 8645 | VMO1 |
| 1306 | PCSK2 | 2774 | FLYWCH1 | 4242 | SEMA6B | 5710 | ARHGAP33 | 7178 | GNG12 | 8646 | ARHGEF11 |
| 1307 | ESYT1 | 2775 | LOC145837 | 4243 | ADCY3 | 5711 | LMBR1L | 7179 | BLACE | 8647 | NPIPB5 |
| 1308 | RAB18 | 2776 | BBS5 | 4244 | WDHD1 | 5712 | LOC101929926 | 7180 | C16ORF92 | 8648 | MIR4691 |
| 1309 | PSMA7 | 2777 | MIR4500HG | 4245 | ZNF410 | 5713 | CDHR1 | 7181 | ACADL | 8649 | TIAF1 |
| 1310 | MTFP1 | 2778 | GRIN2A | 4246 | CELSR1 | 5714 | CYP27A1 | 7182 | HIF1AN | 8650 | RRN3P1 |
| 1311 | CCDC72 | 2779 | CXORF1 | 4247 | CLEC5A | 5715 | RP11-148O21.2 | 7183 | LRRC49 | 8651 | SLX1A-SULT1A3 |
| 1312 | FBXL5 | 2780 | SCN2B | 4248 | THBS1 | 5716 | CENPN | 7184 | MOGAT1 | 8652 | SNORD5 |
| 1313 | PPAP2B | 2781 | CADM2 | 4249 | TBC1D8 | 5717 | LGR5 | 7185 | OSBPL7 | 8653 | IFITM1 |
| 1314 | UBXN6 | 2782 | KANK4 | 4250 | ABHD4 | 5718 | PIM1 | 7186 | PTPRU | 8654 | RASA4CP |
| 1315 | ASAP2 | 2783 | TMIGD2 | 4251 | SPTBN1 | 5719 | KPNB1 | 7187 | EFCAB1 | 8655 | TMEFF1 |
| 1316 | KIAA0090 | 2784 | MAGEB2 | 4252 | RASAL1 | 5720 | PCYOX1L | 7188 | SMYD5 | 8656 | COX3 |
| 1317 | TMEM87B | 2785 | TMEM179 | 4253 | EPHB2 | 5721 | RASGRP4 | 7189 | CA7 | 8657 | SDHAP1 |
| 1318 | MEX3C | 2786 | NPTN | 4254 | CSGALNACT1 | 5722 | ZNF670-ZNF695 | 7190 | WDR37 | 8658 | LOC101060604 |
| 1319 | TBXAS1 | 2787 | CAP2 | 4255 | IL3RA | 5723 | NAA40 | 7191 | APBB2 | 8659 | SNHG5 |
| 1320 | C20ORF108 | 2788 | ADAM12 | 4256 | MMP1 | 5724 | CD177 | 7192 | NCBP1 | 8660 | PDPK1 |
| 1321 | COQ6 | 2789 | C19ORF68 | 4257 | DFNB31 | 5725 | MED15 | 7193 | ABCB6 | 8661 | TMED7 |
| 1322 | PIK3CD | 2790 | GIT1 | 4258 | EMP2 | 5726 | PTPRQ | 7194 | PTER | 8662 | LOC100133331 |
| 1323 | SGK1 | 2791 | ORC2 | 4259 | ATAD2 | 5727 | LINC00661 | 7195 | TACC3 | 8663 | LOC101929792 |
| 1324 | ATP6V1H | 2792 | FBXL19-AS1 | 4260 | SLC28A3 | 5728 | BBS9 | 7196 | EBF2 | 8664 | LOC613037 |
| 1325 | C19ORF50 | 2793 | SYNE1 | 4261 | FADS1 | 5729 | MTMR8 | 7197 | SERPINI2 | 8665 | NDUFS8 |
| 1326 | DIRAS3 | 2794 | BRWD1-IT2 | 4262 | APBB1 | 5730 | E2F3 | 7198 | UBXN11 | 8666 | SULT1A4 |
| 1327 | DEK | 2795 | LOC400684 | 4263 | PALM2-AKAP2 | 5731 | ABCB11 | 7199 | LY75-CD302 | 8667 | LOC220729 |
| 1328 | CDR2 | 2796 | INSL3 | 4264 | BLM | 5732 | AQP11 | 7200 | ACRBP | 8668 | LOC101060596 |
| 1329 | CD81 | 2797 | LOC154872 | 4265 | HERC4 | 5733 | IQCK | 7201 | SORD | 8669 | LOC100133182 |
| 1330 | YIPF5 | 2798 | C19ORF55 | 4266 | ITGB7 | 5734 | TSPAN32 | 7202 | JMJD7 | 8670 | NPIPB4 |
| 1331 | KNTC1 | 2799 | LOC100130000 | 4267 | NT5DC2 | 5735 | RP11-292D4.1 | 7203 | C17ORF77 | 8671 | SULT1A3 |
| 1332 | RPL19P12 | 2800 | KRTAP9-7 | 4268 | NFX1 | 5736 | CLIC3 | 7204 | QRICH1 | 8672 | SNORA40 |
| 1333 | AHR | 2801 | CEP128 | 4269 | ANTXR2 | 5737 | NCAPH | 7205 | XRN2 | 8673 | LOC101060386 |
| 1334 | TIPARP | 2802 | SMCR7L | 4270 | LILRA3 | 5738 | CASC1 | 7206 | LPAR3 | 8674 | LINC01001 |
| 1335 | CCNA2 | 2803 | LFNG | 4271 | TMEM206 | 5739 | LOC101929947 | 7207 | SLC4A10 | 8675 | NPIPB3 |
| 1336 | NUDT2 | 2804 | LOC100130642 | 4272 | RSRP1 | 5740 | ABI3BP | 7208 | ERICH6B | 8676 | SNORA18 |
| 1337 | VAMP5 | 2805 | LOC100130278 | 4273 | LOC101929587 | 5741 | DGCR14 | 7209 | MORN1 | 8677 | SMG1P7 |
| 1338 | FNDC5 | 2806 | FRY | 4274 | GRIK4 | 5742 | TCP11L2 | 7210 | GTF2A2 | 8678 | LOC100132062 |
| 1339 | EIF2B5 | 2807 | NPIP | 4275 | ZFYVE16 | 5743 | PHYKPL | 7211 | APOC1 | 8679 | SNORA1 |
| 1340 | PHGDH | 2808 | LOC256483 | 4276 | STRIP2 | 5744 | ABCA5 | 7212 | PPP1R21 | 8680 | SNORA8 |
| 1341 | C2ORF55 | 2809 | PMS2 | 4277 | CLEC2D | 5745 | ENOX1 | 7213 | HORMAD1 | 8681 | LINC01000 |
| 1342 | MEF2BNB | 2810 | GJA9 | 4278 | PDE2A | 5746 | SLC25A17 | 7214 | TFDP2 | 8682 | LINC01002 |
| 1343 | CD164 | 2811 | LOC100271836 | 4279 | LINC01010 | 5747 | IL11RA | 7215 | ANAPC7 | 8683 | SMG1P1 |
| 1344 | SCUBE2 | 2812 | MUC12 | 4280 | RP11-452H21.1 | 5748 | IZUMO4 | 7216 | KIF1A | 8684 | BOLA2 |
| 1345 | F10 | 2813 | C17ORF39 | 4281 | RB1CC1 | 5749 | PTS | 7217 | ABCC12 | 8685 | SMG1P2 |
| 1346 | DNAJC19 | 2814 | MYH6 | 4282 | SYN1 | 5750 | GUCY2EP | 7218 | FERMT2 | 8686 | SLC7A5P1 |
| 1347 | TRAPPC1 | 2815 | HYDIN2 | 4283 | CD44 | 5751 | BRINP3 | 7219 | MED24 | 8687 | MIR3658 |
| 1348 | TBRG1 | 2816 | C5ORF42 | 4284 | XYLT1 | 5752 | PIP5K1C | 7220 | MID2 | 8688 | FBXO15 |
| 1349 | SCD | 2817 | CRLF3 | 4285 | ARAP1 | 5753 | CLCN5 | 7221 | MEG3 | 8689 | PROCR |
| 1350 | GAS6 | 2818 | INO80D | 4286 | PRKCA | 5754 | ADAM8 | 7222 | CDC5L | 8690 | GLDN |
| 1351 | TMEM47 | 2819 | CHTOP | 4287 | MYO5B | 5755 | SIGLEC9 | 7223 | ITIH5 | 8691 | CD3D |
| 1352 | TMEM62 | 2820 | GPR3 | 4288 | FLNB | 5756 | IREB2 | 7224 | YAF2 | 8692 | TPD52 |
| 1353 | EXOSC7 | 2821 | RPPH1 | 4289 | DNASE1L3 | 5757 | TTYH2 | 7225 | IFI27 | 8693 | PDPN |
| 1354 | CLCN2 | 2822 | INE1 | 4290 | RNF121 | 5758 | IGSF8 | 7226 | TTC26 | 8694 | TOB1 |
| 1355 | PPM1G | 2823 | LOC339442 | 4291 | PDE3B | 5759 | CTC-360P9.3 | 7227 | GAA | 8695 | SLC6A16 |
| 1356 | SPCS1 | 2824 | PLA2G2E | 4292 | MCTP2 | 5760 | RCBTB1 | 7228 | EFNA3 | 8696 | ODF3B |
| 1357 | UBXN8 | 2825 | GPR113 | 4293 | RNF31 | 5761 | GPR141 | 7229 | LOC101927924 | 8697 | CNIH3 |
| 1358 | ZDHHC15 | 2826 | ATL2 | 4294 | IMMP1L | 5762 | WRNIP1 | 7230 | IPO5 | 8698 | CXCL9 |
| 1359 | DDX42 | 2827 | LOC100131860 | 4295 | SIK3 | 5763 | UNG | 7231 | SLC23A3 | 8699 | RNF144B |
| 1360 | PDZD11 | 2828 | LOC100128571 | 4296 | ANXA6 | 5764 | LOC101928758 | 7232 | LRRFIP1 | 8700 | CFB |
| 1361 | C6ORF35 | 2829 | LOC645586 | 4297 | GTF2IRD1 | 5765 | HCN3 | 7233 | KCNH2 | 8701 | CDK6 |
| 1362 | CCDC80 | 2830 | LOC100133308 | 4298 | HSPB8 | 5766 | PADI4 | 7234 | CASKIN1 | 8702 | CAMP |
| 1363 | TIMM44 | 2831 | C22ORF29 | 4299 | GAS7 | 5767 | ZBTB4 | 7235 | TRHDE | 8703 | RASGRP3 |
| 1364 | EMP1 | 2832 | GFOD1 | 4300 | CPS1 | 5768 | PLA2G4B | 7236 | KIF15 | 8704 | ZDHHC2 |
| 1365 | NT5DC3 | 2833 | TRIM11 | 4301 | DUSP4 | 5769 | RPL31 | 7237 | ELMO2 | 8705 | TARP |
| 1366 | TNFAIP3 | 2834 | FAM169A | 4302 | YJEFN3 | 5770 | MUC16 | 7238 | LOC100130691 | 8706 | RHOH |
| 1367 | LOC728723 | 2835 | WFDC11 | 4303 | YWHAH | 5771 | SEMA4C | 7239 | PCED1B | 8707 | CD99P1 |
| 1368 | AMOT | 2836 | KY | 4304 | METTL17 | 5772 | LOXHD1 | 7240 | SYNJ1 | 8708 | TMEM14A |
| 1369 | MPDU1 | 2837 | RCOR2 | 4305 | SETD5 | 5773 | SI | 7241 | FAM228A | 8709 | PCNX1 |
| 1370 | C20ORF151 | 2838 | SLIT3 | 4306 | ZC3H12C | 5774 | FAM159A | 7242 | SORL1 | 8710 | GBP4 |
| 1371 | CXCL6 | 2839 | DLG2 | 4307 | GSR | 5775 | HOMER3 | 7243 | STIM2 | 8711 | KL |
| 1372 | NOP56 | 2840 | EFNB1 | 4308 | FYN | 5776 | LBR | 7244 | MRVI1-AS1 | 8712 | RCAN2 |
| 1373 | DHRS7 | 2841 | LOC728743 | 4309 | MS4A6E | 5777 | ITGAX | 7245 | ARHGEF3 | 8713 | ID2B |
| 1374 | CDH23 | 2842 | NAA35 | 4310 | TUBGCP2 | 5778 | ANKZF1 | 7246 | ESR2 | 8714 | SLC39A10 |
| 1375 | VCAM1 | 2843 | FBXO47 | 4311 | CADM1 | 5779 | SETDB2 | 7247 | TPTEP1 | 8715 | GYPC |
| 1376 | AGFG2 | 2844 | LOC100131929 | 4312 | MAP4K4 | 5780 | RAB24 | 7248 | PNLDC1 | 8716 | CHST13 |
| 1377 | PET112 | 2845 | AURKAPS1 | 4313 | F8 | 5781 | UNC45A | 7249 | ACKR3 | 8717 | RSPO3 |
| 1378 | VAMP3 | 2846 | LOC387646 | 4314 | TNFRSF14 | 5782 | MTL5 | 7250 | DDR2 | 8718 | SSBP3 |
| 1379 | SS18L1 | 2847 | FAM27A | 4315 | LY9 | 5783 | SYNPR | 7251 | MMS22L | 8719 | CCL5 |
| 1380 | BUB1B | 2848 | SCARNA17 | 4316 | TPP1 | 5784 | UHRF1BP1L | 7252 | RPS6KB1 | 8720 | GUSBP9 |
| 1381 | HEXA | 2849 | ADRA1A | 4317 | SGSH | 5785 | SOX15 | 7253 | CUTA | 8721 | TRAC |
| 1382 | MYL6 | 2850 | OTUD6B | 4318 | RP11-20G13.2 | 5786 | SGCZ | 7254 | RALGPS2 | 8722 | LIMK2 |
| 1383 | EIF3G | 2851 | ATP2B3 | 4319 | EIF5 | 5787 | LOC399491 | 7255 | REXO1 | 8723 | LOC100996412 |
| 1384 | PTPLA | 2852 | METTL15 | 4320 | PHRF1 | 5788 | GPR161 | 7256 | TNPO2 | 8724 | HTRA4 |
| 1385 | CD9 | 2853 | TRDN | 4321 | RNF123 | 5789 | GPATCH2L | 7257 | IGDCC4 | 8725 | MCOLN2 |
| 1386 | WBP2 | 2854 | TNFAIP8L1 | 4322 | AP5Z1 | 5790 | GIPR | 7258 | LINC00971 | 8726 | CD69 |
| 1387 | PGR | 2855 | LOC100133311 | 4323 | MMP16 | 5791 | MSANTD3 | 7259 | ATAT1 | 8727 | IL2RB |
| 1388 | SNX16 | 2856 | GPRIN2 | 4324 | ARHGEF2 | 5792 | CCDC42B | 7260 | CNTN3 | 8728 | FPR2 |
| 1389 | PAFAH2 | 2857 | EXOG | 4325 | SATB1 | 5793 | SMAD3 | 7261 | MYO18A | 8729 | MLLT11 |
| 1390 | JHDM1D | 2858 | LOC100132006 | 4326 | CCR5 | 5794 | KAL1 | 7262 | NAB1 | 8730 | XCL2 |
| 1391 | ACTR1B | 2859 | PRDM8 | 4327 | SLC7A2 | 5795 | MMP15 | 7263 | COL18A1 | 8731 | CYB5R3 |
| 1392 | ANP32E | 2860 | TTC3 | 4328 | TDRD6 | 5796 | ZMYND12 | 7264 | MSI2 | 8732 | CD2 |
| 1393 | PLXNB1 | 2861 | MYO15B | 4329 | HK2 | 5797 | ZFC3H1 | 7265 | ANKRD16 | 8733 | ITPKB |
| 1394 | STK38 | 2862 | GPR133 | 4330 | SLC35F2 | 5798 | DENND4A | 7266 | DZANK1 | 8734 | MDFIC |
| 1395 | WDR74 | 2863 | ALMS1 | 4331 | RBM20 | 5799 | MCU | 7267 | BPIFB4 | 8735 | A2M |
| 1396 | CMTM7 | 2864 | N6AMT1 | 4332 | IPO11 | 5800 | CUEDC1 | 7268 | SLC26A6 | 8736 | LILRA2 |
| 1397 | EXD1 | 2865 | ATP1B2 | 4333 | ZNF331 | 5801 | LOC100996724 | 7269 | RP11-38L15.2 | 8737 | GBP5 |
| 1398 | RNF6 | 2866 | PPP1R42 | 4334 | PRSS23 | 5802 | CDON | 7270 | PPARGC1A | 8738 | ICAM1 |
| 1399 | HEXB | 2867 | TNS1 | 4335 | ELMSAN1 | 5803 | UROD | 7271 | SERF2 | 8739 | CYGB |
| 1400 | PPPDE2 | 2868 | ANKRD20A2 | 4336 | TGM3 | 5804 | EMC1 | 7272 | TRIM22 | 8740 | DDIT4L |
| 1401 | NOSIP | 2869 | SPEG | 4337 | SSH1 | 5805 | SERGEF | 7273 | MYO1A | 8741 | AIM2 |
| 1402 | NDUFC2 | 2870 | TKTL1 | 4338 | TBC1D19 | 5806 | VPS36 | 7274 | ARHGEF28 | 8742 | LINC00969 |
| 1403 | PPP1R15A | 2871 | EFR3B | 4339 | ACAD10 | 5807 | LOC100129603 | 7275 | DUS3L | 8743 | PSTPIP2 |
| 1404 | LOC729313 | 2872 | LOC644339 | 4340 | ALDH6A1 | 5808 | IRF9 | 7276 | SLC5A9 | 8744 | ISG20 |
| 1405 | RAC2 | 2873 | OR4A5 | 4341 | PLEKHA7 | 5809 | C2ORF81 | 7277 | PMS1 | 8745 | XCL1 |
| 1406 | H6PD | 2874 | C9ORF71 | 4342 | IRF7 | 5810 | FLJ43879 | 7278 | ADAM22 | 8746 | TNFSF10 |
| 1407 | BRF2 | 2875 | ADHFE1 | 4343 | AGPAT9 | 5811 | ITGA9 | 7279 | ANKRD26P3 | 8747 | HCP5 |
| 1408 | DDX24 | 2876 | SIGLEC16 | 4344 | TBK1 | 5812 | DPH7 | 7280 | CD33 | 8748 | HS3ST1 |
| 1409 | PRKCH | 2877 | PPP4R1L | 4345 | REC8 | 5813 | CYTIP | 7281 | MIA-RAB4B | 8749 | RCBTB2 |
| 1410 | GALNT1 | 2878 | IL18R1 | 4346 | DOT1L | 5814 | RP1-140K8.1 | 7282 | TEP1 | 8750 | ACOD1 |
| 1411 | LOC157627 | 2879 | TMOD2 | 4347 | AMPD3 | 5815 | ACOT6 | 7283 | CORO2A | 8751 | STAT1 |
| 1412 | C17ORF64 | 2880 | ZDHHC14 | 4348 | EYA1 | 5816 | SLC41A3 | 7284 | MFSD2A | 8752 | RNASE6 |
| 1413 | C1D | 2881 | WNT3A | 4349 | LINC01181 | 5817 | DQX1 | 7285 | SNAR-D | 8753 | APOL3 |
| 1414 | VDAC3 | 2882 | GPR97 | 4350 | F5 | 5818 | CEP131 | 7286 | MCF2L2 | 8754 | ALK |
| 1415 | MRPS23 | 2883 | GNAZ | 4351 | C2CD3 | 5819 | TRIM46 | 7287 | KLK3 | 8755 | BNIP3 |
| 1416 | NES | 2884 | WNK4 | 4352 | RAI14 | 5820 | UVSSA | 7288 | CTSW | 8756 | CCL23 |
| 1417 | ATP5G1 | 2885 | MGC12488 | 4353 | GALNT3 | 5821 | DCDC2 | 7289 | METRN | 8757 | GPR34 |
| 1418 | HAUS8 | 2886 | IFT74 | 4354 | ZFAND2A | 5822 | POLR3E | 7290 | ZNF461 | 8758 | C16ORF54 |
| 1419 | IFT46 | 2887 | LOC283270 | 4355 | MMS19 | 5823 | TGFA | 7291 | CCDC121 | 8759 | RARRES3 |
| 1420 | SPAG1 | 2888 | PIP4K2A | 4356 | IL18BP | 5824 | GALNT5 | 7292 | ABCB9 | 8760 | SOCS2 |
| 1421 | G6PC3 | 2889 | FLJ45513 | 4357 | CCHCR1 | 5825 | CCDC51 | 7293 | FPGS | 8761 | B4GALT1 |
| 1422 | PDK3 | 2890 | HNRNPA1L2 | 4358 | ATG2A | 5826 | SNX8 | 7294 | KDM2B | 8762 | LOC644090 |
| 1423 | UBE2B | 2891 | EPHA7 | 4359 | TPK1 | 5827 | CMPK2 | 7295 | BPIFB3 | 8763 | CD274 |
| 1424 | COG3 | 2892 | SSTR4 | 4360 | ABCA10 | 5828 | E2F6 | 7296 | PEAR1 | 8764 | CCL4 |
| 1425 | RBM19 | 2893 | DNAJC14 | 4361 | EPB41L5 | 5829 | EDN1 | 7297 | LRRC28 | 8765 | PTH2R |
| 1426 | ZMAT3 | 2894 | CNOT4 | 4362 | PRLR | 5830 | ITGB1 | 7298 | LRMP | 8766 | SLC18B1 |
| 1427 | SNRPB | 2895 | ARHGAP32 | 4363 | SLC12A8 | 5831 | SUV420H1 | 7299 | LOC152225 | 8767 | HSPA6 |
| 1428 | MAD2L1BP | 2896 | LOC100506190 | 4364 | SMIM3 | 5832 | RP11-638I2.8 | 7300 | PAQR5 | 8768 | CD180 |
| 1429 | PAIP1 | 2897 | WHSC1 | 4365 | ERCC6 | 5833 | OSBP2 | 7301 | EXOC4 | 8769 | PPP5D1 |
| 1430 | DCAKD | 2898 | SPIC | 4366 | MTSS1 | 5834 | NUP214 | 7302 | NPR3 | 8770 | ENTHD1 |
| 1431 | KHDRBS3 | 2899 | ZNF763 | 4367 | PCM1 | 5835 | ISY1-RAB43 | 7303 | TRPC6 | 8771 | CXCL10 |
| 1432 | ABL2 | 2900 | ZNF503-AS2 | 4368 | LIPN | 5836 | ITPKA | 7304 | SLC27A1 | 8772 | ATP9A |
| 1433 | NCAPD3 | 2901 | STARD9 | 4369 | KIAA1324 | 5837 | RP4-568F9.6 | 7305 | CALCOCO2 | 8773 | MIR3945HG |
| 1434 | RNF130 | 2902 | NBEAL2 | 4370 | PLCD1 | 5838 | OTOG | 7306 | MIS18BP1 | 8774 | UBD |
| 1435 | UBR3 | 2903 | FAM151A | 4371 | FMNL3 | 5839 | CACNA1F | 7307 | ONECUT1 | 8775 | LOC105378749 |
| 1436 | WDYHV1 | 2904 | TNXB | 4372 | KCNH4 | 5840 | C22ORF24 | 7308 | SNORD50A | 8776 | LOC101928916 |
| 1437 | DRAM1 | 2905 | IL7R | 4373 | LOC100130256 | 5841 | SLC22A23 | 7309 | TMX2 | 8777 | CES1P1 |
| 1438 | MDM2 | 2906 | LOC100129884 | 4374 | PTPN7 | 5842 | AOC1 | 7310 | MEGF11 | 8778 | HEY1 |
| 1439 | ZNF202 | 2907 | NEDD4L | 4375 | SYCP1 | 5843 | RGS11 | 7311 | RBFOX3 | 8779 | IDO1 |
| 1440 | GINS2 | 2908 | ARHGEF33 | 4376 | RHOBTB3 | 5844 | DNAH1 | 7312 | KIAA0319 | 8780 | ZNF366 |
| 1441 | IFI35 | 2909 | LOC283585 | 4377 | SLC26A11 | 5845 | PCGF2 | 7313 | NOX4 | 8781 | EHD1 |
| 1442 | FUT6 | 2910 | LENG8 | 4378 | HMCN1 | 5846 | MRS2 | 7314 | KAT2A | 8782 | MSC-AS1 |
| 1443 | ATG5 | 2911 | LOC284628 | 4379 | ADAM19 | 5847 | GMDS-AS1 | 7315 | GPD1 | 8783 | KIAA0101 |
| 1444 | TNFRSF1A | 2912 | PCDHGA3 | 4380 | TXNRD1 | 5848 | SERINC2 | 7316 | FAM221A | 8784 | PLTP |
| 1445 | PHB2 | 2913 | UBE2I | 4381 | KCNT2 | 5849 | DHFRL1 | 7317 | SLC14A2 | 8785 | ADGRG2 |
| 1446 | LONP1 | 2914 | ITGA10 | 4382 | RGL4 | 5850 | BEND5 | 7318 | MYO16 | 8786 | LOC102723899 |
| 1447 | STARD4 | 2915 | IHH | 4383 | IL1RN | 5851 | DNAH9 | 7319 | SLC4A9 | 8787 | TDRD3 |
| 1448 | SPRY1 | 2916 | OR1S1 | 4384 | POLR2A | 5852 | SHROOM1 | 7320 | NR3C1 | 8788 | MARCKS |
| 1449 | LACTB2 | 2917 | FAM22F | 4385 | RP11-283G6.5 | 5853 | GTF3A | 7321 | NUBPL | 8789 | GNG11 |
| 1450 | RNF19A | 2918 | KCNB1 | 4386 | TTC5 | 5854 | RENBP | 7322 | PTPRM | 8790 | CHI3L1 |
| 1451 | RAB27A | 2919 | SPAG11B | 4387 | PMP22 | 5855 | NELFB | 7323 | SLC1A1 | 8791 | RRM2 |
| 1452 | ABCC2 | 2920 | RPL13P5 | 4388 | TG | 5856 | MORC1 | 7324 | XYLB | 8792 | SLC1A2 |
| 1453 | LYRM7 | 2921 | TMEM154 | 4389 | ROS1 | 5857 | TMEM161B-AS1 | 7325 | MYRF | 8793 | ITGB8 |
| 1454 | GBAP1 | 2922 | LRRC37B | 4390 | HES4 | 5858 | CCNA1 | 7326 | TRIP4 | 8794 | RASGRP1 |
| 1455 | ALDH1A3 | 2923 | C21ORF90 | 4391 | RGL1 | 5859 | RIMS1 | 7327 | ALDH1A2 | 8795 | GPAT3 |
| 1456 | KHDC1 | 2924 | LUZP4 | 4392 | IFT88 | 5860 | VAC14 | 7328 | PECR | 8796 | GZMB |
| 1457 | DHX9 | 2925 | LOC100131101 | 4393 | CHST15 | 5861 | CAPN6 | 7329 | MARK4 | 8797 | LOC101928429 |
| 1458 | PDS5A | 2926 | SPDYC | 4394 | UGDH | 5862 | RHBG | 7330 | DTWD2 | 8798 | LOC101928635 |
| 1459 | VPS4B | 2927 | KRBA2 | 4395 | FAM129B | 5863 | NKIRAS2 | 7331 | MARK3 | 8799 | IFI44L |
| 1460 | AACS | 2928 | ZAN | 4396 | DOCK3 | 5864 | ZNF652 | 7332 | PRKAR1B | 8800 | HAMP |
| 1461 | PLS1 | 2929 | LOC144571 | 4397 | SLC36A1 | 5865 | BMP7 | 7333 | RNU5A-8P | 8801 | UCK2 |
| 1462 | CYB5A | 2930 | SCGB1D2 | 4398 | CREBZF | 5866 | NPEPPS | 7334 | AF124730.4 | 8802 | TRGV9 |
| 1463 | EID2 | 2931 | LOC644656 | 4399 | NFATC1 | 5867 | COL6A4P2 | 7335 | ATPAF1 | 8803 | ID2 |
| 1464 | ZFAND5 | 2932 | MLL5 | 4400 | ANKH | 5868 | CNIH4 | 7336 | CTNNBL1 | 8804 | GUSBP3 |
| 1465 | 3-Mar | 2933 | SCARF1 | 4401 | SLC9A7 | 5869 | KIF21B | 7337 | ZNF212 | 8805 | CCL15 |
| 1466 | SDC4 | 2934 | NBR2 | 4402 | ATP2A3 | 5870 | NDUFB8 | 7338 | C20ORF166-AS1 | 8806 | TRGC2 |
| 1467 | PRODH | 2935 | ACTRT1 | 4403 | CTD-2033D15.1 | 5871 | NUP62 | 7339 | BIN1 |  |  |
| 1468 | CA2 | 2936 | ELK4 | 4404 | LGALS8 | 5872 | NSL1 | 7340 | CACNB1 |  |  |

Table S4. Results of KEGG functional enrichment of common targets of ISOF and COPD in network pharmacology

| Category | Term | Count | PValue |
| --- | --- | --- | --- |
| KEGG | hsa05200:Pathways in cancer | 13 | 1.37E-05 |
| KEGG | hsa04068:FoxO signaling pathway | 7 | 3.43E-04 |
| KEGG | hsa04914:Progesterone-mediated oocyte maturation | 6 | 3.55E-04 |
| KEGG | hsa05215:Prostate cancer | 6 | 3.74E-04 |
| KEGG | hsa04015:Rap1 signaling pathway | 8 | 6.45E-04 |
| KEGG | hsa04114:Oocyte meiosis | 6 | 0.001082793 |
| KEGG | hsa04520:Adherens junction | 5 | 0.001555405 |
| KEGG | hsa05205:Proteoglycans in cancer | 7 | 0.002763877 |
| KEGG | hsa04912:GnRH signaling pathway | 5 | 0.003855459 |
| KEGG | hsa04913:Ovarian steroidogenesis | 4 | 0.00497234 |
| KEGG | hsa04915:Estrogen signaling pathway | 5 | 0.005209222 |
| KEGG | hsa04923:Regulation of lipolysis in adipocytes | 4 | 0.007225262 |
| KEGG | hsa00590:Arachidonic acid metabolism | 4 | 0.009150841 |
| KEGG | hsa05169:Epstein-Barr virus infection | 5 | 0.010788387 |
| KEGG | hsa04110:Cell cycle | 5 | 0.011404074 |
| KEGG | hsa04510:Focal adhesion | 6 | 0.015020225 |
| KEGG | hsa05161:Hepatitis B | 5 | 0.019267505 |
| KEGG | hsa04921:Oxytocin signaling pathway | 5 | 0.021534316 |
| KEGG | hsa04014:Ras signaling pathway | 6 | 0.021570909 |
| KEGG | hsa04750:Inflammatory mediator regulation of TRP channels | 4 | 0.032214752 |
| KEGG | hsa04010:MAPK signaling pathway | 6 | 0.033049605 |
| KEGG | hsa05219:Bladder cancer | 3 | 0.034167463 |
| KEGG | hsa04151:PI3K-Akt signaling pathway | 7 | 0.034550378 |
| KEGG | hsa05152:Tuberculosis | 5 | 0.036563247 |
| KEGG | hsa04726:Serotonergic synapse | 4 | 0.044080035 |
| KEGG | hsa04919:Thyroid hormone signaling pathway | 4 | 0.048115266 |
| KEGG | hsa00330:Arginine and proline metabolism | 3 | 0.049029185 |

Table S5. Basic information of the top 20 targets in the PPI network based on DEGs in ISOF vs M in three algorithms Degree, Betweenness Centrality and Closeness Centrality respectively (bold indicates the most important genes)

| NO. | Name | Degree | Name | BetweennessCentrality |
| --- | --- | --- | --- | --- |
| 1 | ***Tp53*** | 38 | *Vwa1* | 1 |
| 2 | ***Lck*** | 32 | *C6* | 1 |
| 3 | ***Il2rb*** | 23 | *Kcnj4* | 1 |
| 4 | *Cd247* | 21 | *Lrrc23* | 1 |
| 5 | *Grap2* | 21 | *Snrpg* | 0.66667 |
| 6 | ***Mmp9*** | 19 | *LOC108349606* | 0.58333 |
| 7 | ***Itgb2*** | 19 | ***Tp53*** | 0.36837 |
| 8 | *Il2rg* | 19 | ***Itgb2*** | 0.14213 |
| 9 | ***Ccl5*** | 19 | *Cyp2e1* | 0.14 |
| 10 | *Jak3* | 18 | ***Lck*** | 0.12882 |
| 11 | *Lat* | 18 | ***Mmp9*** | 0.12008 |
| 12 | *RT1-CE5* | 17 | *Glul* | 0.09989 |
| 13 | *RT1-CE15* | 17 | *Hdac6* | 0.08926 |
| 14 | *RT1-A2* | 17 | *LOC100361854* | 0.08333 |
| 15 | *Itk* | 17 | *H6pd* | 0.05888 |
| 16 | *Gata3* | 15 | *Gata3* | 0.05881 |
| 17 | *Grap* | 15 | *Alas2* | 0.05858 |
| 18 | *Thy1* | 14 | ***Il2rb*** | 0.05846 |
| 19 | *Klrk1* | 14 | *Cp* | 0.04809 |
| 20 | *RT1-Bb* | 13 | ***Ccl5*** | 0.0454 |
